# Asparagusic Golgi Trackers

**DOI:** 10.1021/jacsau.4c00487

**Published:** 2024-08-20

**Authors:** Saidbakhrom Saidjalolov, Xiao-Xiao Chen, Julia Moreno, Michael Cognet, Luis Wong-Dilworth, Francesca Bottanelli, Naomi Sakai, Stefan Matile

**Affiliations:** § Department of Organic Chemistry, University of Geneva, CH-1211 Geneva, Switzerland; ‡ Institute for Chemistry and Biochemistry, Freie Universität Berlin, Thielallee 63, D-14195 Berlin, Germany

**Keywords:** Fluorescent probes, Golgi trackers, bioimaging, fluorescent flippers, cellular uptake, thiol-mediated uptake, palmitoylation

## Abstract

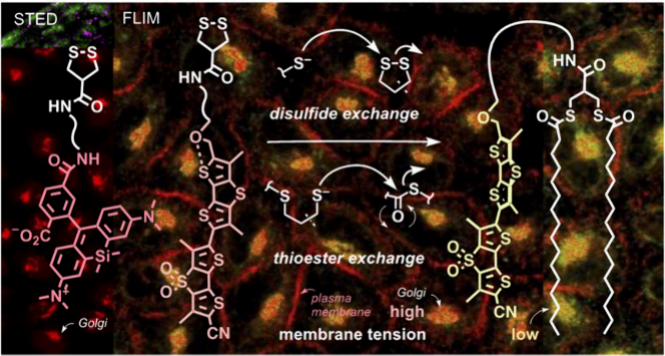

Thiol-mediated uptake (TMU) is thought to occur through dynamic covalent cascade exchange networks. Here we show that the cascade accounting for TMU of asparagusic acid derivatives (AspA) ends in the Golgi apparatus (G) and shifts from disulfide to thioester exchange with palmitoyl transferases as the final exchange partner. As a result, AspA combined with pH-sensitive fluoresceins, red-shifted silicon-rhodamines, or mechanosensitive flipper probes selectively labels the Golgi apparatus in fluorescence microscopy images in living and fixed cells. AspA Golgi trackers work without cellular engineering and excel with speed, simplicity, generality, and compatibility with G/ER and cis/trans discrimination, morphological changes, anterograde vesicular trafficking, and superresolution imaging by stimulated emission depletion microscopy. Golgi flippers in particular can image membrane order and tension in the Golgi and, if desired, at the plasma membrane during TMU.

Thiol-mediated uptake (TMU) is emerging as an enigmatic process that enables cellular entry of substrates appended with thiol-reactive moieties for drug delivery and drug discovery.^1−13^ Efforts to decode the underlying thiol–disulfide exchange networks have focused on the identification of cellular exchange partners P_m_ that enable the penetration of the plasma membrane (PM) through toroidal elastics (Figure 1B, **I**–**III**).^1,2,14^ In clear contrast, the intracellular partners P_i_ at the end of TMU cascades remain essentially unexplored. In this study, we show that derivatives of asparagusic acid^15^ (AspA, **1**) accumulate in the Golgi apparatus to finish their thiol–disulfide-exchange-based TMU^1,2,14,16−19^ as doubly palmitoylated amphiphiles like **2**, the products of dynamic covalent thioester exchange cascades^20−25^ catalyzed by palmitoyl transferases (PATs)^26−32^ as intracellular partners P_i_ (Figure 1B, **IV**–**VI**).

**Figure 1:**
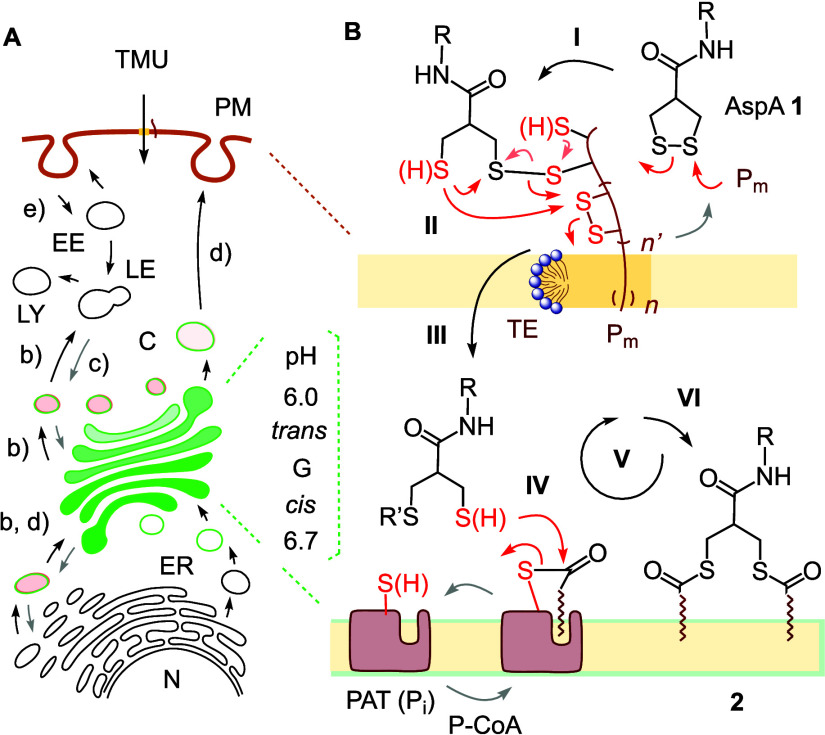
Thiol-mediated uptake, Golgi tracking, and vesicle trafficking. (A) Golgi apparatus (G) and vesicle trafficking, with endoplasmic reticulum (ER), nucleus (N), cytosol (C), lysosome (LY), late (LE) and early endosome (EE). (b) Anterograde and (c) retrograde transport (gray arrows), (d) secretory pathway, (e) endocytosis. (B) TMU of AspA **1** is found to end in the Golgi. Disulfide exchange cascades **I**–**III** with membrane-associated exchange partners (P_m_) to cross the plasma membrane (PM) through toroidal elastics (TE) are terminated by thioester exchange **IV**–**VI** with palmitoyl transferases (PATs) as intracellular P_i_.

The specific labeling of intracellular sites of interest, organelles and beyond, is fundamental for bioimaging. Small-molecule trackers are particularly attractive because they can be added to living systems without genetic engineering or immunofluorescence procedures limited to fixed cells.^33−40^ Most commercially available Golgi (G) trackers are sphingosine derivatives like **3** (Figure 2).^41−46^ They generally suffer from complex and slow application protocols and limited selectivity, particularly with regard to G/ER discrimination.^42,43^ Most recent progress with small-molecule Golgi trackers focused on cysteine derivatives.^42−54^ For the Tsukiji probe **4**, palmitoylation by PATs in the Golgi has been demonstrated.^42^

**Figure 2:**
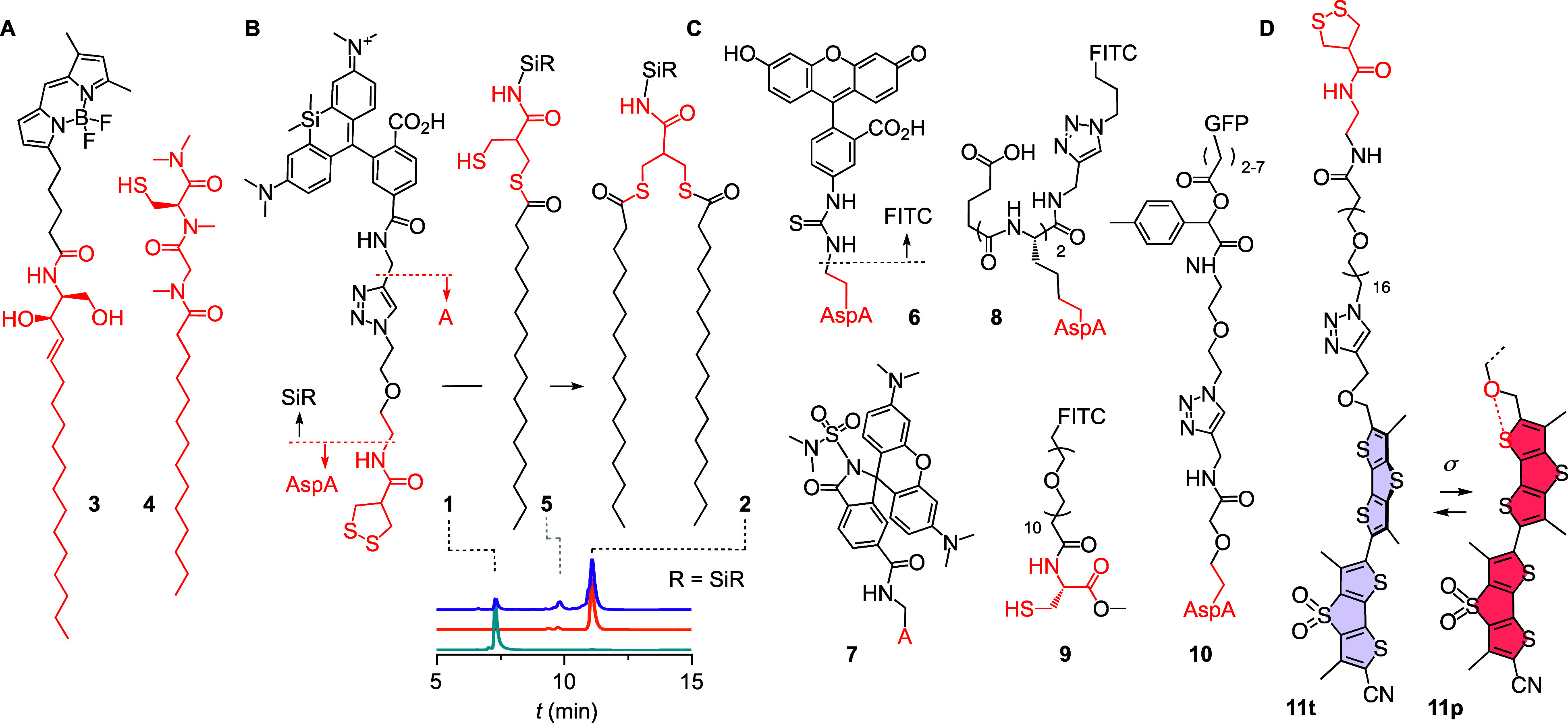
Structure and function of Golgi trackers. (A) Representative existing Golgi trackers. (B) SiR-AspA **1** with palmitoylation products **5** and **2** and HPLC traces of cell extracts (top) compared to pure **2** (middle) and **1** (bottom). (C) FITC-AspA **6** and controls (GFP = green fluorescent protein). (D) Golgi-Flipper **11**, with compression of twisted **11t** into planar **11p** by physical forces σ.

Isosteric of singly palmitoylated AspA **5**, the Tsukiji probe **4** supported that palmitoylation could fix AspA-conjugates, e.g., **1** as a double-tail amphiphile **2** in the Golgi (Figure 2). Such probes could be advantageous for Golgi tracking because superb cellular uptake of AspA via thiol–disulfide exchange is well established,^1,2,14,16−19^ while the two potentially precipitate-inducing greasy alkyl tails would be added only *in situ* at the site of interest (Figure 1B).

Asparagusates **1** and **6**–**8** were accessible from commercially available starting materials without extraordinary synthetic efforts (Figure 2, Schemes S1 and S2). Added to HK cells, FITC-AspA **6** (λ_ex_ 495 nm, λ_em_ 520 nm) labeled the Golgi in less than 10 min (Figure 3A; FITC: fluorescein thiourea). The brighter, red-shifted SiR-AspA **1** (Figure 3B, λ_ex_ 652 nm, λ_em_ 674 nm; SiR: silicon rhodamine^36,37,43,55,56^) and MaP555^57^-AspA **7** (Figures S8 and S10, λ_ex_ 555 nm, λ_em_ 580 nm) confirmed that Golgi tracking is fluorophore independent. The formation of hydrophobic palmitoylation products around^42^**2** and **5** could be detected in the HPLC of cell lysates (Figure 2B). The prepalmitoylated control **2** failed to label HK cells (Figure S11). This result confirmed the superiority of intracellular palmitoylation over prepalmitoylation for Golgi tracking (Figures 1B, 2).

**Figure 3:**
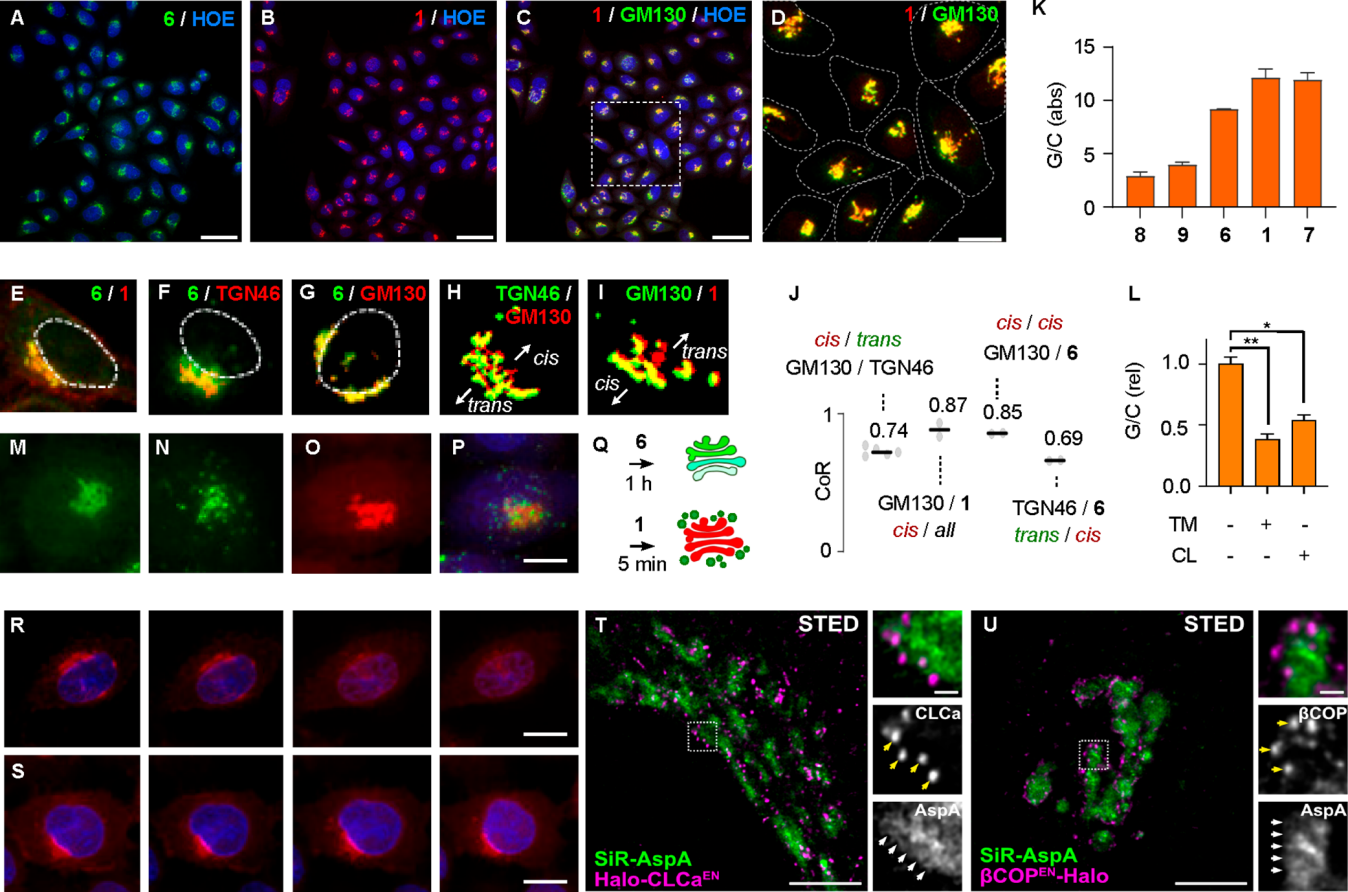
Evaluation of Golgi trackers. (A–G) SDCM images of fixed HK cells with (A) **6**, (B, C) **1**, (C) **1** and GM130, (D) zoom of (C) with cell boundaries (white dashed), (E) **6** (green, *cis*) and **1** (red, *all*), (F) **6** (green, *cis*) and TGN46 (red, *trans*), (G) **6** (green, *cis*) and GM130 (red, *cis-median*) with nuclei boundaries (E–G, white dashed), (H, I) masks generated for (H) GM130 (red, *cis-median*) and TGN46 (green, *trans*), and (I) GM130 (green, *cis-median*) and **1** (red, *all*). (J) Colocalization ratios for (F–I). (K, L) Golgi/cytosol (G/C) ratios for (K) **1**, **6**–**9** and for (L) **1** treated with tunicamycin (TM) and cerulenin (CL). (M–Q) SDCM images of live HK cells for sequential tracking (Q) with **6** (green, 20 μM) for (M) 1 h, then (N) 5 min after the addition of (O) **1** (red, 500 nM, (P) = (N) + (O) merged). (R, S) SDCM images of live HK cells with **1** with (R) and without (S) brefeldin A for 0, 10, 30, 60 min (from left). (T, U) Live-cell STED images of HeLa cells with **1** (green) expressing endogenously tagged (T) CLCa^EN^-Halo and (U) βCOP^EN^-Halo labeled with JF_571_–CA (magenta). Blue: Hoechst 33342 (HOE), scale bars: 50 μm (A–C), 20 μm (D), 10 μm (M–S), 5 and 0.5 μm in the crops (T, U).

Images obtained with SiR-AspA **1** were as good as those from immunofluorescence of Golgi proteins^58−62^ (Figure 3C, D). However, immunofluorescence procedures require 3–5 h and work only in fixed cells, while Golgi tracking with SiR-AspA **1** takes 10 min and works also in living cells. Fixing of cells did not significantly affect brightness or location of AspA probes, consistent with their firm anchoring through palmitoylation (Figure S15).

As for established probes, Golgi tracking depended on probe concentration and incubation time (Figures S3–S10). At high concentrations, probes spread throughout the cells, probably due to oversaturation of the Golgi. FITC **6** was metabolized with *t*_50_ ∼ 1 h as expected, while SiR **1** had a lifetime of *t*_50_ ∼ 4 h (Figures S19, S20). AspA trackers were nontoxic (Figures S9, S10, S12, S16),^1,2,14,16−19^ universal (e.g., HK, MCF-7, RPE-1, A431, MDCK cells, Figure S16), affected neither by ≤10% serum (Figure S14) nor by 500 μM cysteine (Figure S12), and ≥4 times enhanced by preincubation with BSA (Figure S13).

Automated comparison of masks (e.g., Figure 3H, I) for high-content (HC) imaging microscopy was applied to secure high-accuracy colocalization ratios (CoR), comparable to Pearson correlation coefficients (PCC). The PCC = 0.86 ± 0.02 (CoR = 0.87 ± 0.07) of AspA-SiR **1** against GM130 immunofluorescence was outstanding compared to known trackers (Figure 3B–D). Spreading into the ER, commercial trackers like **3** suffer from a Golgi to ER intensity ratio G/ER = 2.0.^42^ G/ER = 6.0 of the Tsukiji probe **4** was considered a recent key breakthrough.^42^ With AspA trackers, the ER was invisible (for **1**, G/ER > 20, PCC with ER tracker = 0.41 ± 0.02).

Most background fluorescence came from the cytosol. Incubation of 5 μM FITC-AspA **6** for 30 min gave a Golgi to cytosol ratio G/C = 8 (Figure 3K). For SiR **1** and MaP555 **7**, this further improved to G/C = 12. Control compounds, AspA dimers **8** and a simple cysteine^42−45,47−54^ conjugate **9** were poor trackers with G/C < 4 (Figures 3K, 2, S4–S6). The previously reported GFP-AspA **10** did not track the Golgi (G/C ≪ 1).^18^ This lost attraction to the Golgi could confirm that the envisioned traceless protein delivery by cytosolic esterolysis was indeed successful.^18^

Colocalization of SiR **1** with FITC **6** was poor (CoR = 0.67, Figure 3E). This implied that **6** could selectively track *cis* Golgi (pH = 6.7) because the pH-sensitive FITC (p*K*_a_ = 6.4) is quenched in *trans* Golgi (pH = 6.0, Figure 1A) if a significant portion of the Golgi trackers faces toward the Golgi lumen, possibly implying flip-flop. Although difficult to prove, this interpretation was supported empirically by the similarly poor colocalization of *cis*/*median*-selective immunofluorescent antibody GM130,^58,59,62^ against the *trans*-selective TGN46 (CoR = 0.74, Figure 3H),^60,61^ and of **6** (*cis*) against TGN46 (*trans*, 0.69, Figure 3F), and also by the good colocalization of **6** (*cis*) with the less selective GM130 (*cis*-*median*, 0.85, Figure 3G) and GM130 with the less selective **1** (*all*, 0.87, Figure 3I).

Live-cell stimulated emission depletion (STED) images of SiR-AspA **1** confirmed compatibility of this new Golgi tracker with multicolor superresolution microscopy (Figure 3T, U).^62,63^ Labeling of cells expressing endogenously Halo-tagged clathrin (Halo-CLCa^EN^, EN = endogenous) and COPI (BetaCOP^EN^-Halo)^63^ showed that AspA **1** tracks both *cis* and *trans* Golgi and defines Golgi cisternae decorated by coat proteins at their rims/edges (Figures 3T, U; 1b, c).^63^

SiR-AspA **1** faithfully showed the morphological changes of the Golgi caused by brefeldin A^64^ (Figure 3R, S). With AspA **6** at higher concentrations, labeling changed within 1 h from the Golgi to clustered puncta spreading from the Golgi, as evidenced by pulse-chase type tracking after the addition (chase) of AspA **1** at low concentrations (Figure 3M–Q). Thus, at higher concentrations (low micromolar) and after a longer incubation time (∼1 h), AspA trackers report on anterograde vesicular trafficking from the Golgi to endo/lysosomes but not to the PM (Figure 1A, b). This differed from lower concentrations (nanomolar), where AspA trackers reported from the Golgi membrane for hours without change (Figure 3S). Since palmitoylation is involved in anterograde transport (Figure 1A, b),^27^ these findings supported that Golgi tracking by AspA operates indeed by dynamic covalent thioester exchange (Figures 1, 2). Further support for operational palmitoylation of AspA in the Golgi could be obtained with PAT inhibitors tunicamycin (TM)^65^ and cerulenin (CL).^66^ The G/C ratio of **1** decreased in their presence, with the former by more than 50% (Figure 3L).

Fluorescent flippers have been introduced over the past decade as small molecule probes to image physical forces, that is, membrane tension, in living systems.^40,67−70^ To explore AspA trackers for flipper research, Golgi flipper **11** was synthesized (Scheme S3). While emission from the Golgi was qualitatively obvious, colocalization of **11** with **3** was technically challenging by confocal laser scanning microscopy (CLSM) because of bleed-through fluorescence (Figure 4A, F). However, in fluorescence lifetime imaging microscopy (FLIM) phasor hemispheres,^71−73^ τ = 3.7 ns of flipper **11** appeared well separated from τ = 5.1 ns of BODIPY **3** (Figure 4C, H). FLIM images mapped from the phasor hemispheres for HK cells labeled with both probes localized the origin of both lifetime maxima in the Golgi (Figures 4B, G).

**Figure 4:**
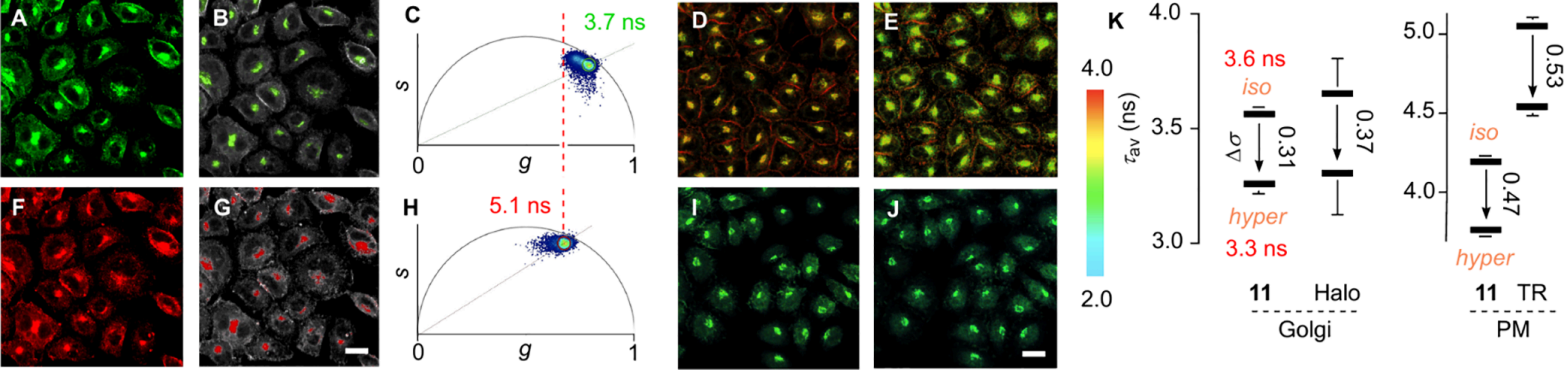
Golgi flippers. Comparison of HK cells with either **11** (A–E, λ_ex_ = 480 nm, detection 600–650 nm), **3** (F–H, λ_ex_ = 480 nm, detection 510–540 nm), or **1** (I, J, λ_ex_ = 600 nm, detection 630–800 nm) in (A, F) CLSM images, (B, G) FLIM images reconstructed from their (C, H) phasor hemispheres, (D, I) FLIM images under isoosmotic and (E, J) hyperosmotic conditions; scale bars: 20 μm. (K) Lifetimes and changes from isoosmotic to hyperosmotic HK cells (arrows, Δσ) for **11** in Golgi compared to HaloFlippers in Golgi and **11** in PM compared to Flipper-TR.

Physical compression of the favored twisted conformer **11t** into the planar conformer **11p** reports an increase in membrane order or tension with red-shifted emission and longer lifetimes (Figure 2).^40,67−69,74,75^ Under hyperosmotic stress, the lifetime of **11** in the Golgi decreased from τ_av_ = 3.6 ns by Δτ_av_ = −0.3 ns to τ_av_ = 3.3 ns (Figure 4D, E, K). Lifetime and response to reduced membrane tension were in the range of Halo-Flippers attached to sialyltransferases in the Golgi by genetic engineering (Figure 4K, Halo).^76^ As expected, SiR-AspA **1** was insensitive to changes in membrane tension (Δτ_av_ < 0.1 ns, Figure 4I, J).

Flippers **11** were partially retained in the plasma membrane (PM, Figure 4D, E), unlike hydrophilic probes like FITC or SiR (Figure 3A-D). This suggested that hydrophobically matching fluorophores like flippers could partially interrupt TMU at the PM and remain attached to the transferrin receptor^14^ or other P_m_ (Figure 1).^2^ The τ_av_ = 4.2 ns of **11** in the PM (Figure 4D, K) was shorter than the τ_av_ = 5.1 ns of the original Flipper TR^74,77^ (Figure 4K). These differences at preserved responsiveness to tension (Figure 4E, K) suggested that the local membrane environment of P_m_ during TMU could be less ordered than the rest of the PM. Transporter-flipper conjugates could thus possibly provide insights on local changes in membrane architecture during TMU, including toroidal elastics (Figure 1).^2^

In summary, this study identifies palmitoyl transferases in the Golgi as final partners in the exchange cascades that account for TMU of asparagusates, thus ending up with a shift from disulfide to dynamic covalent thioester exchange. Established early on as unique monomers to directionally grow functional dynamer surface architectures^78−80^ and to open up new entries into cells,^1,16^ their emergence as Golgi trackers endorses the asparagusic acid motif^15^ as a truly privileged scaffold, tiny but full of positive surprises.

## Methods

### Tracker Synthesis

To a solution of the NHS-ester of asparagusic acid^16^ (150 mg, 607 μmol, **12**) in dry CH_2_Cl_2_ (50 mL) was added 2-(2-azidoethoxy)ethan-1-amine^81^ (152 mg, 910 μmol, HCl salt, **13**) and DIPEA (429 μL, 2.43 mmol). The reaction mixture was stirred at rt overnight. Then it was quenched with 1 M HCl. The crude product was extracted with CH_2_Cl_2_ (x3), washed with brine, dried over Na_2_SO_4_ and concentrated *in vacuo*. The crude product was subjected to purification by flash chromatography (Scorpius silica 12 g, CH_2_Cl_2_/MeOH 95:5) to afford the desired amide (155 mg, 97%, **14**) as a colorless solid. *R*_f_ (CH_2_Cl_2_/MeOH 95:5): 0.52; Mp: 54–55 °C; IR (neat): 3296 (br, NH), 2101 (s, N_3_), 1637 (s, C=O), 1551 (s, N–C=O), 1297 (m), 1250 (s), 1112 (s, C–O–C), 1079 (s, C–O–C), 1045 (m, C–O), 683 (s, C–S); ^1^H NMR (400 MHz, CDCl_3_): 6.17 (s, 1H), 3.70 (t, ^3^*J*_H–H_ = 4.0 Hz, 2H), 3.58 (t, ^3^*J*_H–H_ = 5.0 Hz, 2H), 3.51 (q, ^3^*J*_H–H_ = 5.0 Hz, 2H), 3.40–3.37 (m, 4H), 3.36 (s, 2H), 3.16 (p, ^3^*J*_H–H_ = 7.0 Hz, 1H); ^13^C NMR (126 MHz, CDCl_3_): 171.8 (C), 70.4 (CH_2_), 69.8 (CH_2_), 52.6 (CH), 50.8 (CH_2_), 42.8 (2CH_2_), 39.6 (CH_2_); LRMS (ESI): 263 (C_8_H_15_N_4_O_2_S_2_, [M + H]^+^).

To a mixture of azide **14** (7.2 mg, 28 μmol) and alkyne **15** (7.0 mg, 14 μmol) in dry THF (5.0 mL) under N_2_ atmosphere was added a premixed solution of CuI (5.2 mg, 28 μmol) and THPTA (6.0 mg, 14 μmol) in dry THF. The reaction mixture was stirred at rt for 30 h (completion evidenced by LC-MS). The crude mixture was filtered to remove the excess of CuI and then the filtrate was concentrated *in vacuo*. RP-chromatography (Scorpius C18 33g, H_2_O + 0.1% TFA/CH_3_CN + 0.1% TFA, 1:1) afforded **1** (4.7 mg, 44%) as a blue solid. Mp: 120–121 °C; IR (neat): 2923 (br, NH), 1658 (s, C=O), 1575 (s, N–C=O), 1353 (s, C–N), 1314 (s, C–N), 1124 (s, Si(CH_3_)_2_), 922 (w, CH), 835 (m, Si–C), 719 (w); ^1^H NMR (500 MHz, DMSO-*d*_6_): 9.30 (t, ^3^*J*_H–H_ = 5.7 Hz, 1H), 8.16–8.08 (m, 2H), 8.04 (d, ^3^*J*_H–H_ = 8.0 Hz, 1H), 7.94 (s, 1H), 7.72 (s, 1H), 7.04 (s, 2H), 6.76–6.51 (m, 4H), 4.47–4.45 (m, 4H), 3.76 (t, ^3^*J*_H–H_ = 5.3 Hz, 2H), 3.40 (t, ^3^*J*_H–H_ = 5.7 Hz, 2H), 3.36–3.29 (m, 2H), 3.18 (q, ^3^*J*_H–H_ = 5.7 Hz, 2H), 3.15–3.09 (m, 3H), 2.93 (s, 12H), 0.64 (s, 3H), 0.52 (s, 3H); ^13^C NMR (126 MHz, DMSO-*d*_6_): 170.5 (C), 169.2 (C), 164.7 (C), 158.2 (C), 154.7 (C), 149.2 (2C), 144.3 (C), 139.1 (C), 136.0 (2C), 130.5 (C), 128.3 (2CH), 127.7 (2CH), 125.4 (C), 123.5 (CH), 123.0 (CH), 116.5 (2CH), 113.9 (2CH), 91.3 (C), 68.6 (CH_2_), 68.4 (CH_2_), 51.3 (CH), 49.2 (CH_2_), 42.1 (2CH_2_), 40.0 (4CH_3_), 38.7 (CH_2_), 35.0 (CH_2_), 0.0 (CH_3_), −1.4 (CH_3_); HRMS (ESI, + ve) calcd for C_38_H_45_N_7_O_5_S_2_Si [M + Na]^+^: 794.2585, found: 794.2574.

Trackers other than SiR-AspA **1** were synthesized analogously, see Supporting Information.

### Golgi Tracking

Human cervical cancer-derived HeLa Kyoto, human breast cancer-derived MCF-7, human retinal pigment epithelial-1 (RPE-1), Madin-Darby canine kidney (MDCK) and human epidermoid carcinoma (A-431) cells were cultured in complete FDMEM, i.e., FluoroBrite DMEM (GlutaMAX, 4.5 g/L d-glucose), supplemented with 10% fetal calf serum (FCS) and 1% Penicillin/Streptomycin (PS). HeLa CCL-2 (ECACC General Collection) were cultivated in DMEM supplemented with 10% FBS and 1% PS. The cells were grown under 5% CO_2_ humidified atmosphere at 37 °C on a 75 cm^3^ tissue culture flask (TPD Corporation). Cells were harvested by treatment with 3 mL of phenol-red free TrypLE Express, followed by the addition of 10 mL of complete FDMEM at 37 °C. The cells were spun down at 1500 g for 3 min, resuspended in complete FDMEM, and plated according to the concentration needed. For uptake or inhibition experiments, the cells were seeded in a μ-Plate 96-well Black ibiTreat sterile at 12 000 cells/well (HK, RPE-1, A-431) or 18 000 cells/well (MCF-7, MDCK) in complete FDMEM and left incubating under 5% CO_2_ humidified atmosphere at 37 °C overnight.

From cells thus prepared in a 96 well plate, medium was removed, and cells were washed with PBS (3 × 3 mL/well) followed by fresh FDMEM serum-free medium (4 × 100 μL/well) using a plate washer (Biotek EL406), and kept in a 100 μL of the latter medium. Solutions of **1**, **2**, **6**–**9** (2–10 mM, DMSO) were diluted in FDMEM to give a solution at 3× final concentration, of which 50 μL was added to the well resulting in a final volume of 150 μL per well. The cells were incubated under 5% CO_2_ humidified atmosphere at 37 °C for the indicated time (5–120 min). Then, the cells were washed with PBS (3 × 3 mL/well) and the medium was exchanged with FDMEM keeping a final volume of 100 μL/well, and a solution of Hoechst 33342 (100 μg/mL) in PBS (50 μL/well) was added. After 10 min of incubation under 5% CO_2_ humidified atmosphere at 37 °C, cells were washed with PBS (3 × 3 mL/well) and kept in FDMEM (100 μL/well) for live cell imaging. For fixed cell imaging, the cells were treated with a solution of PFA 3% (70 μL/well) for 15 min at rt. The excess of PFA was removed by washing with PBS (9 × 3 mL/well). The distribution of fluorescent signals was captured on a IXM-C automated microscope with two channels, blue for Hoechst 33342 (377/50 nm excitation filter; 477/60 nm emission filter), and green (475/34 nm excitation filter; 536/40 nm emission filter, for **6**, **8**, and **9**), red (531/40 nm excitation filter; 593/40 nm emission filter, for **7**) or far red (620/50 nm; emission filter: 690/50 nm, for **1** or **2**). The rest of the parameters were adjusted according to the nature of the experiment. Duplicates were performed for each condition. The resulting images were automatically analyzed and quantified using the MetaXpress software.

## Supporting Information Available

Detailed experimental procedures, materials and methods, synthesis, SDCM procedures, colocalization ratios from automated HC imaging, FLIM procedures, membrane tension measurement, original images (PDF)
