# Supplementary material for: Asparagusic Golgi Trackers
Source: JACS Au. 2024 Aug 20;4(10):3759–65. doi: 10.1021/jacsau.4c00487 (PMC11522900; doi:10.1021/jacsau.4c00487)
Supplement: Supplementary file 1 — au4c00487_si_001.pdf [file au4c00487_si_001.pdf]

Supporting Information

Asparagusic Golgi Trackers

Saidbakhrom Saidjalolov,[§] Xiao-Xiao Chen,[§] Julia Moreno,[§] Michael Cognet,[§] Luis Wong-Dilworth,[‡] Francesca Bottanelli,[‡] Naomi Sakai[§] and Stefan Matile*,[§]

[§]Department of Organic Chemistry, University of Geneva, CH-1211 Geneva, Switzerland

[‡]Institute for Chemistry and Biochemistry, Freie Universität Berlin, Thielallee 63, D-14195 Berlin, Germany

*E-mail: stefan.matile@unige.ch

Table of Contents

1.	Materials and Methods	S4
2.	Synthesis	S7
3.	Spectroscopic Properties	S17
4.	Cell Culture	S18
5.	Golgi Tracking	S19
	5.1. General Procedure	S19
	5.2. Data Analysis	S19
	5.3. FITC-AspA	S20
	5.4. SiR-AspA and MaP555-AspA	S23
	5.5. SiR-AspA vs Dipalmitoylated SiR-AspA	S26
6.	Influence of Cysteine	S27
7.	Influence of BSA	S27
8.	Influence of Serum	S28
9.	Difference Between Live and Fixed Cells	S29
10.	Dependence on Cell Lines	S29
11.	Stability	S30
	11.1. Stock Solutions	S30
	11.2. Cells	S31
12.	Co-Localization	S32
	12.1. ER-Trackers	S32
	12.2. Anti-Golgi Antibodies	S33
	12.3. Data Analysis	S34
	12.4. Golgi Proteins	S37
	12.5. Summary Table	S40

13. Golgi Morphology S40

13.1. Disassembly S40

13.2. Fusion with ER S41

13.3. Vesicle Trafficking S42

14. Palmitoylation S44

15. Palmitoyl Transferase Inhibitors S46

15.1. General Procedure S46

15.2. Data Analysis S47

15.3. Influence on Golgi Tracking S47

15.4. Endocytosis Inhibitors S49

16. Confocal Laser Scanning Microscopy (CLSM) S50

17. Fluorescence Lifetime Imaging Microscopy (FLIM) S52

18. Live-Cell STED Imaging S54

19. Supporting References S55

20. NMR Spectra S57

1. Materials and Methods

As in reference S1, briefly, reagents for synthesis were purchased from Merck, Sigma-Aldrich, TCI, Broadpharm and Fluorochem. Salts of the best grade available from Fluka or Sigma-Aldrich were used as received. Compounds **15** and **16** were from Spirochrome. Phosphate buffered saline (PBS, pH = 7.4), Dulbecco's PBS complemented with calcium and magnesium (DPBS, 14040117), FluoroBrite DMEM (GlutaMAX, 4.5 g/L D-glucose, pyruvate, without phenol red) medium, penicillin-streptomycin, Fetal calf serum , TrypLE Express Enzyme, V96-MicroWell plates, Nunclon™ Sphera™ U-bottom 96-well sterile µ-plates, Hoechst 33342 (HOE, 10 mg/mL solution in water) and propidium iodide (PI, 1.0 mg/mL solution in water), Golgi tracker **3** (BODIPY™ FL C5-Ceramide complexed to BSA, B22650), the LysoTracker™ Red (DND-99, L7528) and the ER tracker™ red (BODIPY™ TR Glibenclamide, E34250) were obtained from Thermo Fisher Scientific. M-Plate 96-Well Black were obtained from Ibidi. Primary antibodies against GM130 (610822), TGN46 (ab174280) and GOLPH3 (ab98023) were purchased from BD Biosciences and Abcam. Brefeldin A (ab120299) was purchased from Abcam. Chlorpromazine (C8138), cytochalasin B (C6762) and methyl-β-cyclodextrin (C4555) were purchased from Sigma-Aldrich. Monensin (15760909) and wortmannin (10706642) were purchased from Thermo Fisher Scientific. Fluorescent labeled secondary antibodies Alexa Fluor® 488 (715-545-150) and 647 (715-605-150) AffiniPure Donkey Anti-Mouse IgG (H+L) and Alexa Fluor® 488 (711-545-152) and 647 (711-605-152) AffiniPure Donkey Anti-Rabbit IgG (H+L)) were purchased from Jackson ImmunoResearch. Tunicamycin was purchased from Cayman Chemicals and cerulenin was purchased from Focus Biomolecules. Analytical thin layer chromatography (TLC) was performed on silica gel 60 F254 (Merck, 0.2 mm) and visualized under a UV lamp at 254 nm. Column chromatography was carried out on silica gel 60 (SilicaFlash® P60, SILICYCLE, 230-400 mesh). Flash chromatography was performed on a Biotage Isolera™ Spektra or Selekt using pre-packed Scorpius cartridges (BGB). Melting points (Mp) were recorded on a Melting Point M-565 (BUCHI). Alpha-D values were

measured on a Polarimeter P-1030 (Jasco). IR spectra were recorded on a Perkin Elmer Spectrum Two™ FT-IR spectrometer (ATR, Golden Gate) and are reported as wavenumbers v in cm^{-1} with band intensities indicated as br (broad), s (strong), m (medium), w (weak). All ^{1}H and ^{13}C NMR spectra were recorded either on a Bruker 300, 400 or 500 MHz spectrometer at room temperature (25 °C) and are reported as chemical shifts (δ) in parts per million (ppm) with reference to the residual solvent peak (DMSO-d_6: 2.50/39.5 ppm; CD_3OD: 3.31/49.0 ppm; $CDCl_3$: 7.26/77.2 ppm). Spin multiplicities are reported as a singlet (s), doublet (d), and triplet (t) with coupling constants (J) given in Hz, or multiplet (m). Broad peaks are marked as br. ^{1}H and ^{13}C resonances were assigned with the aid of additional information from 1D and 2D NMR spectra (^{1}H-^{1}H COSY, DEPT 135, HSQC and HMBC). ESI-HRMS was performed on Xevo G2-S TOF (Waters). LC-MS (low resolution) analyses were performed on Advion Avant® UHPLC system equipped with a Thermo C18 Hypersil GOLD column (50 × 2.1 mm, 1.9 µm particles size, 0.75 mL/min, gradient elution H_2O + 0.01% TFA / CH_3CN + 0.01% TFA 3:7 to 0:1 in 4.0 min) with Advion Expression® CMS in ESI mode. All mass data are reported as mass-per-charge ratio *m/z*. UV-Vis spectra were recorded on a JASCO V-650 spectrophotometer equipped with a stirrer and a temperature controller (20 ºC) and are reported as maximal absorption wavelength λ in nm (extinction coefficient ε in M^{-1} cm^{-1}). Fluorescence spectra were recorded on a FluoroMax-4 (Horiba Scientific). Fluorescence spectra were corrected with factors supplied by the manufacturer. Spinning disk confocal and wide field imaging of cells was performed using an IXM-C automated microscope from ImageXpress equipped with a Lumencor Aura III with 5 independent selectable solid-state light sources, bandpass filters and 5 objectives (4x to 60x). Washing steps were performed using a plate washer Biotek EL406®. Confocal laser scanning and fluorescence lifetime imaging was performed on Leica Stellaris FALCON equipped with 60x oil immersion objective.

Abbreviations. AHCHT: Automated high-content high-throughput; AspA: Asparagusic acid; BFA: Brefeldin A; BSA: Bovine serum albumin; CAX: Covalent exchangers; CL: Cerulenin; CLSM: Confocal laser scanning microscopy; CoR: Co-localization ratio; DIPEA: Diisopropylethylamine; DMEM: Dulbecco's modified eagle medium; DMF: *N*,*N*-dimethylformamide; DPBS: Dulbecco's phosphate-buffered saline; EDC: *N*-(3-Dimethylaminopropyl)-*N'*-ethylcarbodiimide; FCS: Fetal calf serum; FDMEM: FluoroBrite DMEM; FITC: Fluorescein isothiocyanate; FLIM: Fluorescence lifetime imaging microscopy; GA: Golgi apparatus; HATU: Hexafluorophosphate azabenzotriazole tetramethyl uronium; HBSS: Hanks' balanced salt solution; HeLa: Henrietta Lacks; HK: HeLa Kyoto; HPLC: High-Performance Liquid Chromatography; HRMS: High resolution mass spectra; LRMS: Low resolution mass spectra; MDCK: Madin-Darby canine kidney; PBS: Phosphate-buffered saline; PCC: Pearson correlation coefficient; PFA: Paraformaldehyde; PI: Propidium iodide; PS: Penicillin-streptomycin; ROI: Region of interest; RP: reverse-phase; RPE-1: human retinal pigment epithelial-1; rt: Room temperature; RV: Relative viability; SD: Standard deviation; SDCM: Spinning disk confocal microscopy; SEM: Standard error of mean; SiR: Silicon rhodamine; STED: Stimulated emission depletion; TBTA: Tris(benzyltriazolylmethyl)amine; TEA: Triethylamine; TGN: Trans Golgi network; THPP: Tris(hydroxypropyl)phosphine; THTPA: Tris(hydroxypropyltriazolyl-methyl)amine; TM: Tunicamycin; TMU: Thiol-mediated uptake; TRIS buffer: Tris(hydroxymethyl)aminoethane buffer; WI: Water immersion.

2. Synthesis

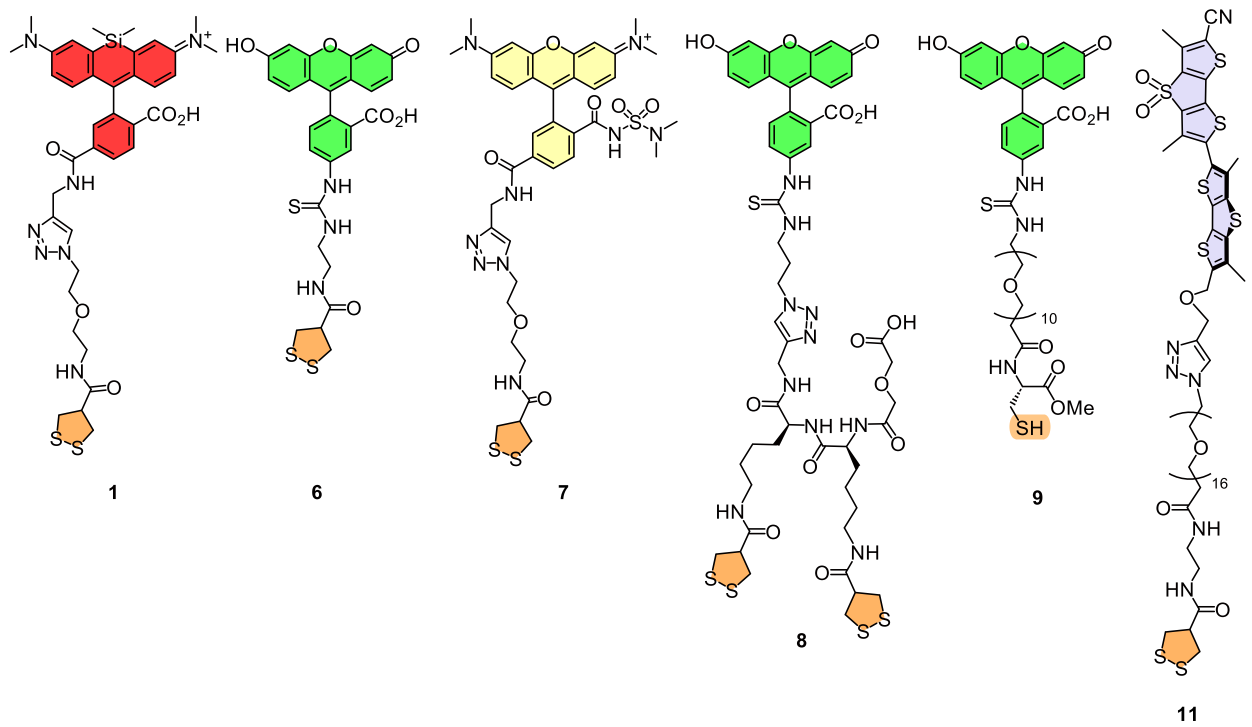

Figure S1. Structure of AspA derivatives used in this study.

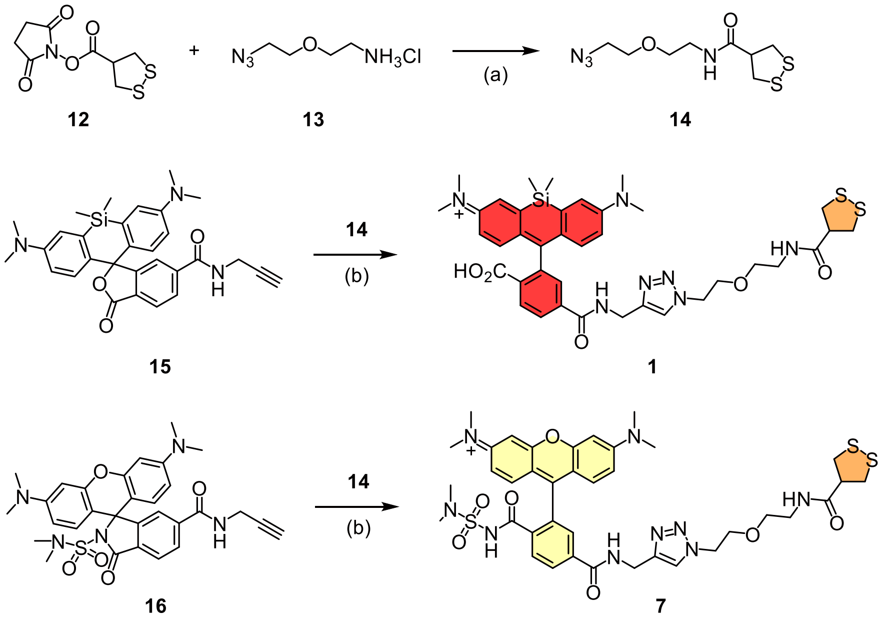

Scheme S1. (a) DIPEA, CH_2Cl_2, rt, overnight, 97%, (b) CuI, THPTA, THF, 30 h, 44% for **1** and 56% for **7**.

Compound 12 was prepared following the procedure described in ref S2.

Compound 13 was prepared following the procedure described in ref S3.

Compound 14. To a solution of **12** (150 mg, 607 μmol) in dry CH_2Cl_2 (50 mL) was added **13** (152 mg, 910 μmol) and DIPEA (429 μL, 2.43 mmol). The reaction mixture was stirred at rt overnight then it was quenched with 1 M HCl. The crude product was extracted with CH_2Cl_2 (x3), washed with brine, dried over Na_2SO_4 and concentrated *in vacuo*. The crude product was subjected to purification by flash chromatography (Scorpius silica 12 g, CH_2Cl_2/MeOH 95:5) to afford the desired **14** (155 mg, 97%) as a colorless solid. R_f (CH_2Cl_2/MeOH 95:5): 0.52; Mp: 54-55 °C; IR (neat): 3296 (br, NH), 2101 (s, N_3), 1637 (s, C=O), 1551 (s, N-C=O), 1297 (m), 1250 (s), 1112 (s, C-O-C), 1079 (s, C-O-C), 1045 (m, C-O), 683 (s, C-S); ^1H NMR (400 MHz, $CDCl_3$): 6.17 (s, 1H), 3.70 (t, $^3J_{H-H}$ = 4.0 Hz, 2H), 3.58 (t, $^3J_{H-H}$ = 5.0 Hz, 2H), 3.51 (q, $^3J_{H-H}$ = 5.0 Hz, 2H), 3.40 – 3.37 (m, 4H), 3.36 (s, 2H), 3.16 (p, $^3J_{H-H}$ = 7.0 Hz, 1H); ^{13}C NMR (126 MHz, $CDCl_3$): 171.8 (C), 70.4 (CH_2), 69.8 (CH_2), 52.6 (CH), 50.8 (CH_2), 42.8 ($2CH_2$), 39.6 (CH_2); LRMS (ESI): 263 ($C_8H_{15}N_4O_2S_2$, $[M+H]^+$).

General procedure A. To a mixture of alkyne (1 equiv, final concentration between 2-5 mM) and azide (1-2 equiv.) in dry THF under N_2 atmosphere was added a pre-mixed solution of CuI (2 equiv.) and TBTA (0.5 equiv.) or THPTA (1 equiv.) in dry THF. The reaction mixture was stirred at rt and monitored by LC-MS. After completion of reaction (2 – 30 h), the crude mixture was filtered to remove the excess of CuI and then the filtrate was concentrated *in vacuo*. The crude solid was subjected to purification by RP flash chromatography to afford the desired triazole product.

Compound 1. Following the general procedure A, **15** (7.0 mg, 14 μmol) and compound **14** (7.2 mg, 28 μmol) were suspended in dry THF (5.0 mL) with CuI (5.2 mg, 28 μmol) and THPTA (6.0 mg, 14 μmol). The reaction mixture was stirred at rt for 30 h (completion evidenced by LC-MS). RP-chromatography (Scorpius C18 33g, H_2O + 0.1% TFA / CH_3CN + 0.1% TFA, 1:1) afforded the corresponding compound **1** (4.7 mg, 44%) as a blue solid. Mp: 120-121 °C; IR (neat): 2923 (br, NH), 1658 (s, C=O), 1575 (s, N-C=O), 1353 (s, C-N), 1314 (s, C-N), 1124 (s, $Si(CH_3)_2$), 922 (w, CH), 835 (m, Si-C), 719 (w); ^1H NMR (500 MHz, DMSO-d_6): 9.30 (t, $^3J_{H-H}$ = 5.7 Hz, 1H), 8.16 – 8.08 (m, 2H), 8.04 (d, $^3J_{H-H}$ = 8.0 Hz, 1H), 7.94 (s, 1H), 7.72 (s, 1H), 7.04 (s, 2H), 6.76 – 6.51 (m, 4H), 4.47–4.45

(m, 4H), 3.76 (t, $^3J_{H-H}$ = 5.3 Hz, 2H), 3.40 (t, $^3J_{H-H}$ = 5.7 Hz, 2H), 3.36 – 3.29 (m, 2H), 3.18 (q, $^3J_{H-H}$ = 5.7 Hz, 2H), 3.15 – 3.09 (m, 3H), 2.93 (s, 12H), 0.64 (s, 3H), 0.52 (s, 3H); ^{13}C NMR (126 MHz, DMSO-d_6): 170.5 (C), 169.2 (C), 164.7 (C), 158.2 (C), 154.7 (C), 149.2 (2C), 144.3 (C), 139.1 (C), 136.0 (2C), 130.5 (C), 128.3 (2CH), 127.7 (2CH), 125.4 (C), 123.5 (CH), 123.0 (CH), 116.5 (2CH), 113.9 (2CH), 91.3 (C), 68.6 (CH_2), 68.4 (CH_2), 51.3 (CH), 49.2 (CH_2), 42.1 (2CH_2), 40.0 (4CH_3), 38.7 (CH_2), 35.0 (CH_2), 0.0 (CH_3), -1.4 (CH_3); HRMS (ESI, +ve) calcd for $C_{38}H_{45}N_7O_5S_2Si$ [M+Na]$^+$: 794.2585, found: 794.2574.

Compound 6 was prepared following the procedure described in ref S2.

Compound 7. Following the general procedure A, **16** (6.3 mg, 11 μmol) and **14** (3.7 mg, 14 μmol) were suspended in dry THF (5.0 mL) with CuI (4.2 mg, 22 μmol) and THPTA (4.8 mg, 11 μmol). The reaction mixture was stirred at rt for 30 h (completion monitored by LC-MS). RP-chromatography (Scorpius C18 33g, H_2O + 0.1% TFA / CH_3CN + 0.1% TFA, 1:1) afforded the corresponding compound **7** (5.0 mg, 54%) as a purple solid. Mp: 162-163 °C; IR (neat): 3440 (br, NH), 2250 (w), 1660 (w, C=O), 1050 (s, C-O-C), 1023 (s, C-O-C), 1005 (s), 821 (m), 758 (m), 618 (m); ^{1}H NMR (400 MHz, DMSO-d_6): 9.21 (t, $^3J_{H-H}$ = 5.8 Hz, 1H), 8.10–8.09 (m, 2H), 7.99 (d, $^3J_{H-H}$ = 8.0 Hz, 1H), 7.89 (s, 1H), 7.48 (s, 1H), 6.52 (d, $^3J_{H-H}$ = 8.5 Hz, 2H), 6.41–6.38 (m, 4H), 4.44 (t, $^3J_{H-H}$ = 5.2 Hz, 2H), 4.40 (d, $^3J_{H-H}$ = 5.8 Hz, 2H), 3.74 (t, $^3J_{H-H}$ = 5.2 Hz, 2H), 3.38 (t, $^3J_{H-H}$ = 5.8 Hz, 2H), 3.37 – 3.28 (m, 2H), 3.20 – 3.05 (m, 5H), 2.92 (s, 12H), 2.62 (s, 6H); ^{13}C NMR (126 MHz, DMSO-d_6): 170.6 (C), 165.8 (C), 164.5 (C), 153.8 (C), 152.5 (2C), 151.1 (2C), 144.4 (C), 139.8 (C), 131.3 (C), 128.7 (2CH), 128.4 (CH), 123.7 (CH), 123.5 (CH), 123.1 (CH), 108.3 (2CH), 106.4 (2C), 98.2 (2CH), 69.0 (C), 68.6 (CH_2), 68.4 (CH_2), 51.3 (CH), 49.2 (CH_2), 42.1 (2CH_2), 40.0 (4CH_3), 38.7 (CH_2), 37.5 (2CH_3), 34.9 (CH_2); HRMS (ESI, +ve) calcd for $C_{38}H_{45}N_9O_7S_3$ [M+Na]$^+$: 858.2497, found: 858.2463.

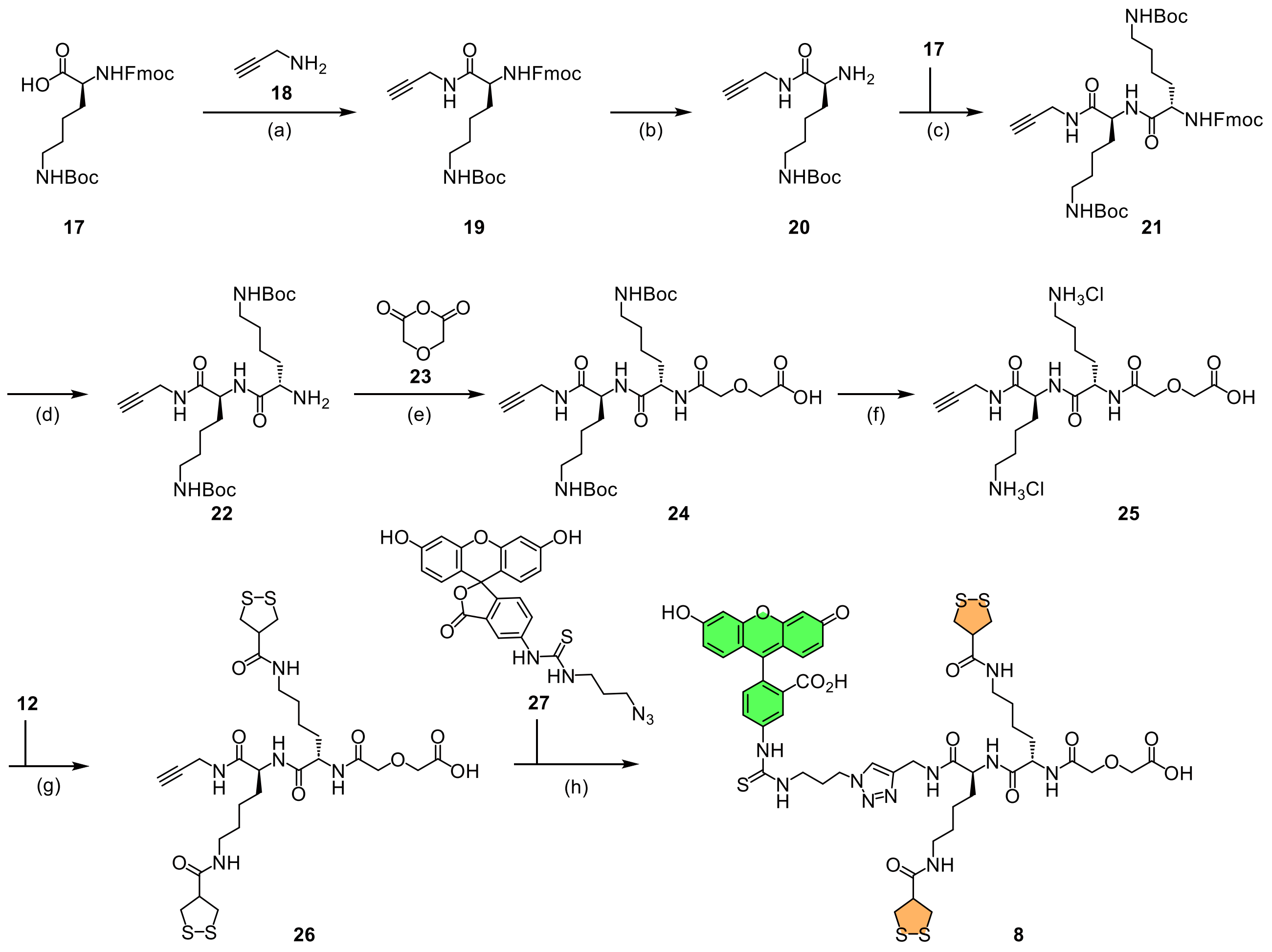

Scheme S2. (a) HATU, DIPEA, DMF, rt, 1 h, 83%; (b) dimethylamine, THF, rt, 30 min, 88%; (c) EDC HCl, CH_2Cl_2, rt, 40 min; (d) dimethylamine, THF, rt, 40 min, 78% over 2 steps; (e) THF, rt, 1 h, 78%; (f) HCl, dioxane, 0 to rt, 1 h; (g) DIPEA, DMF, rt, 10 min, 66% over 2 steps; (h) CuI, TBTA, THF, rt, 2 h, 20%.

Compounds 19 and **20** were prepared following the procedure described in ref S4.

Compound 21. To a solution of **20** (698 mg, 1.5 mmol) and EDC HCl (343 mg, 1.8 mmol) in anhydrous CH_2Cl_2 (20 mL) was added a solution of **17** (422 mg, 1.5 mmol) in anhydrous CH_2Cl_2 (3 mL). The reaction mixture was stirred for 40 min at rt until completion (monitored by LC-MS). The reaction mixture was washed with water (x3) to remove the urea by-product. The aqueous phase was extracted with CH_2Cl_2 (×3). The combined organic layers were dried over Na_2SO_4, filtered, and

concentrated *in vacuo*. The crude product was directly engaged in the next step without any further purification.

Compound 22. Compound **21** (1.9 g, 2.6 mmol) was dissolved in THF containing dimethylamine (30 mL of 2 M, 60 mmol) and the reaction mixture was stirred at rt for 40 min. Once the starting material was completely consumed, the crude mixture was concentrated *in vacuo*. The crude product was solubilized in small amount of CH_2Cl_2 and triturated several times with pentane to afford compound **22** (1.31 g, quant; 78% from **20**) as a pale yellow oil. R_f (CH_2Cl_2/MeOH 8:2): 0.44; $[\alpha]_D^{20}$ -14 (*c* 1.0, MeOH), IR (neat): 3299 (m, H-C≡C), 2932 (m, NH), 2485 (m, C≡C), 1678 (s, C=O), 1365 (s, C-N), 1166 (s, C-O), 988 (s), 779 (w), 779 (w); ^{1}H NMR (300 MHz, CD_3OD): 4.33 – 4.29 (m, 1H), 4.01 (dd, $^2J_{H\text{-}H}$ = 17.5, $^4J_{H\text{-}H}$ = 2.6 Hz, 1H), 3.92 (dd, $^2J_{H\text{-}H}$ = 17.5, $^4J_{H\text{-}H}$ = 2.6 Hz, 1H), 3.37 – 3.33 (t, $^3J_{H\text{-}H}$ = 6.5 Hz, 1H), 3.06 – 3.00 (m, 4H), 2.61 – 2.59 (t, $^4J_{H\text{-}H}$ = 2.6 Hz, 1H), 1.34 – 1.84 (m, 30H); ^{13}C NMR (75 MHz, CD_3OD): 177.8 (C), 174.0 (C), 158.5 (2C), 80.4 (C), 79.9 (2C), 72.4 (CH), 55.9 (CH), 54.3 (CH), 41.1 (2CH_2), 36.0 (CH_2), 33.0 (CH_2), 30.7 (CH_2), 30.5 (CH_2), 29.5 (CH_2), 28.8 (6CH_3), 24.1 (CH_2), 23.8 (CH_2); LRMS (ESI): 513 ($C_{25}H_{46}N_5O_6$, $[M+H]^+$).

Compound 24. To a solution of **22** (500 mg, 1 mmol) in THF (14 mL), was added **23** (113 mg, 1 mmol) and the reaction mixture was stirred for 1 h at rt. After the completion of the reaction (monitored by LC-MS), the crude mixture was concentrated *in vacuo*. The crude product was dissolved in a minimum volume of CH_2Cl_2, and pentane was added until the solution turned visibly cloudy. The solution was filtered over celite. The filtrate was recovered and concentrated *in vacuo* and engaged in the same process. After 3 filtrations, the filtrate was discarded and the celite was washed with CH_2Cl_2 (x3) to recover the product. The combined organic phases were concentrated *in vacuo* and dried to afford **24** (1.3 g, 78%) as a yellow oil. R_f (CH_2Cl_2/MeOH 8:2): 0.18; $[\alpha]_D^{20}$ -6.9 (*c* 0.50, MeOH); IR (neat): 3312 (m, H-C≡C), 2933 (m, NH), 2416 (m, C≡C), 1647 (s, C=O), 1524 (CH_2-C=O, m), 1444 (s, N-C=O), 1168 (s, C-O-C), 991 (w), 865(w), 663 (w); ^{1}H NMR (500 MHz, CD_3OD): 4.41–4.36 (m, 1H), 4.32–4.27 (m, 1H), 4.23 (s, 2H), 4.12 (s, 2H), 3.99 (dd, $^2J_{H\text{-}H}$ = 17.5,

$^4J_{H-H}$ = 2.6 Hz, 1H), 3.92 (dd, $^2J_{H-H}$ = 17.5, $^4J_{H-H}$ = 2.6 Hz, 1H), 3.06 – 3.00 (m, 4H), 2.59 (t, $^4J_{H-H}$ = 2.6 Hz, 1H), 1.90 – 1.41 (m, 30H); ^{13}C NMR (126 MHz, CD_3OD): 174.0 (C), 173.9 (C), 173.8 (C), 172.3 (C), 158.5 (2C), 80.4 (C), 79.9 (2C), 72.3 (CH), 71.6 (CH_2), 69.5 (CH_2), 54.5 (2C), 41.1 (2CH_2), 32.7 (2CH_2), 30.5 (2CH_2), 29.5 (CH_2), 28.8 (6CH_3), 24.1 (CH_2), 24.0 (CH_2); LRMS (ESI): 629 ($C_{29}H_{50}N_5O_{10}$, $[M+H]^+$).

Compound 25. To **24** (451 mg, 0.7 mmol) was added in one portion a solution of HCl in dioxane (3.6 mL of 4 M, 14 mmol) at 0 °C. A white precipitate formed in a few seconds after the addition. After stirring for 1 h at rt (completion monitored by LC-MS), the reaction mixture was concentrated *in vacuo*. The crude product was dissolved in water and then lyophilized to afford compound **25** as a colorless solid. The formed salt was engaged in the next step without any further purification.

Compound 26. To a solution of **25** (110 mg, 0.20 mmol) in DMF (7.6 mL), was added DIPEA (190 µL, 1.1 mmol) to adjust pH to 8-10. Then, **12** (140 mg, 0.60 mmol) was added to the solution. After 10 min (completion was evidenced by LC-MS), diethyl ether (10 mL) was added to the reaction mixture and precipitation was observed. The mixture was sonicated for 1 min and centrifuged. After centrifugation, a yellow sticky deposit was observed at the bottom of the vial. The supernatant was discarded. The residue was then suspended in HCl 1 M (10 mL), sonicated, centrifuged, and the supernatant was discarded (x3). The same procedure was performed with H_2O (x3) and CH_3CN (x3) to give **26** (60 mg, 66% from **24**) as a colorless solid. Mp: > 241 °C decomposed; IR (neat): 3288 (m, H-C≡C), 3074 (w, OH), 2928 (m, C≡C), 1639 (s, C=O), 1537 (s, CH_2-C=O), 1455 (m), 1235 (s, N-C=O), 1131 (s, C-O-C), 972 (m); 870 (w); 666 (s); ^{1}H NMR (400 MHz, DMSO-d_6): 12.82 (bs, 1H), 8.35 (t, $^3J_{H-H}$ = 5.6 Hz, 1H), 8.09–8.06 (m, 3H), 7.80 (d, $^3J_{H-H}$ = 8.1 Hz, 1H), 4.39 – 4.28 (m, 1H), 4.25 – 4.15 (m, 1H), 4.13 (s, 2H), 4.00 (s, 2H), 3.86–3.83 (m, 2H), 3.42 – 3.36 (m, 4H), 3.22 – 3.10 (m, 6H), 3.10 (t, $^4J_{H-H}$ = 2.6 Hz, 3H), 3.08 – 2.99 (m, 4H), 1.72 – 1.46 (m, 4H), 1.43 – 1.32 (m, 4H), 1.33 – 1.17 (m, 4H); ^{13}C NMR (101 MHz, DMSO-d_6): 171.4 (C), 171.3 (C), 171.2 (C), 170.2 (C),

168.7 (2C), 80.9 (C), 73.1 (CH), 69.9 (CH_2), 67.9 (CH_2), 52.3 (CH), 51.9 (CH), 51.5 (2CH), 42.1 (4CH_2), 38.7 (overlap with DMSO, 2CH_2), 31.9 (CH_2), 31.6 (CH_2), 28.6 (2CH_2), 27.9 (CH_2), 22.7 (CH_2), 22.5 (CH_2); LRMS (ESI): 692 ($C_{27}H_{42}N_5O_8S_4$, $[M+H]^+$).

Compound 27 was prepared following the procedure described in ref S5.

Compound 8. Following the general procedure A, **26** (10 mg, 15 μmol) and **27** (9.2 mg, 19 μmol) were suspended in dry THF (5.0 mL) with CuI (5.5 mg, 29 μmol) and TBTA (3.8 mg, 7.0 μmol). The reaction mixture was stirred at rt for 2 h (completion monitored by LC-MS). RP-chromatography (Scorpius C18 33 g, H_2O + 0.1% TFA / CH_3CN + 0.1% TFA, 1:1) afforded **8** (3.5 mg, 20%) as a neon green powder. Mp: > 240 °C decomposed; IR (neat): 3268 (m, NH), 3074 (m, OH), 2935 (m, NH), 2627 (m), 2286 (m), 2100 (s), 1634 (s, C=O), 1586 (s, N-C=O), 1538 (s, C=N), 1385 (s, C-N), 1270 (s, C-O-C), 1117 (s, CH_2-O-CH_2), 995 (m), 915 (m), 841 (m), 763 (m), 795 (m), 716 (m), 671 (m), 597 (m, C-H), 517 (w, S-S); ^1H NMR (400 MHz, DMSO-d_6): 10.11 (br s, 3H), 8.39 (t, $^3J_{H-H}$ = 5.6 Hz, 1H), 8.30 (s, 1H), 8.24 (s, 1H), 8.09 – 8.06 (m, 3H), 7.94 (s, 1H), 7.79 (d, $^3J_{H-H}$ = 8.1 Hz, 1H), 7.78 – 7.73 (m, 2H), 7.18 (d, $^3J_{H-H}$ = 8.3 Hz, 1H), 6.67 (d, $^4J_{H-H}$ = 2.2 Hz, 2H), 6.60 (d, $^3J_{H-H}$ = 8.7 Hz, 2H), 6.56 (dd, $^3J_{H-H}$ = 8.7, $^4J_{H-H}$ = 2.2 Hz, 2H), 4.42 (t, $^3J_{H-H}$ = 6.0 Hz, 2H), 4.36 – 4.29 (m, 3H), 4.27 – 4.17 (m, 2H), 4.13 (s, 2H), 4.00 (s, 2H), 3.52 (q, $^3J_{H-H}$ = 6.3 Hz, 2H), 3.39 – 3.32 (m, 5H), 3.20 – 3.07 (m, 7H), 3.03 (q, $^3J_{H-H}$ = 6.6 Hz, 4H), 2.13 (p, $^3J_{H-H}$ = 7.1 Hz, 2H), 1.71 – 1.47 (m, 4H), 1.43 – 1.34 (m, 4H), 1.31 – 1.17 (m, 4H); ^{13}C NMR (126 MHz, DMSO-d_6): 180.1 (C), 171.5 (C), 171.3 (C), 170.2 (2C), 168.5 (3C), 159.5 (2C), 158.2 (C), 151.9 (2C), 145.8 (C), 141.1 (C), 129.0 (2CH), 126.5 (CH), 124.0 (C), 122.7 (2CH), 115.9 (CH), 112.6 (2CH), 109.7 (2C), 102.2 (2CH), 83.0 (C), 70.6 (CH_2), 67.8 (CH_2), 52.5 (CH), 51.9 (CH), 51.5 (2CH), 47.2 (CH_2), 42.1 (4CH_2), 41.1 (C), 40.3 (2CH_2), 38.7 (CH_2), 34.3 (CH_2), 31.8 (CH_2), 29.3 (CH_2), 28.6 (CH_2), 28.5 (CH_2), 22.8 (CH_2), 22.6 (CH_2); HRMS (ESI, -ve) calcd for $C_{51}H_{60}N_{10}O_{13}S_5$ $[M-H]^-$: 1179.2871, found: 1179.2888.

Compound 9 was synthesized by a procedure that will be reported elsewhere.

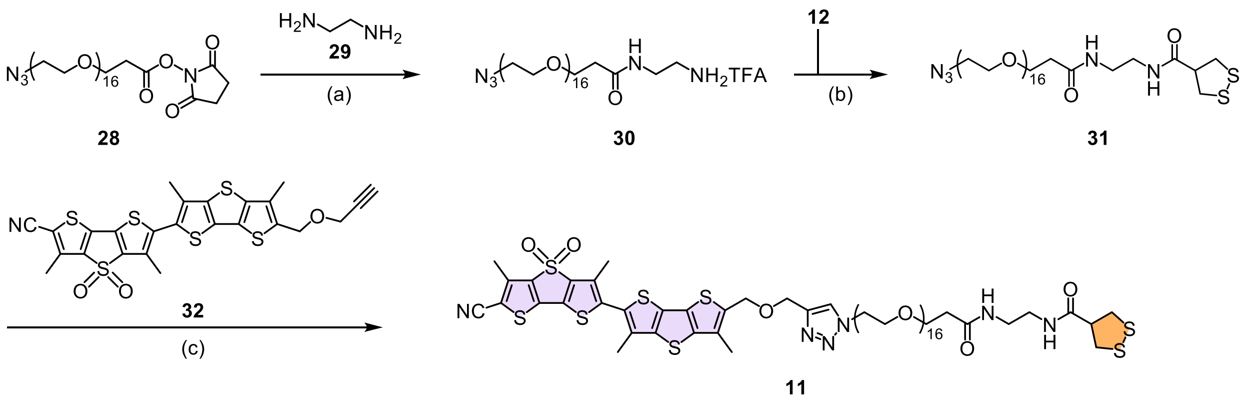

Scheme S3. (a) DMF, rt, 5 min, 80%, (b) DIPEA, DMF, rt, 5 min, 52%, (c) TBTA, $CuSO_4 \cdot 5H_2O$, sodium ascorbate, CH_2Cl_2/H_2O 2:1, rt, 2 h, 60%.

Compound 30. To a stirred solution of **29** (33 mg, 550 µmol) in dry DMF (3.5 mL) was added **28** (100 mg, 109 µmol) under N_2 atmosphere. After 5 min at rt, the solvent was removed *in vacuo*. The crude product was subjected to purification by RP-chromatography (Scorpius C18 20 g, CH_3CN + 0.1% TFA / H_2O + 0.1% TFA 0:1 to 3:7) to afford **30** (85 mg, 80%) as a colorless oil. IR (neat): 3074 (br, w, N-H), 2868 (s, C-H), 2104 (s, N_3), 1674 (s, C=O amide), 1537 (w, N-H), 1454 (w, CH_2), 1200 (m, C-N), 1093 (br, s, C-O-C), 948 (m), 830 (m), 799 (m), 719 (m), 557 (w), 518 (w); ^{1}H NMR (400 MHz, CD_3OD): 3.76 (t, $^3J_{H\text{-}H}$ = 5.7 Hz, 2H), 3.70 – 3.62 (m, 62H), 3.52 (t, $^3J_{H\text{-}H}$ = 6.2 Hz, 2H), 3.38 (t, $^3J_{H\text{-}H}$ = 4.9 Hz, 2H), 3.16 (t, $^3J_{H\text{-}H}$ = 6.2 Hz, 2H), 2.48 (t, $^3J_{H\text{-}H}$ = 5.7 Hz, 2H); ^{13}C NMR (101 MHz, CD_3OD): 175.3 (C=O), 71.6 – 71.1 (31CH_2), 68.2 (CH_2), 51.8 (CH_2), 40.8 (CH_2), 37.9 (CH_2), 37.7 (CH_2); LRMS (ESI): 862 ($C_{37}H_{76}N_5O_{17}$, $[M\text{-}TFA+H]^+$).

Compound 31. To a stirred solution of **30** (42 mg, 43 µmol) and **12** (16 mg, 64 µmol) in dry DMF (1.5 mL) was added DIPEA (15 µL, 85 µmol) dropwise under N_2 atmosphere and the pH of the reaction was kept at 8. The reaction mixture was stirred at room temperature for 5 min and the solvent was removed *in vacuo*. The residue was subjected to purification by RP-chromatography (Scorpius C18 20 g, CH_3CN / H_2O 2:8 to 4:6) to afford **31** (22 mg, 52%) as a colorless oil. IR (neat): 3317 (br, w, N-H), 3072 (br, w, N-H), 2867 (s, C-H), 2102 (s, N_3), 1667 (s, C=O amide), 1540 (m, N-H), 1453 (m, CH_2), 1201 (w, C-N), 1096 (br, s, PEG), 948 (m), 846 (m), 718 (w), 647 (w), 558 (w, S-S); ^{1}H

NMR (400 MHz, DMSO-d_6): 8.13 (s, 1H), 7.88 (s, 1H), 3.62 – 3.46 (m, 64H), 3.40 – 3.34 (m, 4H), 3.23 – 3.14 (m, 2H), 3.13 – 3.09 (m, 5H), 2.30 (t, $^3J_{H\text{-}H}$ = 6.5 Hz, 2H); ^{13}C NMR (101 MHz, DMSO-d_6): 170.6 (C=O), 170.2 (C=O), 69.8 – 69.2 (31CH$_2$), 66.7 (CH$_2$), 51.5 (CH), 50.0 (CH$_2$), 42.0 (2CH$_2$), 38.7 (CH$_2$), 38.1 (CH$_2$), 36.2 (CH$_2$); LRMS (ESI): 994 ($C_{41}H_{80}N_5O_{18}S_2$, [M+H]$^+$).

Compound 32 was synthesized and purified according to the procedures reported in ref. S6 and S7.

Compound 11. To a solution of **31** (20.0 mg, 201 μmol), TBTA (9.3 mg, 18 μmol), **32** (10 mg, 18 μmol) in CH_2Cl_2 (400 μL), a solution of $CuSO_4 \cdot 5H_2O$ (22 mg, 87 μmol) and sodium ascorbate (34.6 mg, 175 μmol) in H_2O (200 μL) were added dropwise under N_2 atmosphere. The reaction was stirred at room temperature for 2 h. The mixture was extracted with CH_2Cl_2/brine, and the organic phase was dried with Na_2SO_4, and concentrated under reduced pressure. The residue was subjected to purification by RP chromatography (Scorpius C18 20 g, CH_3CN+ 0.1% TFA / H_2O 0:1 to 1:0) to afford **11** (16.4 mg, 60%) as an orange solid. IR (neat): 3318 (br, w, N-H), 3134 (br, w, N-H), 2868 (s, C-H), 2212 (m, C≡N), 1666 (s, C=O amide), 1540 (m, N-H), 1451 (m, CH$_2$), 1417 (m), 1350 (w), 1314 (m, SO$_2$), 1258 (w), 1198 (w), 1142 (s, SO$_2$), 1100 (br, s, PEG), 950 (m), 804 (m), 667 (w), 598 (w), 580 (w), 558 (m, S-S); Mp: >200 °C decomposed; ^{1}H NMR (500 MHz, DMSO-d_6): 8.11 (s, 2H), 7.87 (s, 1H), 4.78 (s, 2H), 4.63 (s, 2H), 4.53 (t, $^3J_{H\text{-}H}$ = 5.3 Hz, 2H), 3.81 (t, $^3J_{H\text{-}H}$ = 5.3 Hz, 2H), 3.58 (t, $^3J_{H\text{-}H}$ = 6.5 Hz, 2H), 3.51 – 3.45 (m, 60H), 3.39 – 3.35 (m, 2H), 3.22 – 3.14 (m, 2H), 3.13 – 3.08 (m, 5H), 2.48 (s, 3H, overlapped with DMSO peak), 2.36 (s, 3H), 2.34 (s, 3H), 2.32 (s, 3H), 2.30 (t, $^3J_{H\text{-}H}$ = 6.5 Hz, 2H); ^{13}C NMR (126 MHz, DMSO-d_6): 170.6 (C=O), 170.3 (C=O), 143.9 (C), 143.5 (C), 142.2 (C), 141.7 (C), 140.8 (C), 139.9 (C), 138.1 (C), 136.9 (C), 133.3 (C), 131.5 (C), 130.5 (C), 130.2 (C), 128.5 (C), 127.9 (C), 125.9 (C), 124.5 (C), 113.1 (C), 109.9 (C), 69.8 – 69.5 (30CH$_2$), 68.7 (CH$_2$), 66.8 (CH$_2$), 64.5 (CH$_2$), 62.7 (CH$_2$), 54.9 (CH$_2$), 51.5 (CH), 49.4 (CH$_2$), 42.0 (2CH$_2$), 38.7 (CH$_2$), 38.1 (CH$_2$), 36.2 (CH$_2$), 13.8 (CH$_3$), 12.6 (CH$_3$), 12.5 (CH$_3$), 12.0 (CH$_3$); HRMS (ESI+) calcd. for $C_{66}H_{96}N_6O_{21}S_8$ [M+Na]$^+$: 1587.4287, found: 1587.4347.

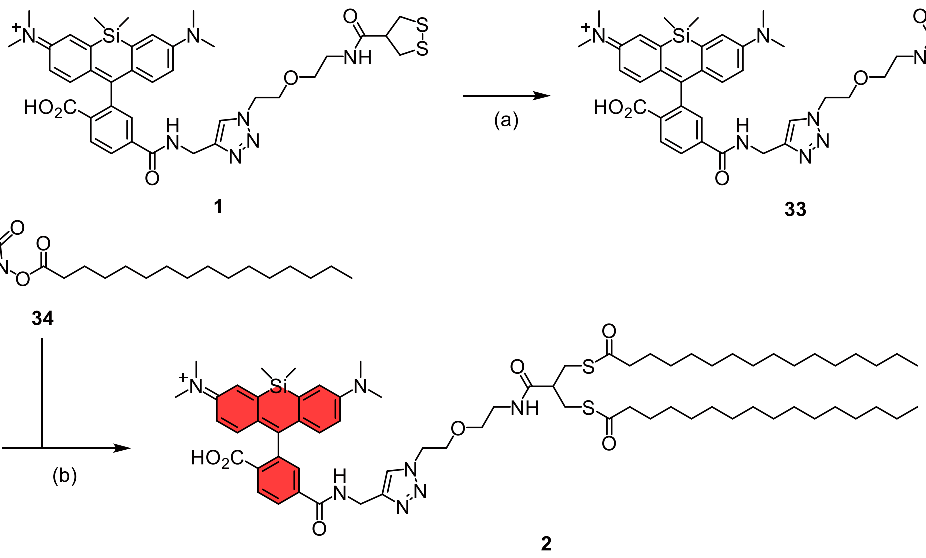

Scheme S4. (a) THPP, DMF, 25 °C, 1 h, (b) **34**, DIPEA, DMF, rt, 4 h.

Compound 33. To a solution of **1** (2.0 mg, 2.6 μmol) in anhydrous DMF (40 μL) was added in one portion tris(hydroxypropyl)phosphine (2.0 μL, 11 μmol) and the reaction mixture was gently shaken (800 rpm) at 25 °C for 1 h. Once **1** was consumed (monitored by LC-MS), the reaction mixture was engaged in the next step without further purification.

Compound 2. To the previous crude mixture containing compound **33** in DMF was added a solution of **34** (9.2 mg, 26 μmol) in DIPEA (9.2 μL, 52 μmol). The reaction mixture was shaken at 25 °C for 2 h (monitored by LC-MS). An extra solution of **34** (9.2 mg, 26 μmol) in DIPEA (9.2 μL, 52 μmol) was added and the reaction mixture was shaken for an additional 2 h at 25 °C. Once most of the starting material was consumed (confirmed by LC-MS), the reaction mixture was quenched in 1 M HCl (2 mL), and extracted with EtOAc (x3). The combined organic layers were dried over Na_2SO_4, filtered and concentrated *in vacuo* and subjected to purification by RP-chromatography (Scorpius C18 33g, CH_3CN + 0.1% TFA isocratic) to afford compound **2** (0.8 mg, 27%) as a blue powder. ^{1}H NMR (400 MHz, DMSO-d_6): 9.29 (t, $^3J_{H\text{-}H}$ = 5.6 Hz, 1H), 8.12 (dd, $^3J_{H\text{-}H}$ = 8.1, $^4J_{H\text{-}H}$ =1.4 Hz, 1H), 8.06 (t, $^3J_{H\text{-}H}$ = 5.6 Hz, 1H), 8.01 (d, $^3J_{H\text{-}H}$ = 8.1 Hz, 1H), 7.93 (s, 1H), 7.72 (s, 1H), 7.01 (d, $^3J_{H\text{-}H}$ = 2.5 Hz, 2H), 6.67 – 6.56 (m, 4H), 4.49 – 4.42 (m, 4H), 3.75 (t, $^3J_{H\text{-}H}$ = 5.3 Hz, 2H), 3.38 – 3.35 (m, 2H), 3.13 (q, $^3J_{H\text{-}H}$ = 5.8 Hz, 2H), 3.00 (dd, $^3J_{H\text{-}H}$ = 13.4, $^2J_{H\text{-}H}$ = 6.2 Hz, 2H), 2.92 –

2.87 (m, 14H), 1.51 (t, $^3J_{H-H}$ = 7.2 Hz, 4H), 1.22 (s, 24H), 1.20 (s, 24H), 0.88 – 0.80 (m, 6H), 0.63 (s, 3H), 0.51 (s, 3H); ^{13}C NMR (126 MHz, DMSO):198.2 (2C), 171.2 (C), 169.1 (C), 166.6 (2C), 164.7 (C), 154.7 (C), 149.2 (C), 144.5 (2C), 140.0 (C), 135.9 (2C), 130.4 (C), 128.3 (CH), 127.7 (2CH), 125.3 (CH), 123.5 (CH), 123.0 (CH), 116.4 (2CH), 113.7 (2CH), 95.3 (C), 68.7 (CH_2), 68.5 (CH_2), 49.2 (CH_2), 45.0 (2CH_2), 43.3 (CH_2), 38.4 (CH_2), 35.0 (palm), 31.3 (palm), 29.6 (palm), 29.0 (palm), 29.0 (palm), 28.8 (palm), 28.7 (palm), 28.7 (palm), 28.2 (palm), 25.0 (2CH_2), 22.1 (2CH_2), 13.9 (2CH_3), 0.1 (CH_3), -1.4 (CH_3); HRMS (ESI+) calcd. for $C_{70}H_{108}N_7O_7S_2Si$ $[M]^+$: 1250.7521, found: 1250.7561.

3. Spectroscopic Properties

Stock solutions of **1** and **7** (1 mM in DMSO) were diluted to give 1 µM solutions in PBS. In parallel, solutions of fluorescent compounds (10 µL, 100 µM in PBS) were mixed with DTT (10 µL, 2 mM in PBS) for 30 min at 25 °C before diluting to 1 µM in PBS. The fluorescence emission spectra were then recorded from 650 nm to 800 nm for **1** (excitation at 640 nm, slits 3) and from 565 nm to 800 nm for **7** (excitation at 550 nm, slits 2). The quenching factor (QF) for **1** was calculated by first dividing the emission intensity at 665 nm by the absorbance at 650 nm for the oxidized AspA (disulfide, closed) and the reduced AspA (thiols, open). The ratio of these values (open/closed) provided the QF = 5.3 for **1**. The same calculation was applied to **7**, except that the value of emission intensity was at 575 nm and the absorbance at 556 nm, to give QF = 3.7.

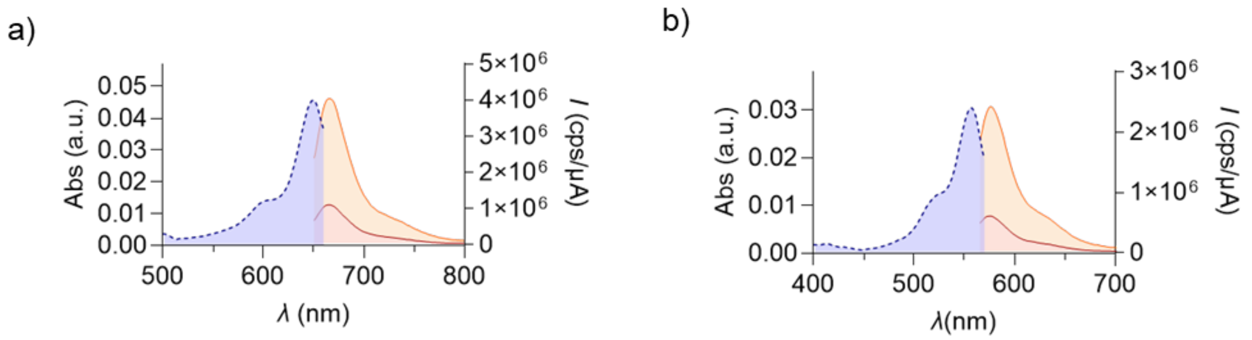

Figure S2. Absorption (dashed) and emission (solid) spectra of oxidized (red solid) and reduced (orange solid) probes a) **1** and b) **7** in PBS upon excitation at 640 nm and 550 nm, respectively. l = 0.3 cm.

4. Cell Culture

Human cervical cancer-derived HeLa Kyoto, human breast cancer-derived MCF-7, human retinal pigment epithelial-1 (RPE-1), Madin-Darby canine kidney (MDCK) and human epidermoid carcinoma (A-431) cells were cultured in complete FDMEM (GlutaMAX, 4.5 g/L D-glucose) medium, which contains 10% fetal calf serum (FCS) and 1% Penicillin/Streptomycin (PS). HeLa CCL-2 (ECACC General Collection) were cultivated in DMEM supplemented with 10% FBS and 1% PS. The cells were grown under 5% CO_2 humidified atmosphere at 37 °C on a 75 cm^3 tissue culture flask (TPD Corporation). Cells were harvested by treatment with 3 mL of phenol-red free TrypLE Express, followed by the addition of 10 mL of complete FDMEM (GlutaMAX, 4.5 g/L D-glucose) medium at 37 °C. The cells were spun down at 1500 g for 3 min, re-suspended in complete FDMEM (GlutaMAX, 4.5 g/L D-glucose) medium, and plated according to the concentration needed. For uptake or inhibition experiments, the cells were seeded in a μ-Plate 96-well Black ibiTreat sterile at 12 000 cells/well (HK, RPE-1, A-431) or 18 000 cells/well (MCF-7, MDCK) in complete FDMEM and left incubating under 5% CO_2 humidified atmosphere at 37 °C overnight.

5. Golgi Tracking

5.1. General Procedure

Cells were prepared in a 96 well plate as described in section 4, then medium was removed, and cells were washed with PBS (3 × 3 mL/well) followed by fresh FDMEM serum-free medium (4 × 100 μL/well) using a plate washer (Biotek EL406®), and kept in a 100 μL of the latter medium. The solution of **1**, **2**, **6-9** (2-10 mM, DMSO) was diluted in FDMEM to give a solution at 3x final concentration, of which 50 μL was added to the well resulting in a final volume of 150 μL per well. The cells were incubated under 5% CO_2 humidified atmosphere at 37 °C for the indicated time (5 – 120 min). Afterward, the cells were washed with PBS (3 x 3 mL/well) and the medium was exchanged with FDMEM keeping a final volume of 100 μL/well, and a solution of Hoechst 33342 (100 μg/mL) in PBS (50 μL/well) was added. After 10 min of incubation under 5% CO_2 humidified atmosphere at 37 °C, cells were washed with PBS (3 × 3 mL/well) and kept in FDMEM (100 μL/well) for live cell imaging. For fixed cell imaging, the cells were treated with a solution of PFA 3% (70 μL/well) for 15 min at rt. The excess of PFA was removed by washing with PBS (9 x 3 mL/well). The distribution of fluorescent signals was captured on a IXM-C automated microscope with two channels, blue for Hoechst 33342 (377/50 nm excitation filter; 477/60 nm emission filter), green (475/34 nm excitation filter; 536/40 nm emission filter, for **6**, **8**, and **9**), red (531/40 nm excitation filter; 593/40 nm emission filter, for **7**) or far red (620/50 nm; emission filter: 690/50 nm, for **1** or **2**). The rest of the parameters were adjusted according to the nature of the experiment. Duplicates were performed for each condition.

5.2. Data Analysis

Resulting images were automatically analyzed and quantified using the MetaXpress software. For quantification of the fluorescent signal in the whole cell, the data analysis method from reference S1 was applied.

5.3. FITC-AspA

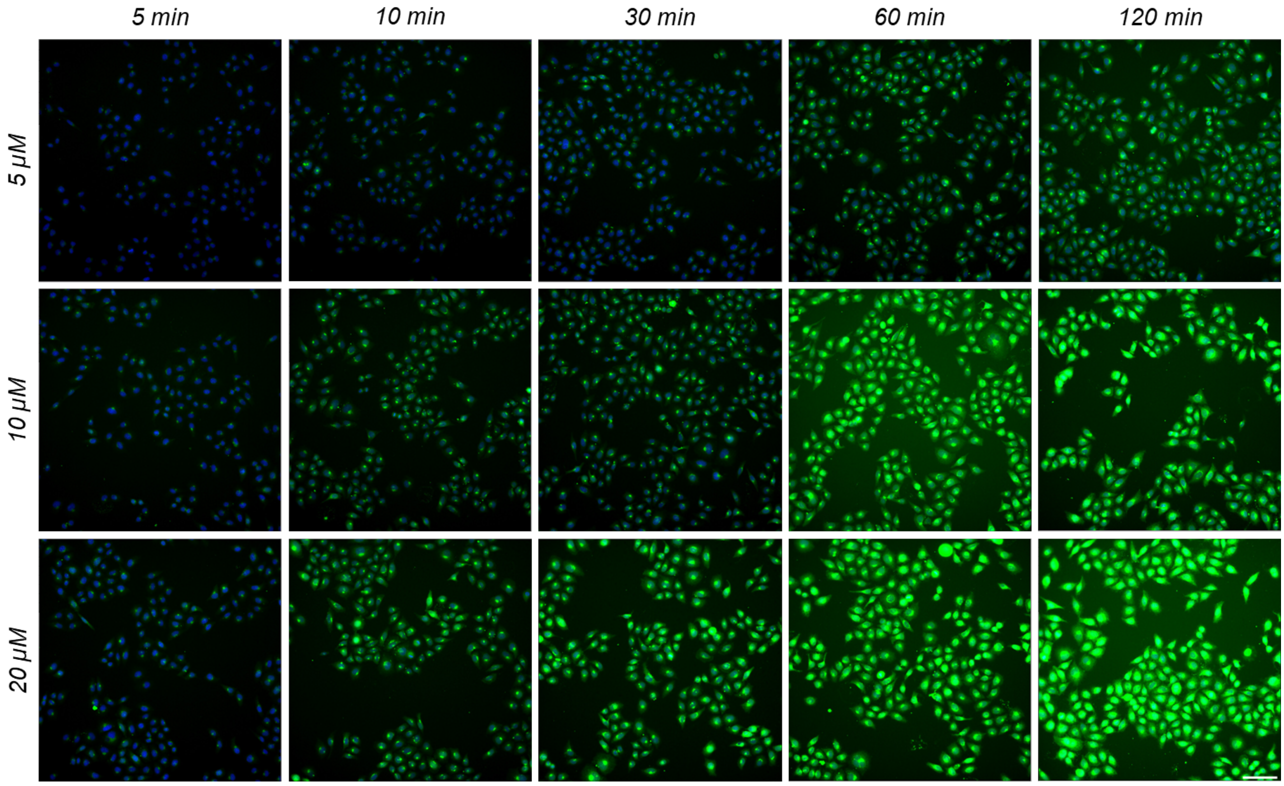

Figure S3. AHCHT microscope images (10X, widefield) of HK cells in the 96-well plate after 5, 10, 30, 60 or 120 min incubation with 5 to 20 µM of **6** (green; blue: Hoechst 33342; scale bar 200 µm).

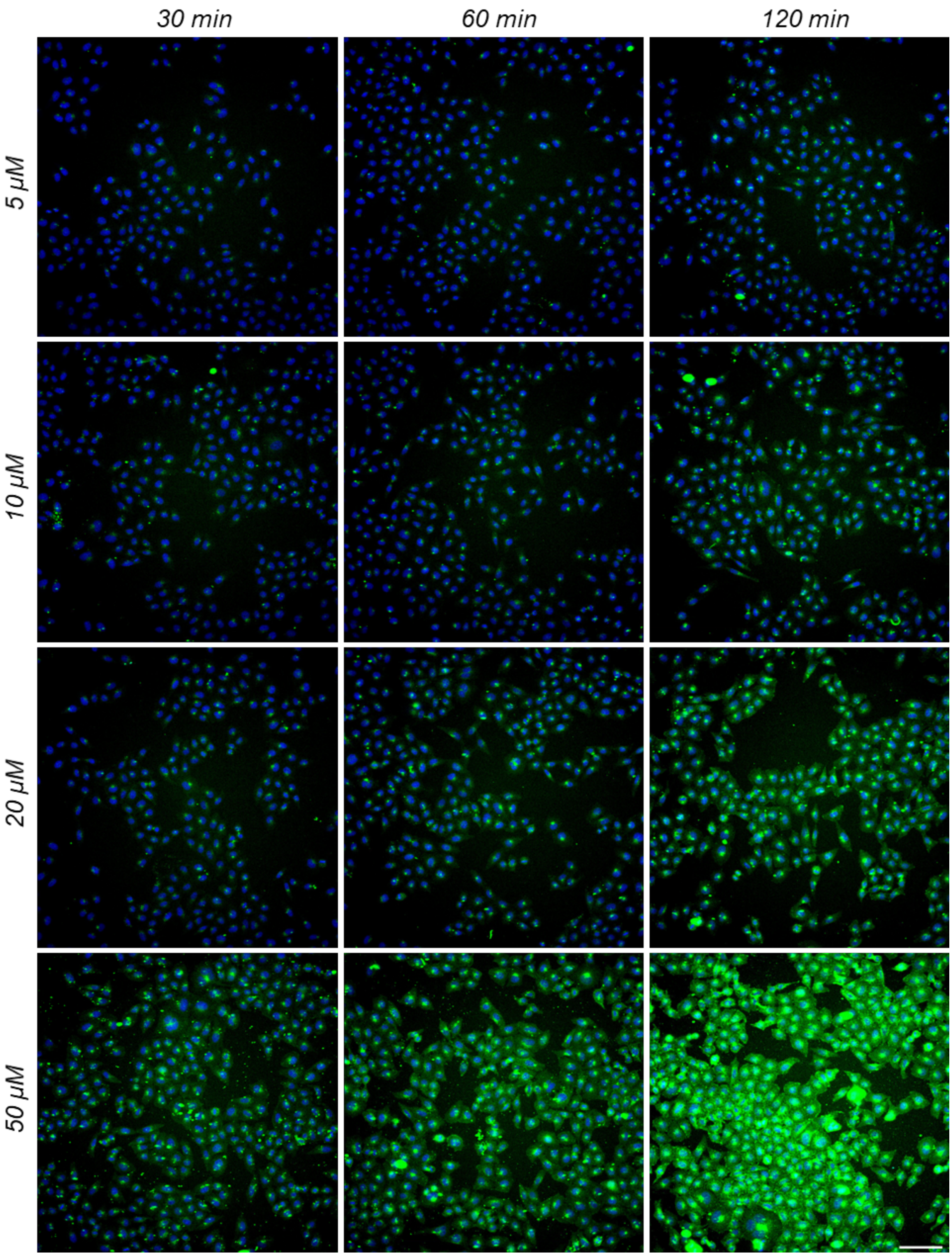

Figure S4. AHCHT microscope images (20X, widefield) of HK cells in the 96-well plate after 30, 60 or 120 min incubation with 5 to 50 µM of **8** (green; blue: Hoechst 33342; scale bar 100 µm).

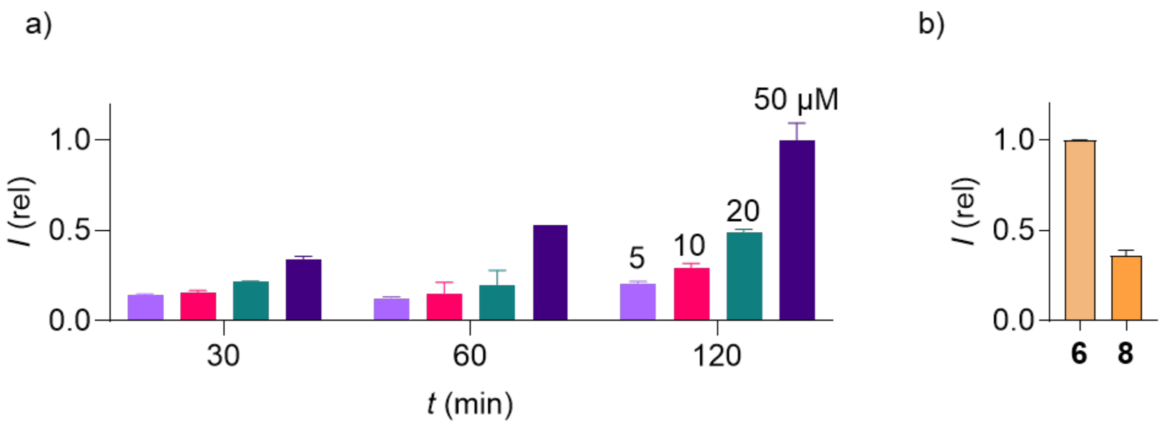

Figure S5. a) AHCHT data showing the relative fluorescence intensity (I) ± SD of compound **8** in different concentration and incubation times and b) versus **6** (10 µM, 1 h).

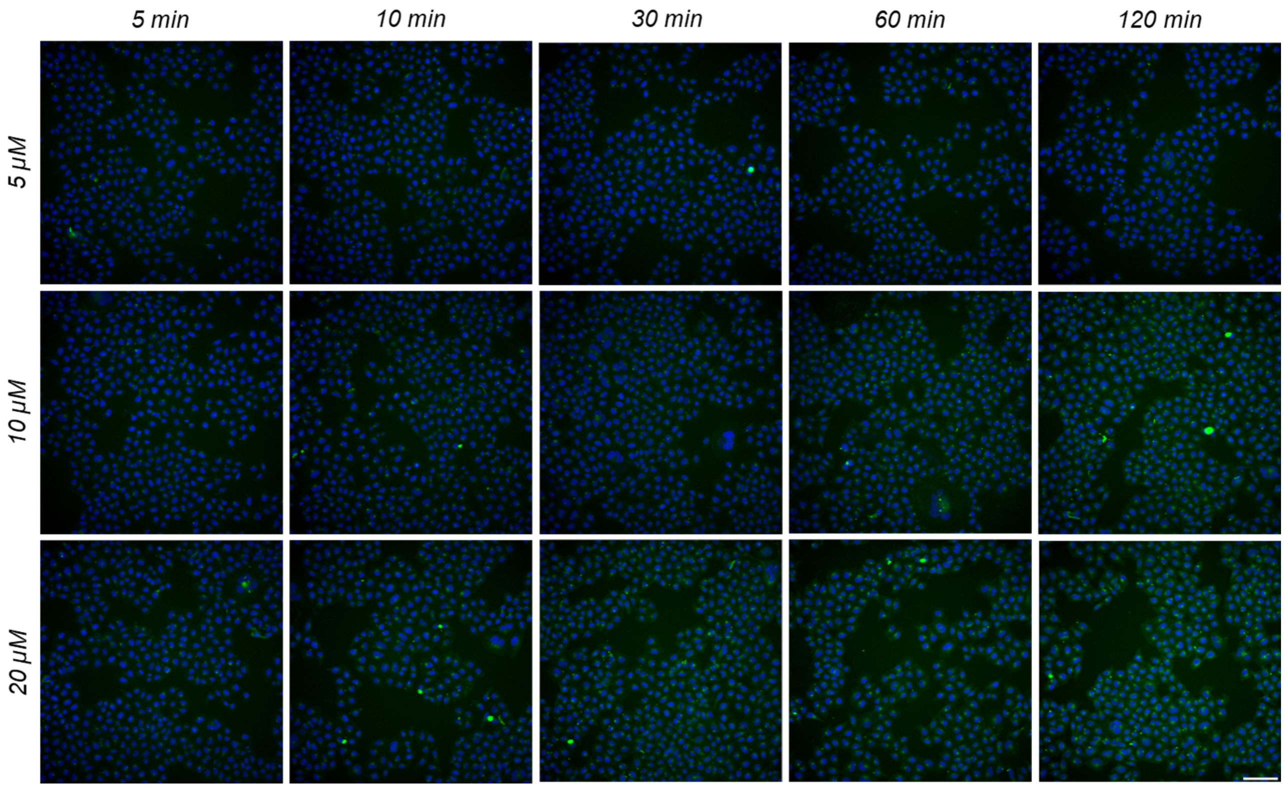

Figure S6. AHCHT microscope images (10X, widefield) of HK cells in the 96-well plate after 5, 10, 30, 60 or 120 min incubation with 5 to 20 µM of **9** (green; blue: Hoechst 33342; scale bar 200 µm).

5.4. SiR-AspA and MaP555-AspA

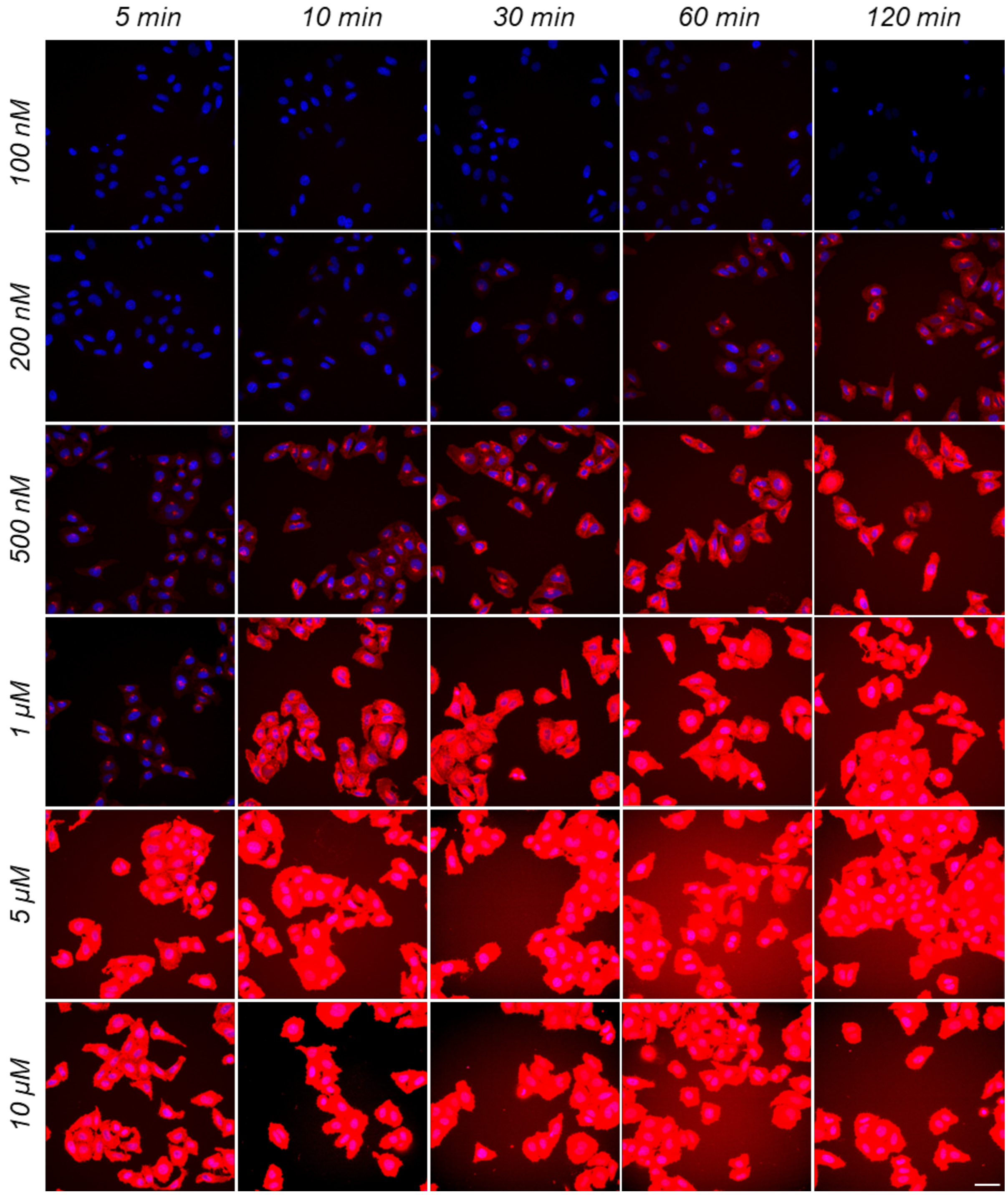

Figure S7. AHCHT microscope images (40X WI, confocal) of HK cells in the 96-well plate after 5, 10, 30, 60 or 120 min incubation with 0.1, 0.2, 0.5, 1, 5 or 10 μM of **1** (red; blue: Hoechst 33342; scale bar 50 μm).

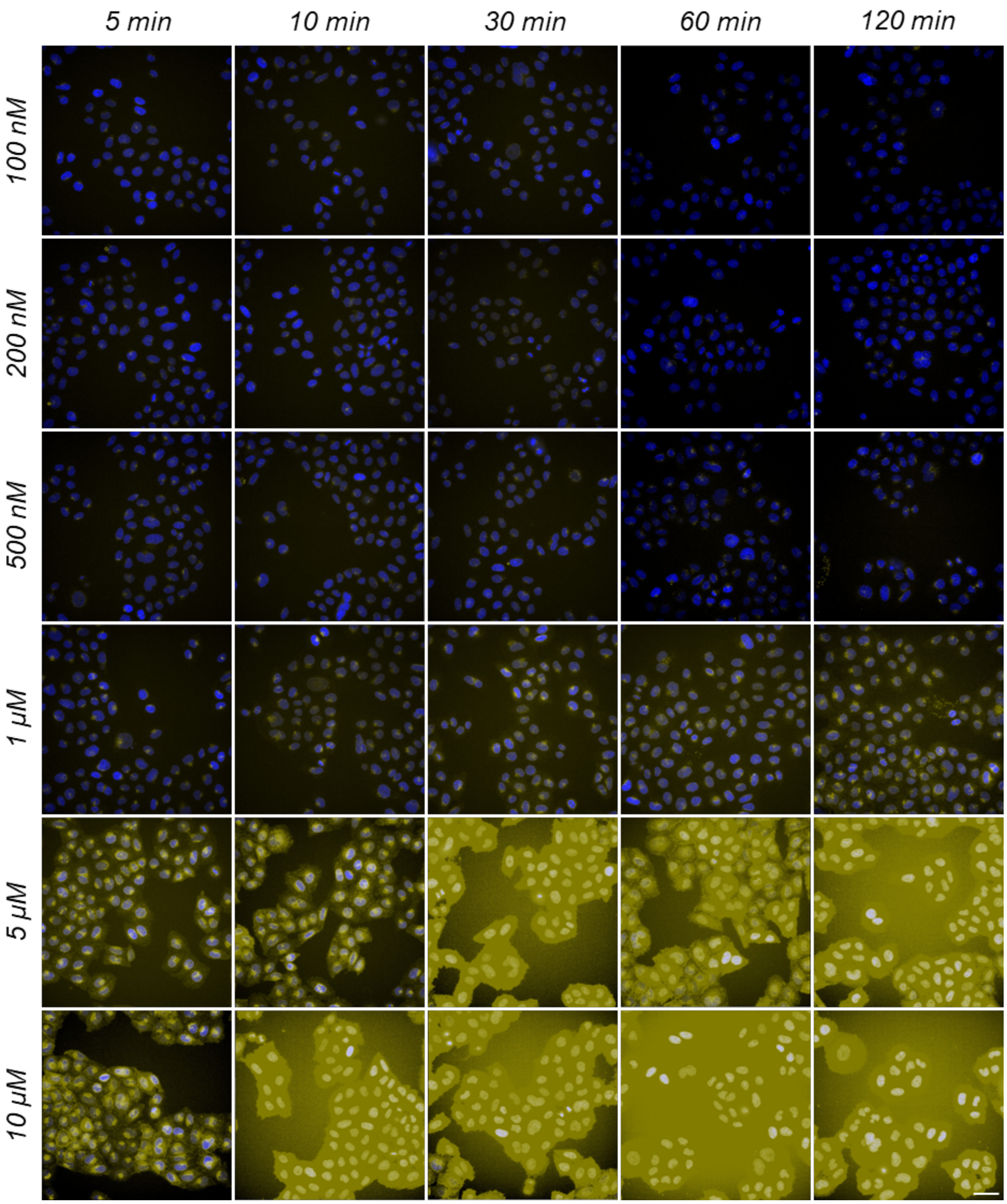

Figure S8. AHCHT microscope images (40X WI, confocal) of HK cells in the 96-well plate after 5, 10, 30, 60 or 120 min incubation with 0.1, 0.2, 0.5, 1, 5 or 10 μM of **7** (yellow; blue: Hoechst 33342; scale bar 50 μm).

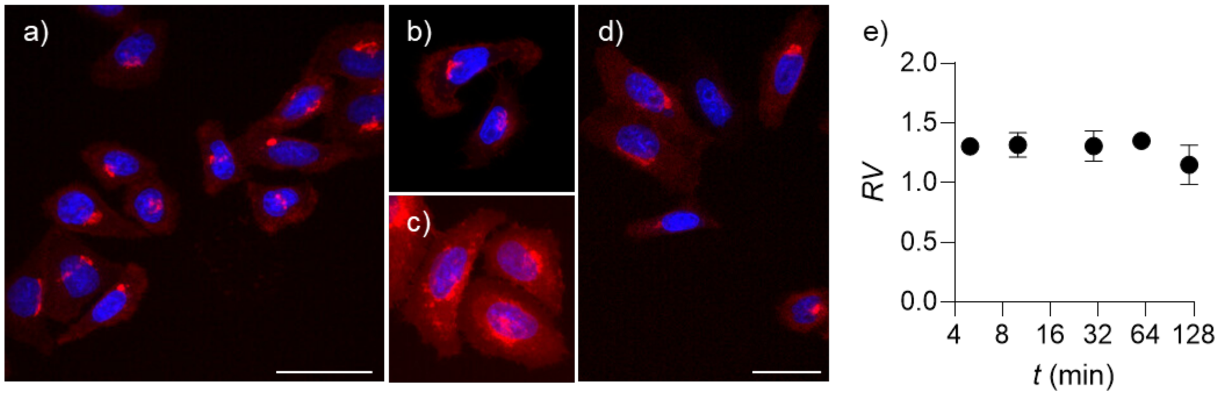

Figure S9. SDCM images (40X WI) with adjusted brightness of **1** at a) 500 nM (5 min), b) 10 µM (5 min), c) 10 µM (120 min), and d) 100 nM (60 min) (red; blue: Hoechst 33342; scale bar 50 µm for a) and 30 µm for d)). e) The respective cell viability ($RV_{\mathrm{rel}} \pm$ SD) at 10 µM of **1** as a function of time.

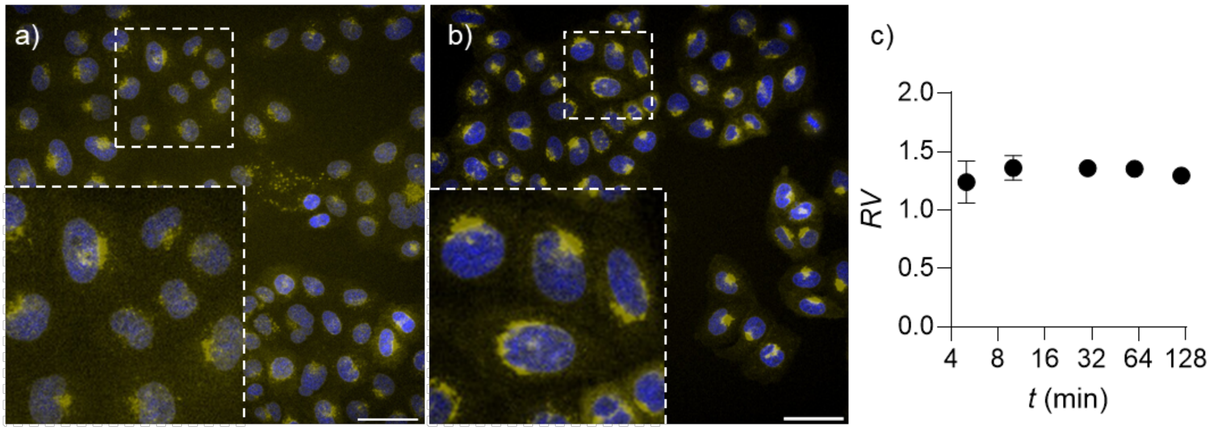

Figure S10. SDCM images (40X WI) with adjusted brightness of **7** at a) 1 µM (10 min) and b) 10 µM (5 min, yellow; blue: Hoechst 33342; scale bar 50 µm). c) The respective cell viability ($RV_{\mathrm{rel}} \pm$ SD) at 10 µM of **7** as a function of time.

5.5. SiR-AspA vs Dipalmitoylated SiR-AspA

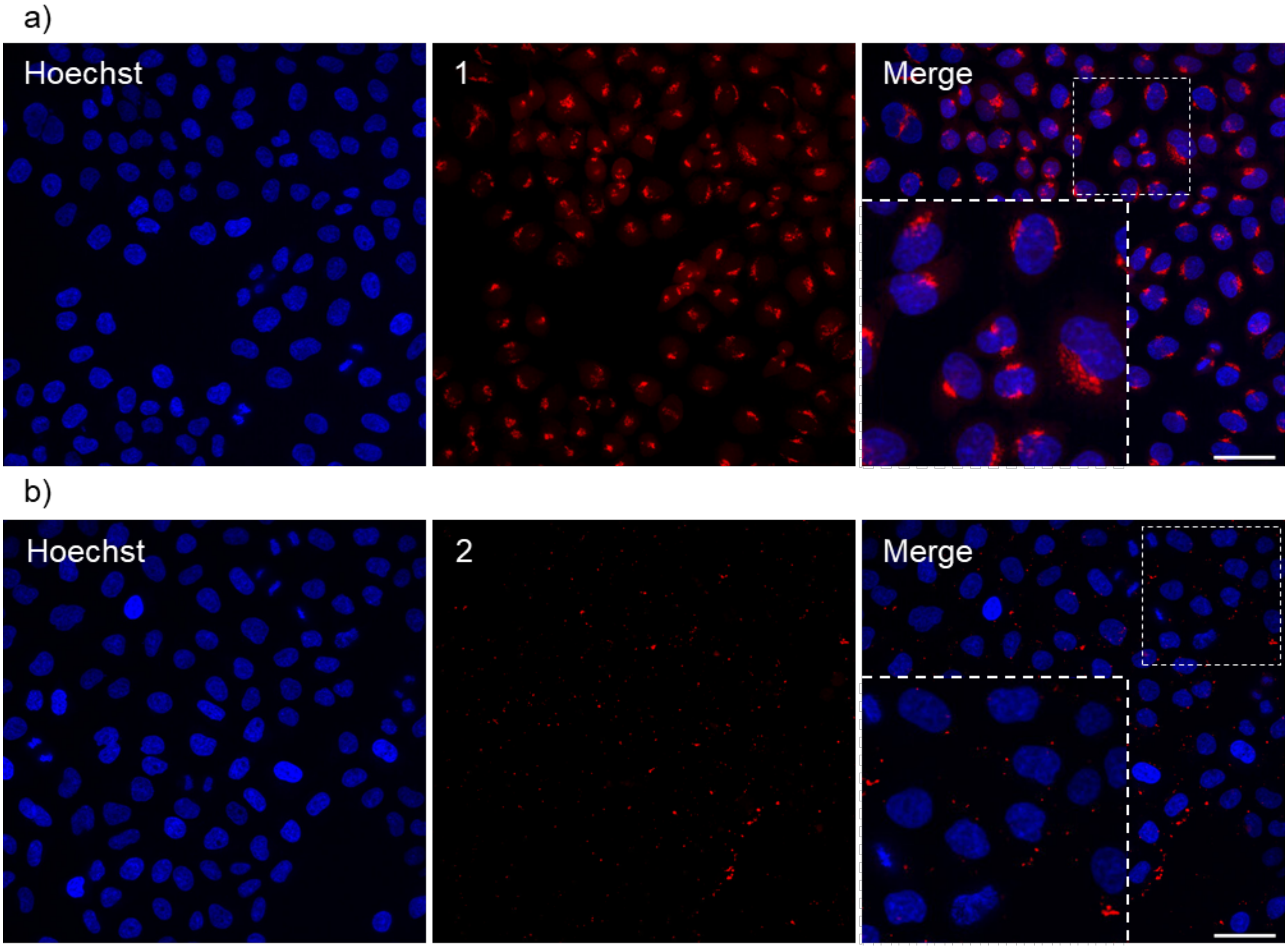

Figure S11. SDCM images (40X WI) of HK cells treated with a) **1** (500 nM, red) or b) **2** (500 nM, red) for 10 min (blue: Hoechst 33342; scale bar 50 µm).

6. Influence of Cysteine

Following the procedure described in section 5.1., except that **6** (10 µM) was co-incubated for 60 min with different concentration of L-cysteine.

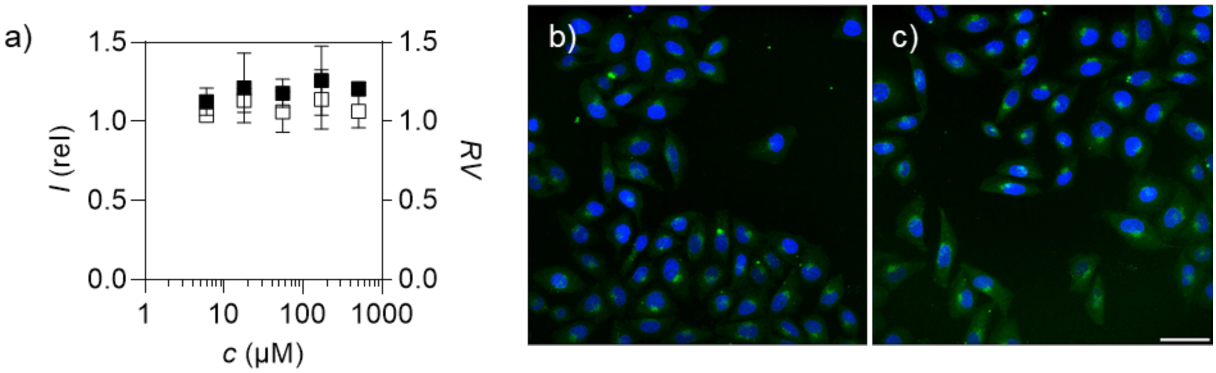

Figure S12. a) Relative fluorescence intensity *I* (rel) ± SEM of **6** (10 µM) in HK cells (filled symbols) and relative viability *RV* ± SEM (empty symbols) as a function of the concentration of L-cysteine. b,c) SDCM images (40X WI) of HK cells after incubating with **6** (green, 10 µM) b) without and c) with 500 µM of L-cysteine (blue: Hoechst 33342, nuclei; scale bar 50 µm).

7. Influence of BSA

Following the procedure described in section 5.1., except that **1** (0.5 µM) was pre-incubated for 30 min with BSA (0.5 µM) in FDMEM.

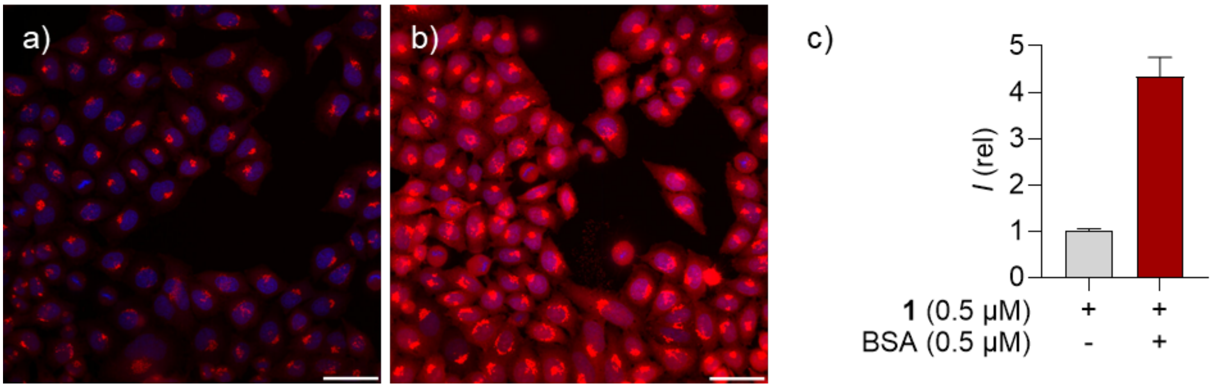

Figure S13. SDCM images (40X WI) of HK cells treated with **1** (500 nM, red) for 5 min a) without or with b) BSA (500 nM) and b) the respective intensity signals (Hoechst 33342; scale bar 50 µm).

8. Influence of Serum

Following the procedure described in section 5.1., except that serum-free FDMEM was replaced by FDMEM supplemented with 10% FCS.

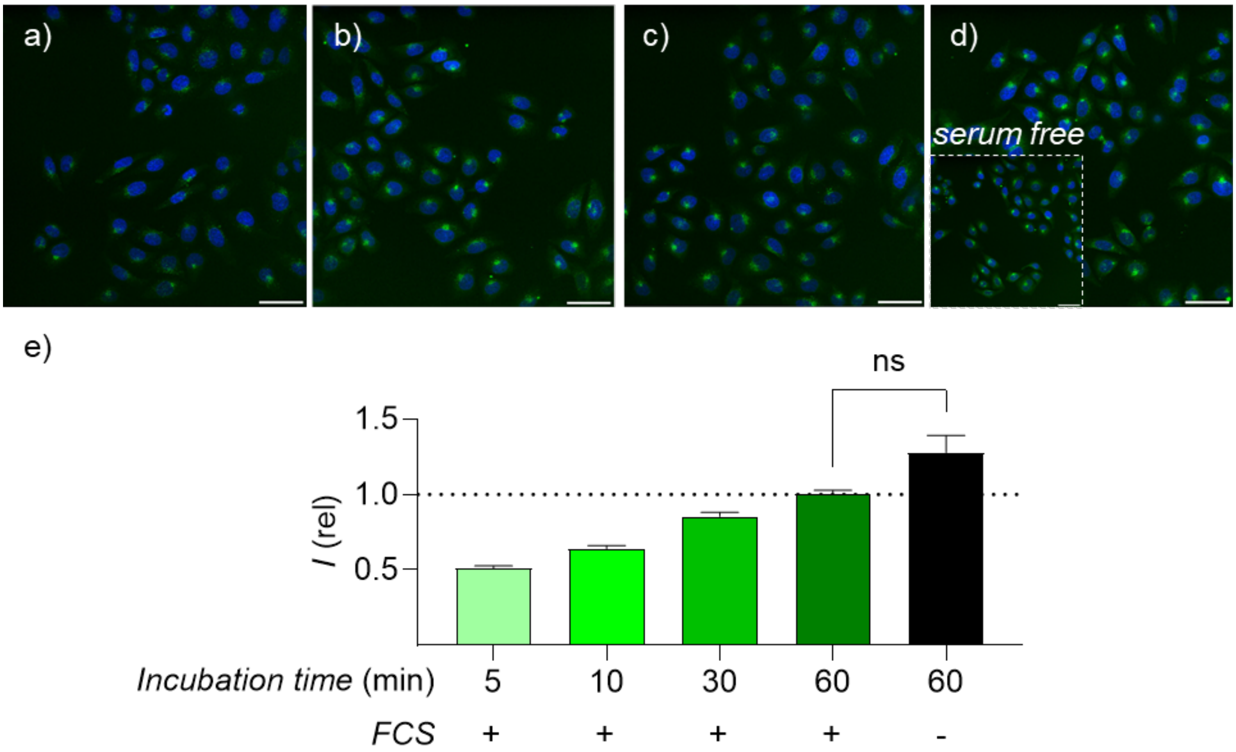

Figure S14. SDCM images (40X WI) of HK cells after incubating with **6** (green, 10 µM) for a) 5 min, b) 10 min, c) 30 min and d) 60 min in presence of 10% FCS. Blue: Hoechst 33342; scale bar 50 µm. e) AHCHT data showing the relative fluorescence intensity I_{rel} + SD of **6** at different incubation times in HK cells in presence of 10% FCS (P-value = 0.13 obtained with two-tailed unpaired t test).

9. Difference Between Live and Fixed Cells

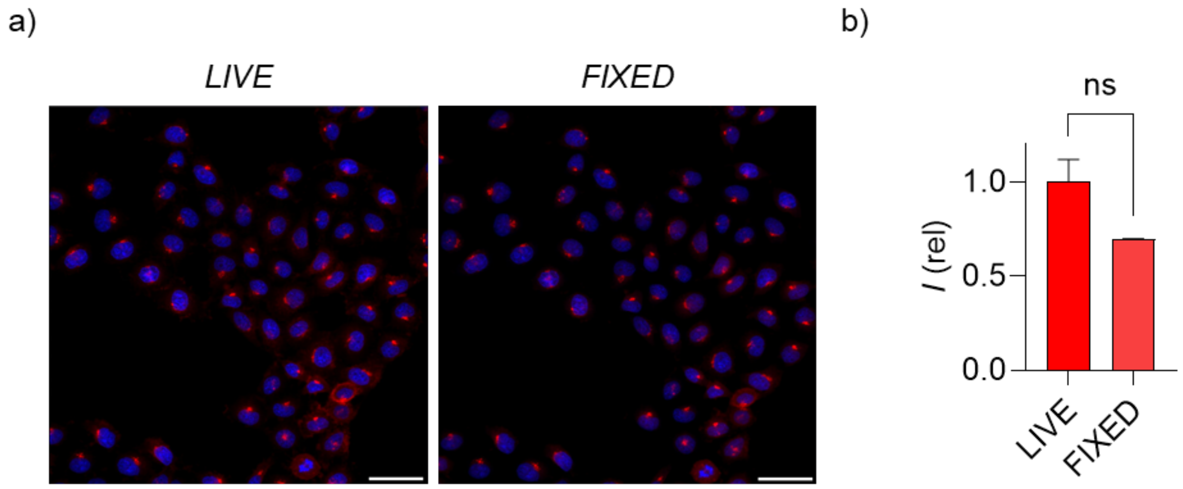

Figure S15. SDCM images (40X WI) of a) live or fixed HK cells treated with **1** (500 nM, red) for 10 min and b) the respective intensity signals between live and fixed cells (P-value = 0.13 obtained with two-tailed unpaired t test; blue: Hoechst 33342; scale bar 50 µm).

10. Dependence on Cell Lines

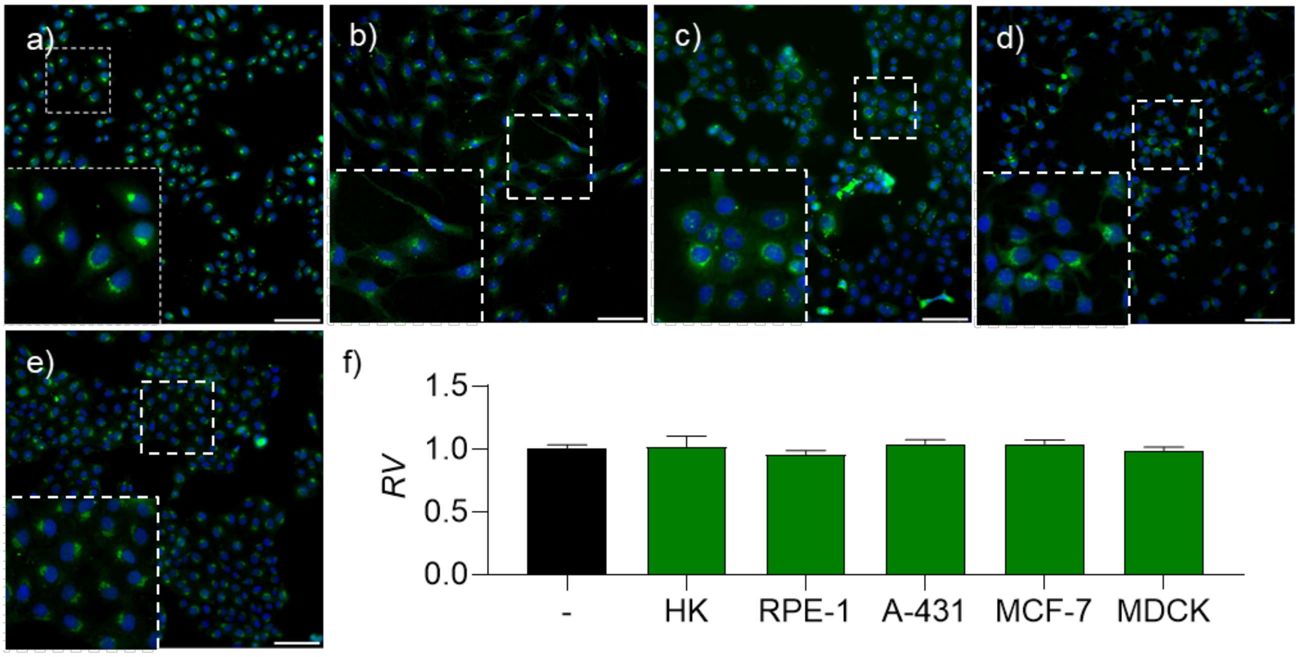

Figure S16. SDCM images (20X WI) of a) HK, b) RPE-1, c) A-431, d) MCF-7, e) MDCK after incubation with **6** (10 µM, green) for 1 h and f) the respective cell viability (RV_{rel} ± SD) (blue: Hoechst 33342; scale bar 100 µm).

11. Stability

11.1. Stock Solutions

Stock solutions of **1**, **6-9** and **11** were prepared in DMSO with a concentration range of 1-10 mM. Aliquots were prepared to avoid freeze/thaw cycles and stored at – 20 °C for at least > 6 months with no sign of degradation.

Stability of GA tracker **1** (10 µM, DMSO) at rt was monitored by LC-MS for 3 weeks with no signs of degradation. Conditions: column Hypersil Gold Vanquish 1.9 µm 2.1 x 50 mm, gradient: 5-95% CH_3CN in H_2O + 0.1% formic acid in 4 min, 0.5 mL/min. Detection wavelengths at 650 nm. After the 3 weeks, the stock solution of **1** was used for GA staining in HK cells following the procedure described in section 5.1. (depicted in Figure S18).

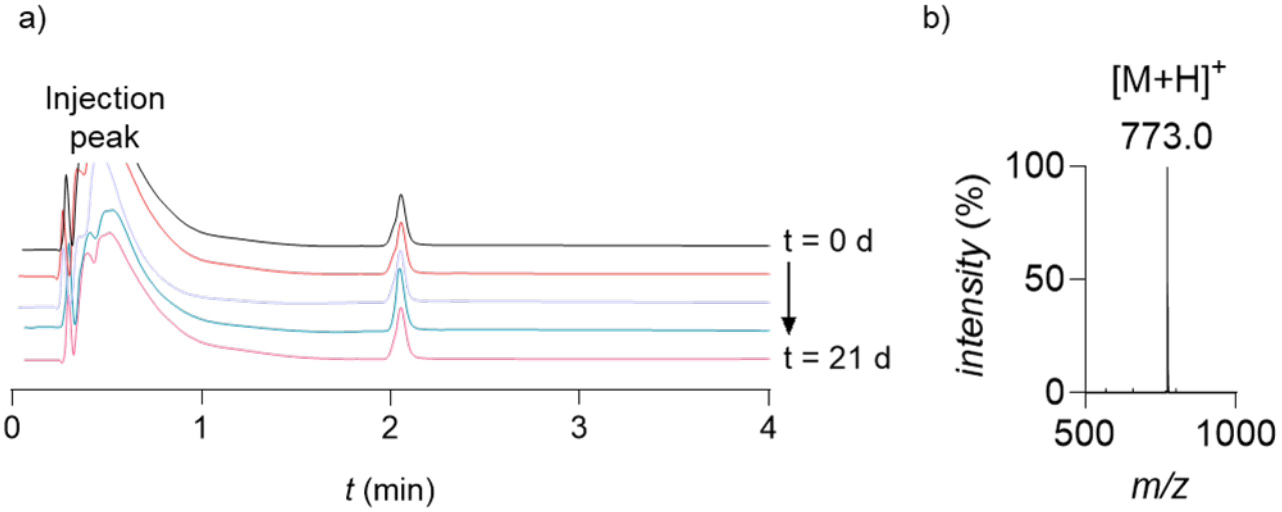

Figure S17. a) HPLC profiles of the stock solution of **1** (10 µM, DMSO) at different time points showing the stability over 3 weeks at rt b) with the corresponding mass spectrum.

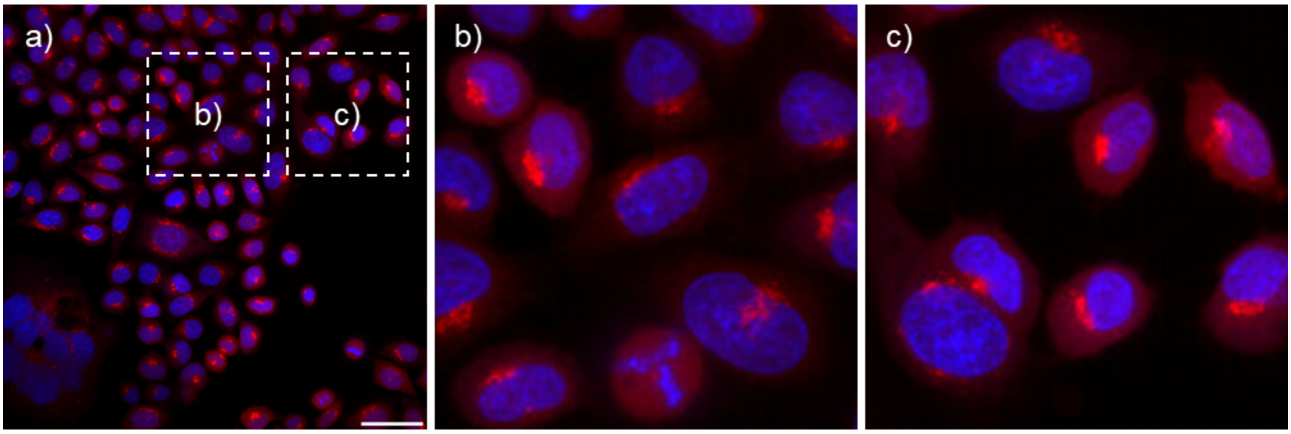

Figure S18. SDCM images (40X WI) of a) HK cells treated with the 3 weeks old stock solution of **1** (1 µM, red) for 10 minutes, b-c) and the zoomed regions. (blue: Hoechst 33342; scale bar 50 µm).

11.2. Cells

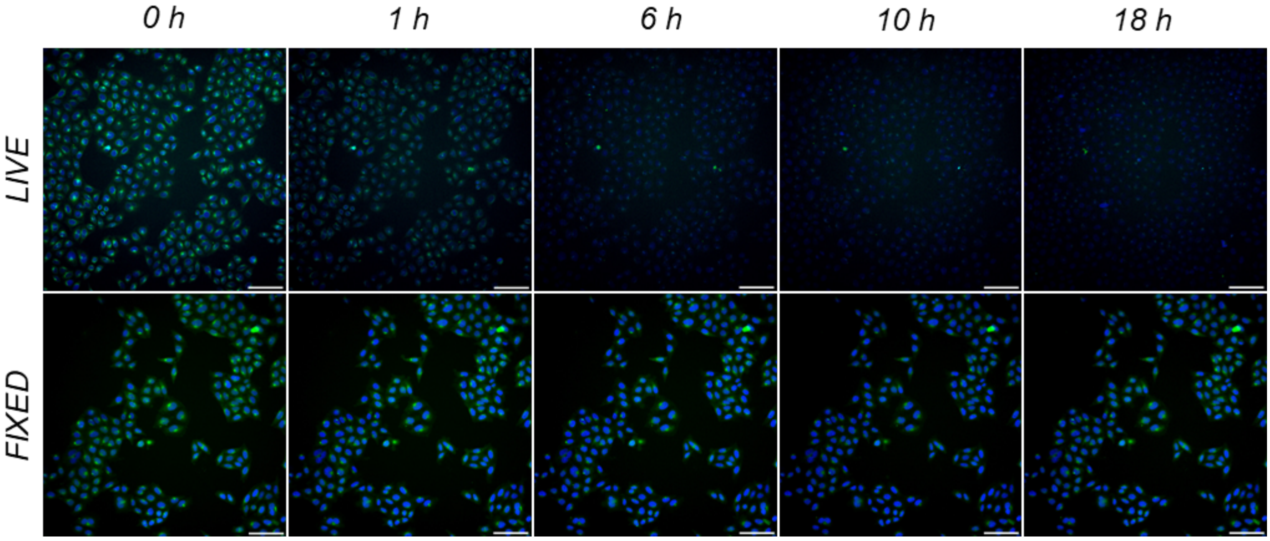

Figure S19. AHCHT images (20X WI, confocal) at different time points of HK cells treated with **6** (20 µM, green) for 10 min, rinsed, and monitored every hour for 18 h under 2 different conditions, live and fixed (blue: Hoechst 33342; scale bar 100 µm).

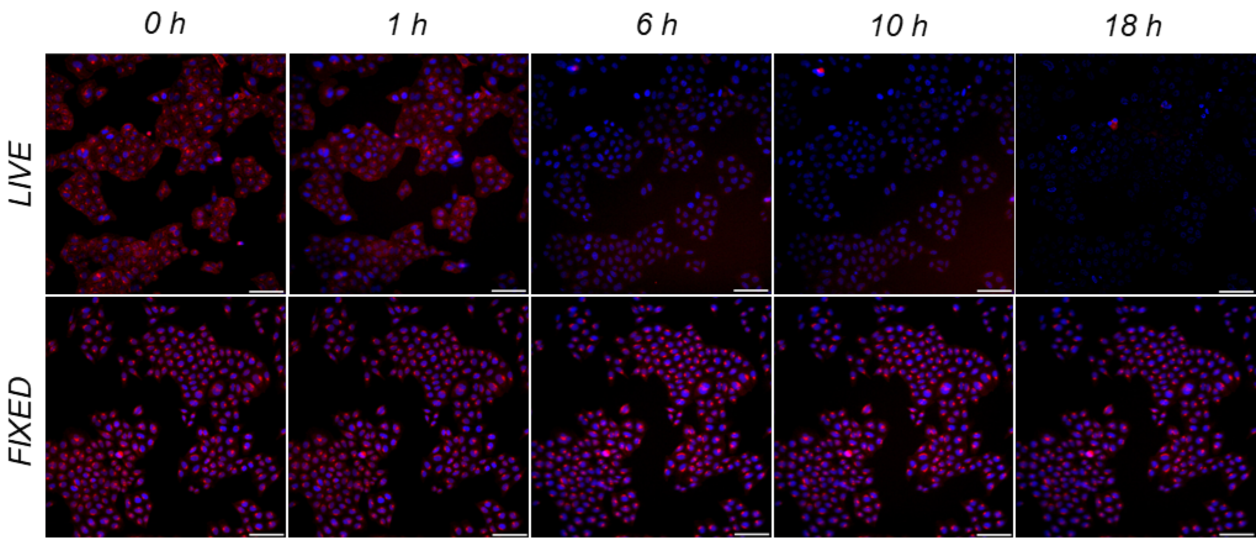

Figure S20. AHCHT images (20X WI, confocal) at different time points of HK cells treated with **1** (1 µM, red) for 10 minutes and monitored every hour for 18 h in 2 different conditions, live and fixed (blue: Hoechst 33342; scale bar 100 µm).

12. Co-Localization

12.1. ER-Trackers

Cells were prepared in a 96 well plate as described in section 4, then medium was removed. Cells were then washed with PBS (3 × 3 mL/well) followed by fresh FDMEM serum-free medium (4 × 100 µL/well) using a plate washer (Biotek EL406®), and kept in a 100 µL of the latter medium. A solution of **6** (10 mM, DMSO) and a solution of ER tracker™ red (Thermo Fisher E34250; 1 mM, DMSO) were diluted in FDMEM to give a solution at 6x final concentration, of which 25 µL of each was co-added to the well resulting in a final volume of 150 µL per well (final concentration: **6**, 10 µM, ER tracker™, 1 µM). The cells were incubated under 5% CO_2 humidified atmosphere at 37 °C for 30 min. Afterward, the cells were washed with PBS (3 × 3 mL/well) and the medium was exchanged with FDMEM keeping a final volume of 100 µL/well, and a solution of Hoechst 33342 (100 µg/mL) in PBS (50 µL/well) was added. After 10 min of incubation under 5% CO_2 humidified atmosphere at 37 °C, cells were washed with PBS (3 × 3 mL/well) and kept in FDMEM (100 µL/well) for live cell imaging. The distribution of fluorescent signals was captured on a IXM-C automated

microscope with two channels, blue for Hoechst 33342 (377/50 nm excitation filter; 477/60 nm emission filter), green for **6** (475/34 nm excitation filter; 536/40 nm emission filter) or red for ER tracker™ (590/40 nm; emission filter: 615/50 nm). Duplicates were performed for each condition.

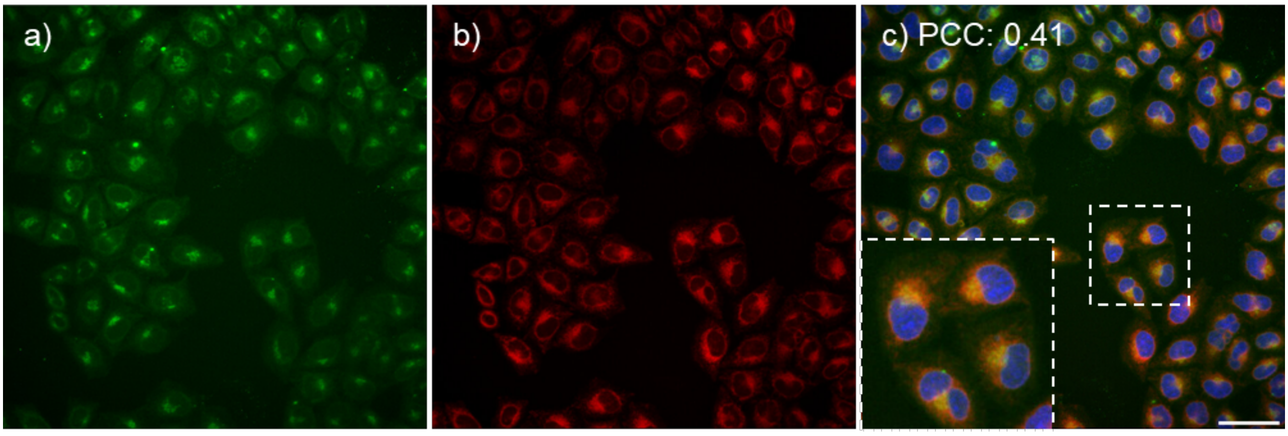

Figure S21. SDCM images (40X WI, confocal) of HK cells co-stained with **6** and ER tracker™ in a) green channel for **6** (10 μM), b) red channel for ER tracker™ (1 μM) and c) merged with the PCC value (blue: Hoechst 33342, nuclei; scale bar 50 μm).

12.2. Anti-Golgi Antibodies

After the cells were treated with the AspA derivatives **6** (20 μM, 60 min) or **1** (0.5 μM, 10 min) following the procedure described in section 5.1., the cells were treated with a solution of PFA 3% (70 μL/well) for 15 min at rt. The fixed cells were washed with PBS (9 x 3 mL/well) and then incubated with the corresponding primary antibody (anti-GM130 (mouse), anti-GOLPH3 (rabbit) or anti-TGN46 (rabbit)) in PBS containing 1% BSA and 0.05% of saponin (70 μL per well, 1/500 for GM130 and GOLPH3 and 1/400 for TGN46; stock solution of Ab: GM130, 0.25 mg/mL; GOLPH3, 0.80 mg/mL; TGN46, 0.80 mg/mL) for 1 h at rt. The cells were washed with PBS (9 × 3 mL/well) using the plate washer and treated with a solution of the secondary antibody with a fluorescent dye in PBS (80 μL per well, 1/200 of 1 mg/mL; 4 secondary antibodies: Alexa Fluor® 488 and 647 AffiniPure Donkey Anti-Mouse IgG (H+L), Alexa Fluor® 488 and 647 AffiniPure Donkey Anti-Rabbit IgG (H+L)) for 1 h at rt. The distribution of fluorescent signals was captured on a IXM-C automated microscope with three channels, blue for Hoechst 33342 (377/50 nm excitation filter;

477/60 nm emission filter), green (475/34 nm excitation filter; 536/40 nm emission filter) and red (620/50 nm; emission filter: 690/50 nm). The rest of the parameters were adjusted according to the nature of the experiment. Triplicates were performed for each condition.

12.3. Data Analysis

For the quantification of the fluorescent signal and/or ratio of signals in the GA, a new method was developed and applied. Briefly, the blue channel (Hoechst 33342) was used for the segmentation of the nuclei and cell bodies (Figure S22).

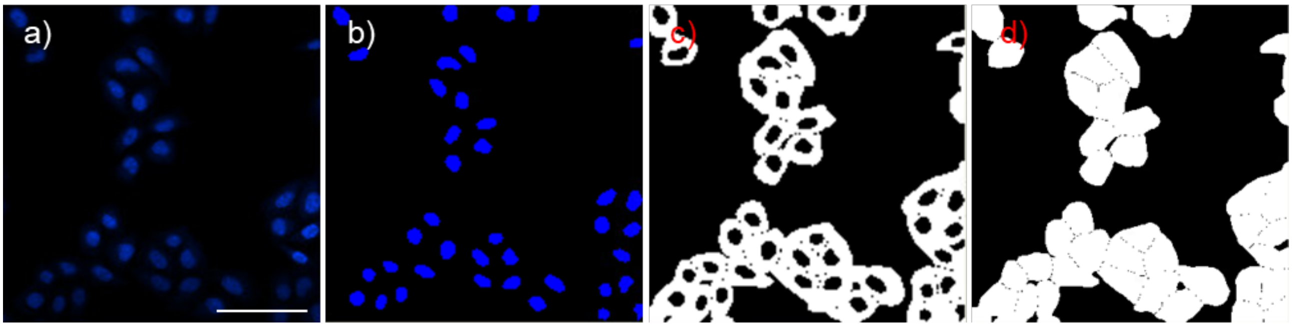

Figure S22. a) SDCM image of the blue channel (Hoechst 33342), b) nuclei mask, c) cell body without nucleus mask and d) cell body mask (scale bar 100 μm).

To determine the area sum of the GA, a *Top-hat* transformation was applied to the green channel (GM130 staining), filtered out by the size (10 pixels) and brightness (lower threshold 500) (Figure S23).

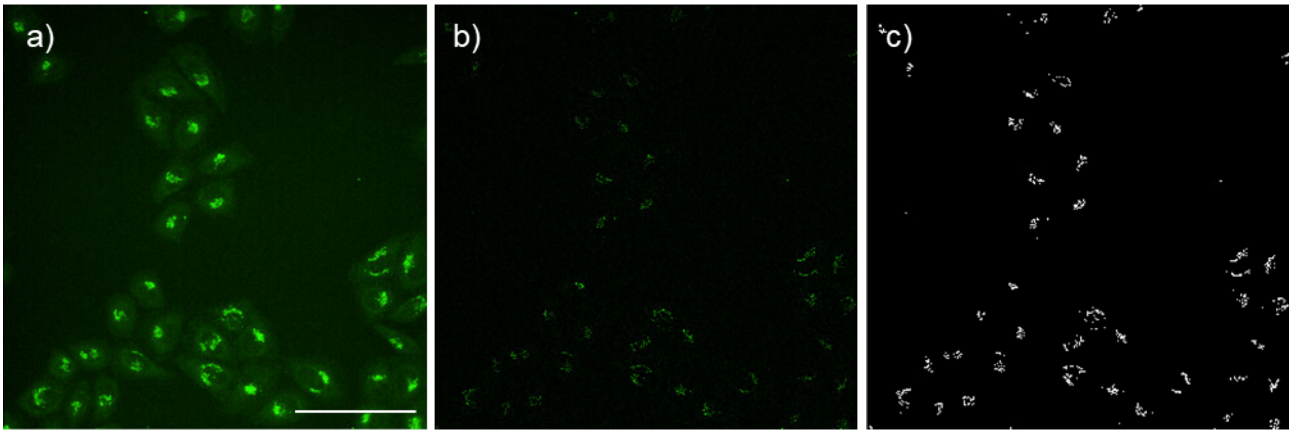

Figure S23. a) SDCM images of the green channel (GM130), b) *Top-hat* transformation of (a) filtered out by size and intensity generating c) the Golgi mask. Scale bar 100 µm

The same operation was performed in the red channel to generate the corresponding GA area sum labeled by the compound **1** (Figure S24).

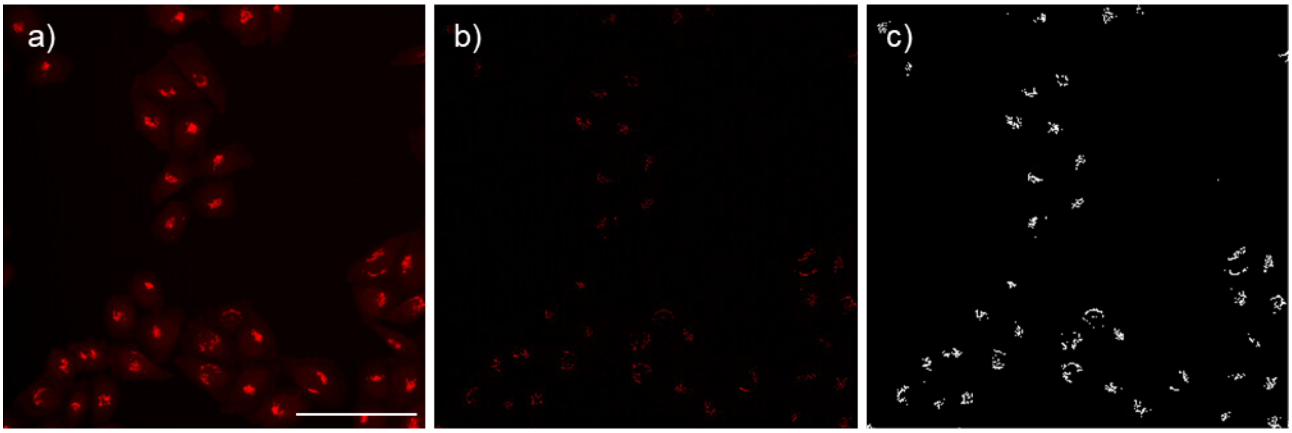

Figure S24. a) SDCM images of the red channel (**1**), b) *Top-hat* transformation of (a) filtered out by size and intensity generating c) the Golgi mask. Scale bar 100 µm

A final co-positive mask was applied to determine the ratio of co-localization: both previous masks for GM130 and **1** (Figure S23 and S24 respectively) were overlapped, and the puncta that overlap or touch one another are highlighted in yellow (Figure S25). The count of yellow dots over the count of red dots gave the ratio of co-localization (CoR).

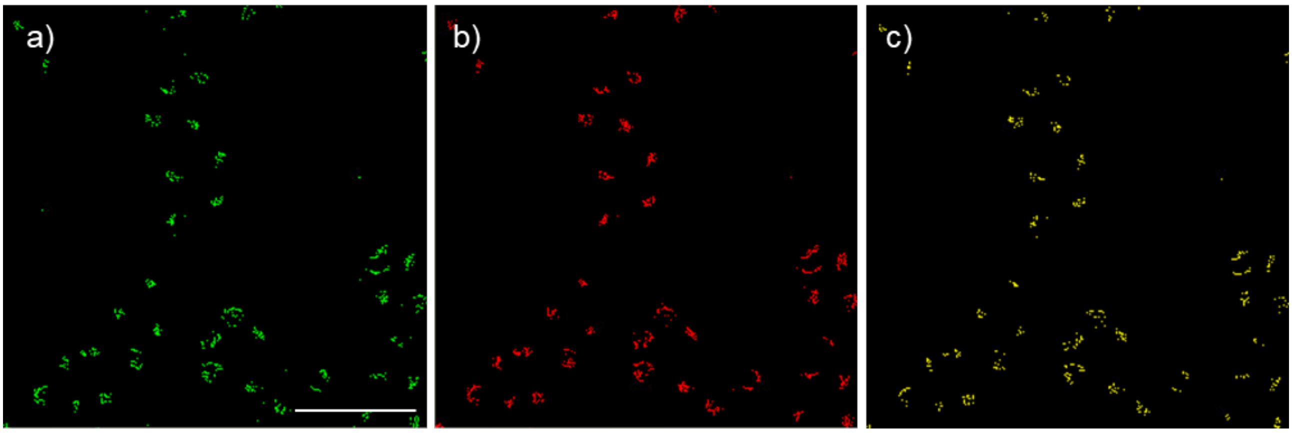

Figure S25. The generated masks for a) GM130 IF staining, b) AspA derivative **1** and c) the overlay of a) and b).

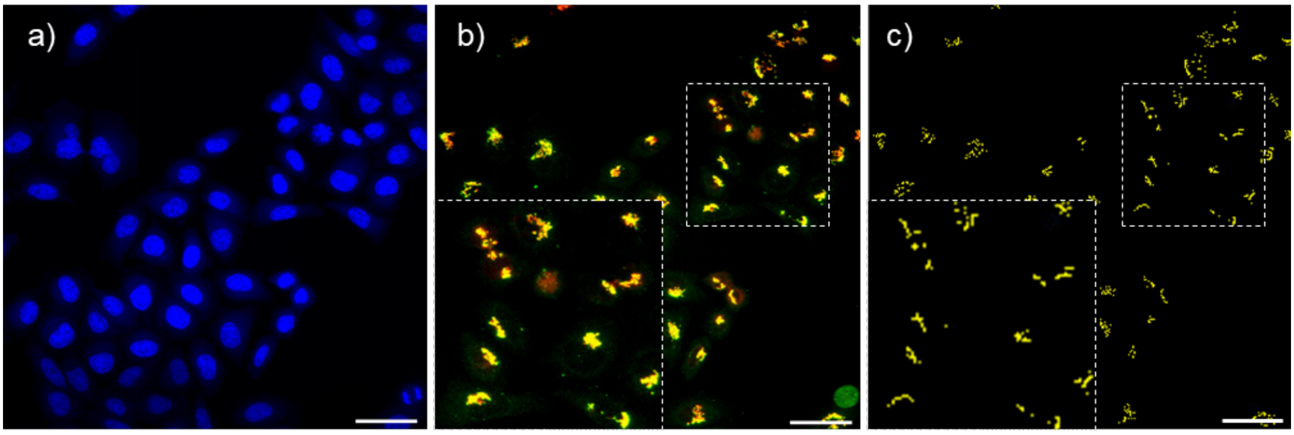

Figure S26. SDCM images (40X WI) of HK cells with a) Hoechst 33342, b) overlay of **1** (red, 500 nM) and IF of GM130 (AlexaFluor® 488-conjugated AffiniPure Donkey Anti-Mouse IgG (H+L)), green) and c) the corresponding CoR mask (scale bar 50 µm).

12.4. Golgi Proteins

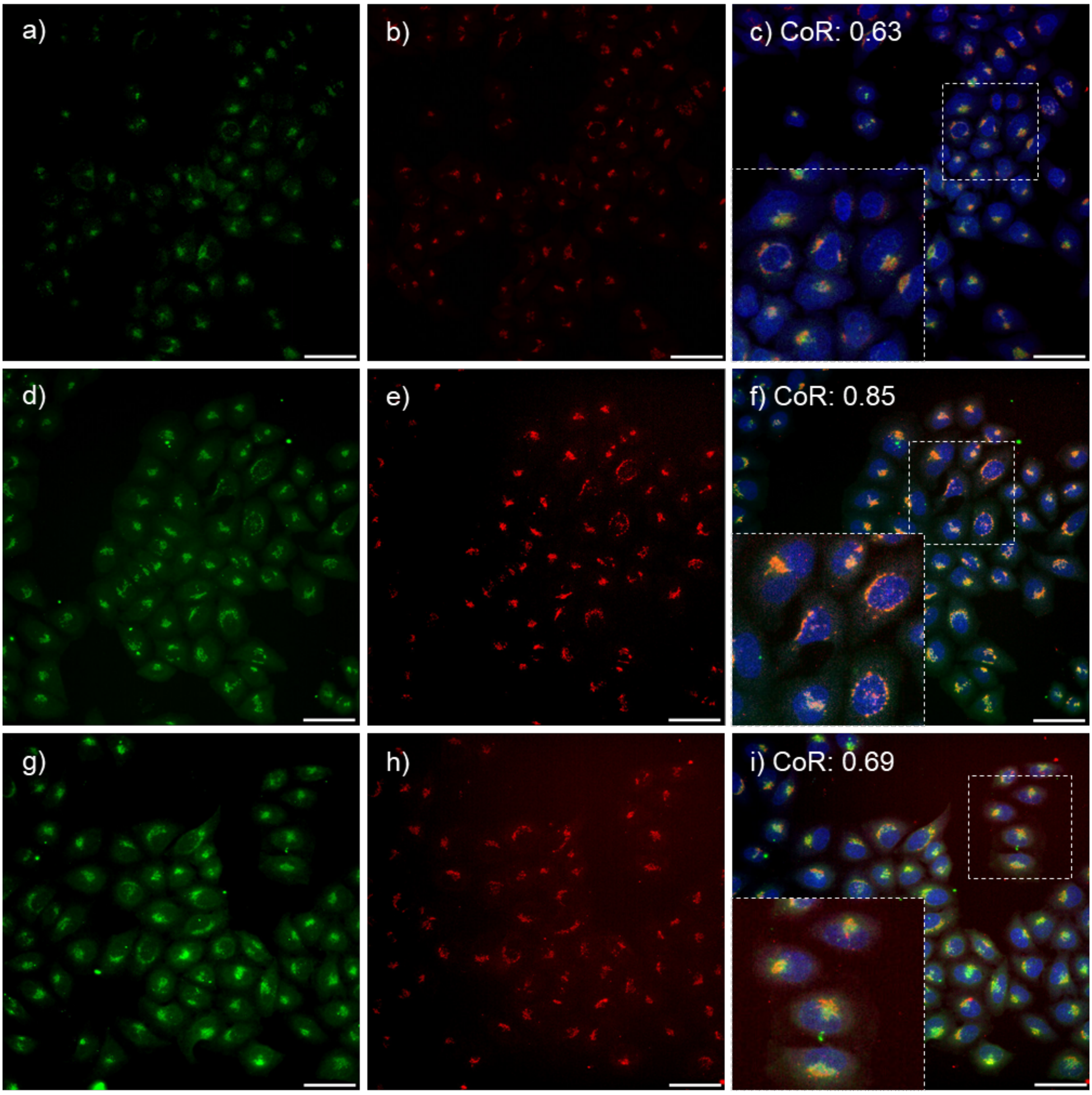

Figure S27. SDCM images (40X WI) of Golgi apparatus staining with the CoR values in HK cells with a) **6** (10 µM, green), b) IF of GOLPH3 (AlexaFluor® 647-conjugated AffiniPure Donkey Anti-Rabbit IgG (H+L)), red), c) both (merged), d) **6** (20 µM, green), e) IF of GM130 (AlexaFluor® 647-conjugated AffiniPure Donkey Anti-Mouse IgG (H+L)), red), f) both (merged), g) **6** (20 µM, green), h) IF of TGN46 (AlexaFluor® 647-conjugated AffiniPure Donkey Anti-Rabbit IgG (H+L)), red) and i) both (merged) (blue: Hoechst 33342, nuclei; scale bar 50 µm).

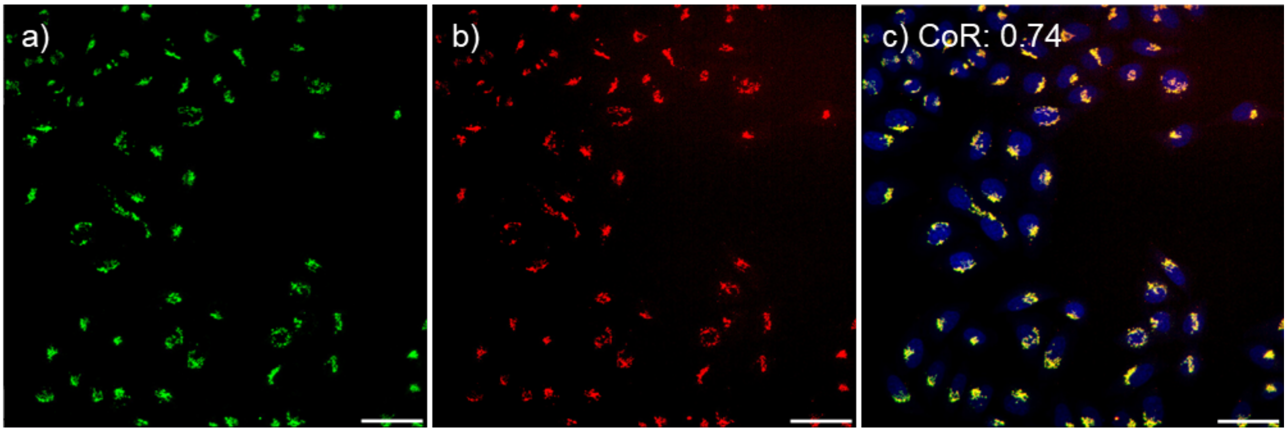

Figure S28. SDCM images (40X WI) of Golgi apparatus staining in HK cells with a) IF of TGN46 (AlexaFluor® 488-conjugated secondary antibody, green), b) IF of GM130 (AlexaFluor® 647-conjugated AffiniPure Donkey Anti-Mouse IgG (H+L)), red), and c) both (merged) with the CoR value. (blue: Hoechst 33342, nuclei; scale bar 50 µm).

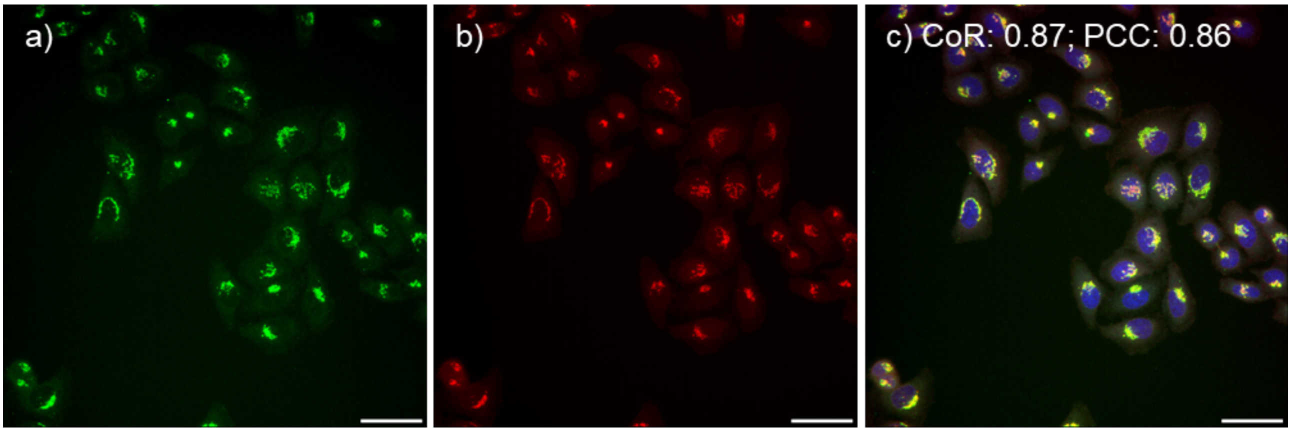

Figure S29. SDCM images (40X WI) of Golgi apparatus staining in HK cells with a) IF of GM130 (AlexaFluor® 488-conjugated AffiniPure Donkey Anti-Mouse IgG (H+L)), green), b) **1** (500 nM, red), and c) both (merged) with the CoR and PCC values. (blue: Hoechst 33342, nuclei; scale bar 50 µm).

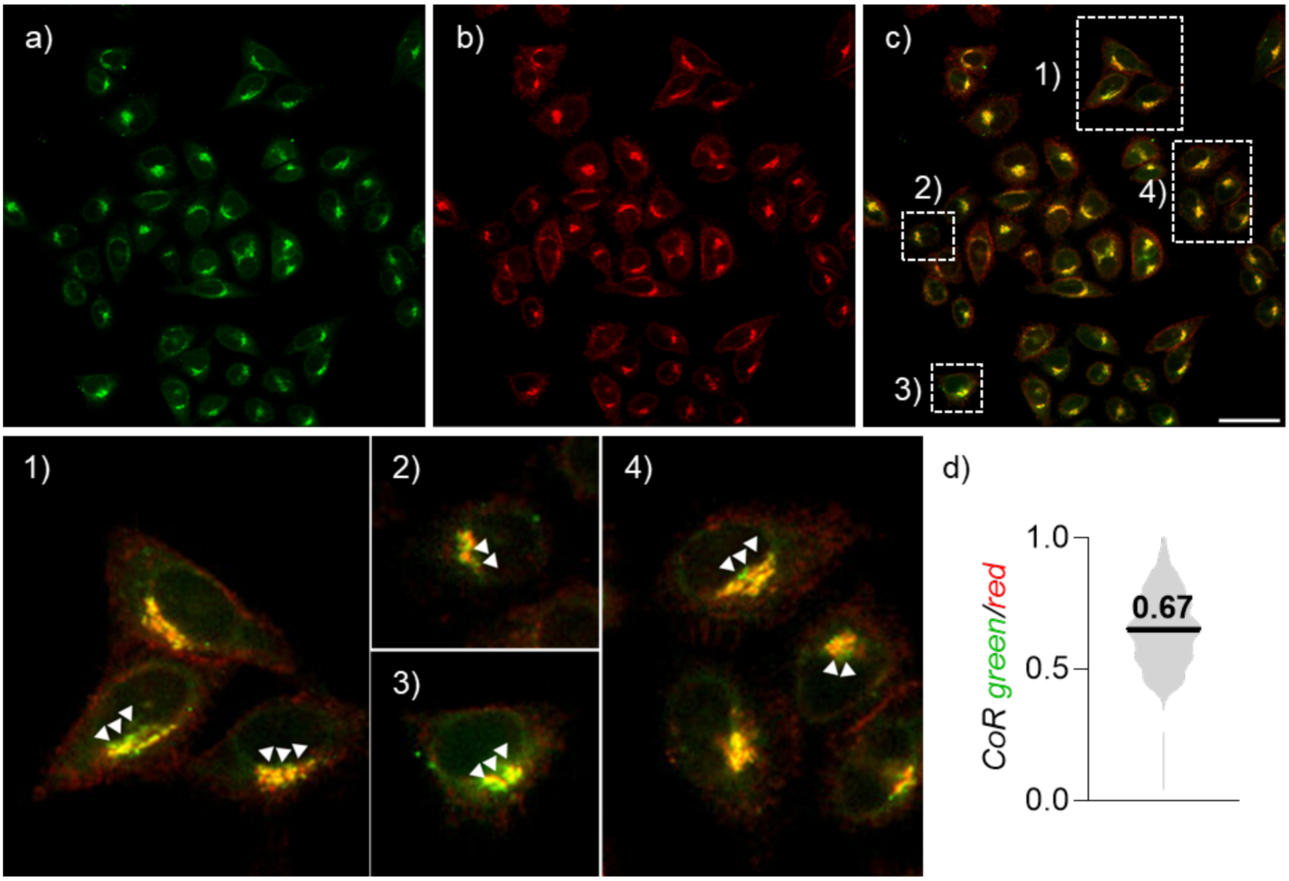

Figure S30. SDCM images (40X WI) of Golgi apparatus staining with a) **6** (25 µM, green), b) **1** (5 µM, red), and c) both (merged) with zoomed regions (1-4) showing the co-localized uptake in HK cells after incubating for 30 min at 37 °C. d) AHCHT data showing the ratio of co-localized **1** and **6** in more than 1000 cells (scale bar 50 µm).

12.5. Summary Table

Table S1. Co-localization results for AspA trackers.

Entry	Tracker 1	Tracker 2	CoR[a]	PCC[b]
1	**1**	GM130	0.87 ± 0.07	0.86 ± 0.02
2	GM130	TGN46	0.74 ± 0.03	-
3	**6**	ER-Tracker	-	0.41 ± 0.02
4		GM130	0.85 ± 0.01	-
5		TGN46	0.69 ± 0.01	-
6		GOLPH3	0.65 ± 0.01	0.63 ± 0.01
7		**1**	0.67 ± 0.02	-

[a]Co-localization ratio determined using the aforementioned metaXpress masks. [b]Pearson Correlation Coefficient (PCC) determined using ImageJ "coloc 2" plugin. - Not determined. Error corresponds to SD.

13. Golgi Morphology

13.1. Disassembly

Cells were prepared in a 96 well plate as described in section 4, then medium was removed, and cells were washed with PBS (3 × 3 mL/well) followed by complete FDMEM medium (10% FCS, 5% PS, 5% GlutaMAX, 4 × 100 µL/well) using a plate washer (Biotek EL406®), and kept in a 100 µL of the latter medium. A solution of Hoechst 33342 (50 µg/mL) in complete FDMEM (50 µL/well) was added to cells and left incubating for 30 min under 5% CO_2 humidified atmosphere at 37 °C. Afterward, the cells were washed with PBS (3 x 3 mL/well) and the medium was exchanged with complete FDMEM keeping a final volume of 100 µL/well, and a solution of **1** (1.5 µM) in complete FDMEM (50 µL/well) was added (final concentration of 0.5 µM). After 10 min of incubation under 5% CO_2 humidified atmosphere at 37 °C, cells were washed with PBS (3 × 3 mL/well) and kept in complete FDMEM (100 µL/well) for live cell imaging (imperatively, the temperature in the

microscope was kept at 37 °C and under 5% CO_2). The distribution of fluorescent signals was captured on a IXM-C automated microscope with two channels, blue for Hoechst 33342 (377/50 nm excitation filter; 477/60 nm emission filter), red (620/50 nm; emission filter: 690/50 nm) for the fluorescent reporters **1**. After the image at t_0 was captured, a solution of BFA (5 μM) or DMSO in complete FDMEM (50 μL/well) was added to cells and measurements were done every 10 min for 2 h.

13.2. Fusion with ER

Following the procedure described in section 5.1. Cells were prepared in a 96 well plate as described in section 4, then medium was removed, and cells were washed with PBS (3 × 3 mL/well) followed by fresh FDMEM serum-free medium (4 × 100 μL/well) using a plate washer (Biotek EL406®), and kept in a 100 μL of the latter medium. A solution of BFA (50 mM, DMSO) was diluted in FDMEM to give a solution at 3x final concentration (30 μM) of which 50 μL was added to the well resulting in a final volume of 150 μL per well (final concentration of 10 μM). The cells were incubated under 5% CO_2 humidified atmosphere at 37 °C for 1 h. Afterward, the cells were washed with PBS (3 x 3 mL/well) and the medium was exchanged with FDMEM keeping a final volume of 100 μL/well. Then, **1** (1.5 μM in FDMEM) was added (50 μL/well) to the cells, giving a final volume of 150 μL/well and a final concentration of 0.5 μM. The cells were incubated for an additional 10 minutes under 5% CO_2 humidified atmosphere at 37 °C. After that, the cells were washed with PBS (3 × 3 mL/well) and FDMEM (4 × 100 μL/well) using the plate washer. A solution of Hoechst 33342 (100 μg/mL) was added (50 μL/well) to the cells to a final volume of 150 μL/well. After 10 min of incubation under 5% CO_2 humidified atmosphere at 37 °C, the cells were washed with PBS (9 × 3 mL/well) and kept in FDMEM for imaging. During live cell imaging, samples were kept under 5% CO_2 atmosphere at 37 °C. The distribution of fluorescent signals was captured on a IXM-C with 2 channels, blue for Hoechst 33342 (377/50 nm excitation filter; 477/60 nm emission filter) and red for

1 (620/50 nm excitation filter; 690/50 nm emission filter). Duplicates were performed for each condition.

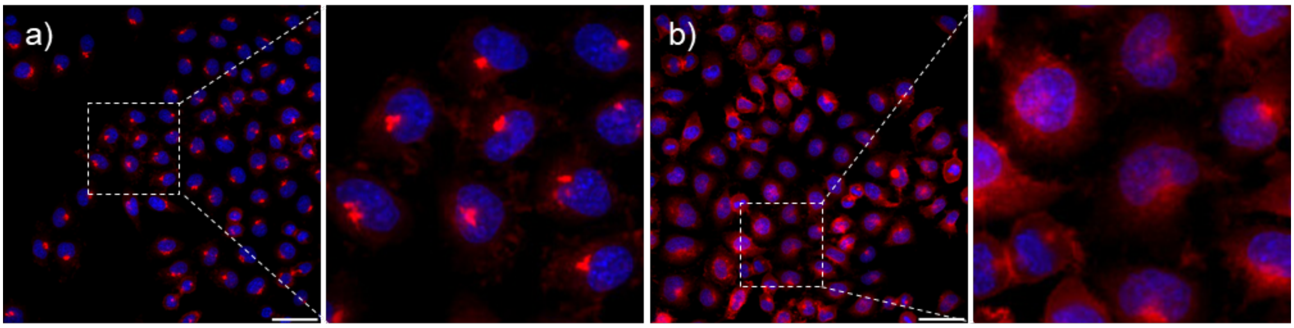

Figure S31. SDCM images (40X WI) showing fluorescence intensity of **1** (500 nM, red) in HK cells a) without BFA or b) after pre-incubating with BFA (10 μM) (blue: Hoechst 33342, nuclei; scale bar 50 μm).

13.3. Vesicle Trafficking

Cells were prepared in a 96 well plate as described in section 4, then medium was removed, and cells were washed with PBS (3 × 3 mL/well) followed by fresh FDMEM serum-free medium (4 × 100 μL/well) using a plate washer (Biotek EL406®), and kept in a 100 μL of the latter medium. A solution of **6** (10 mM, DMSO) and **1** (1 mM, DMSO) were diluted in FDMEM to give a solution at 3x final concentration (60 μM and 1.5 μM for **6** and **1** respectively). 50 μL of **6** was added to the well resulting in a final volume of 150 μL per well (final concentration of 20 μM). The cells were incubated under 5% CO_2 humidified atmosphere at 37 °C for 1 h. Afterward, the cells were washed with PBS (3 x 3 mL/well) and the medium was exchanged with FDMEM keeping a final volume of 100 μL/well, and then, 50 μL of **1** was added to the well resulting in a final volume of 150 μL per well (final concentration of 0.5 μM). The cells were incubated for 10 min under 5% CO_2 humidified atmosphere at 37 °C. The cells were washed with PBS (3 x 3 mL/well) and the medium was exchanged with FDMEM keeping a final volume of 100 μL/well. Hoechst 33342 (100 μg/mL) in PBS (50 μL/well) was added. After 10 min of incubation under 5% CO_2 humidified atmosphere at 37 °C, cells were washed with PBS (3 × 3 mL/well) and fixed by treating with a solution of PFA 3%

(70 µL/well) for 15 min at rt. The excess of PFA was removed by washing with PBS (9 x 3 mL/well). The distribution of fluorescent signals was captured on a IXM-C automated microscope with three channels, blue for Hoechst 33342 (377/50 nm excitation filter; 477/60 nm emission filter), green for **6** (475/34 nm excitation filter; 536/40 nm emission filter) and red for **1** (620/50 nm; emission filter: 690/50 nm). Duplicates were performed for each condition.

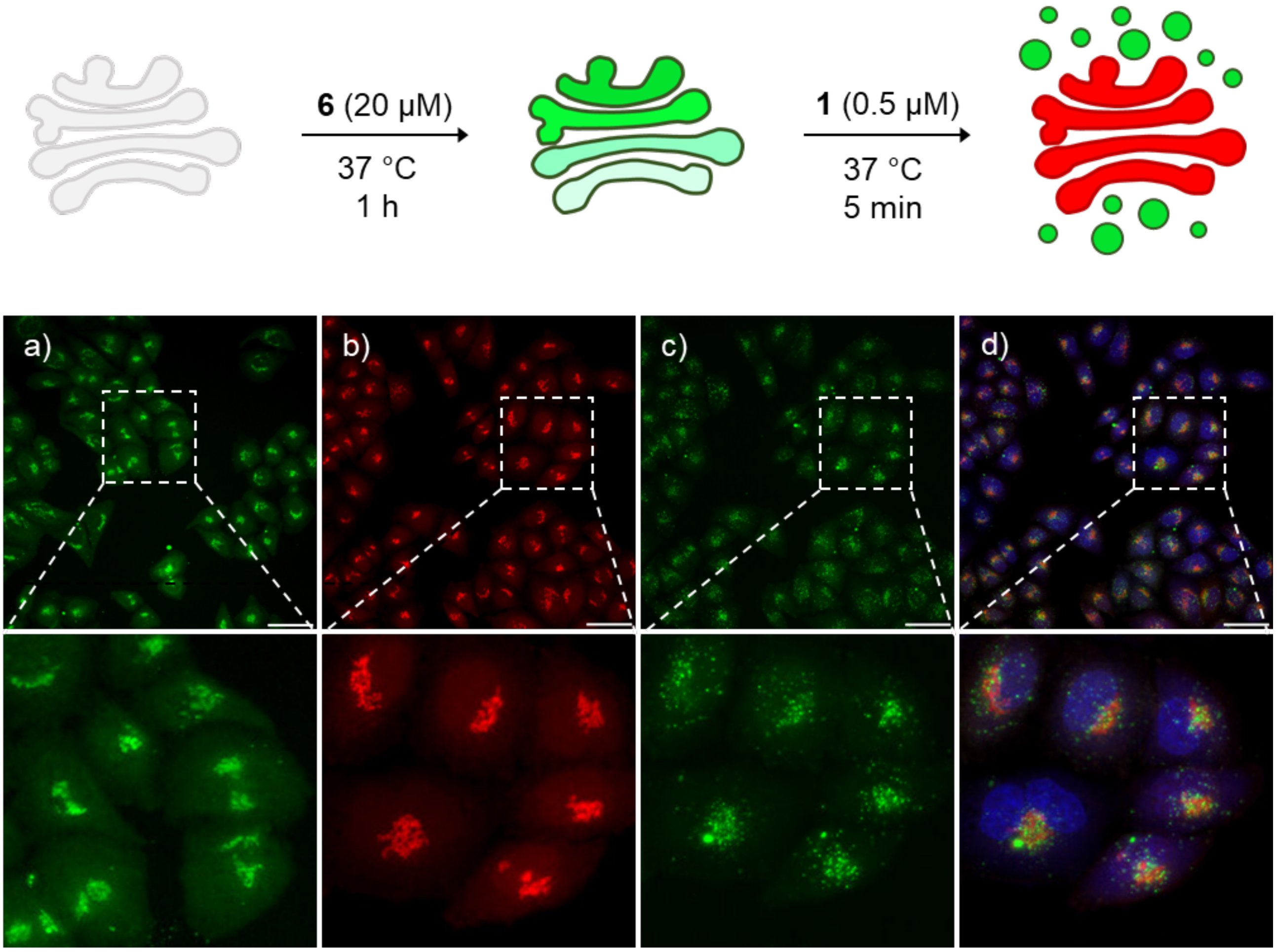

Figure S32. SDCM images (40X WI) of HK cells a) treated with **6** (20 µM, green) for 1 h, rinse with PBS and then treated with b) **1** (500 nM, red) for 5 min causing c-d) the anterograde transport of **6** (vesicles formation, green) (blue: Hoechst 33342, nuclei; scale bar 50 µm).

14. Palmitoylation

Following the procedure described in section 4., HK cells were plated in 6-well sterile plate (10^6 cells/mL, 1 mL/well) and placed in the incubator under 5% CO_2 humidified atmosphere for 24 h at 37 °C. After removing the medium, the cells were washed twice with serum-free FDMEM (2 x 2 mL). The cells were incubated with **1** in FDMEM (10 μM, 1 mL/well) for 10 min at rt. After removing medium, the cells were washed with DPBS (3 x 1 mL/well) and re-suspended in fresh serum-free FDMEM. Lipids were extracted following the procedure reported in ref S8: briefly, the cells were washed with Tris buffer (2 x 1 mL/well, 50 mM Tris-HCl, 150 mM NaCl, 1 mM EDTA, pH = 7.4) and then suspended in the same buffer (1 mL/well) allowing the cells to gently detach. The cell suspension was pelleted by centrifugation at 1000 × g for 5 min and re-suspended in Tris buffer (0.4 mL). Subsequently, chloroform (0.5 mL) and methanol (1.0 mL) were added to the cell suspension. The biphasic solution was mixed thoroughly and left to stand for 10 min at rt. Furthermore, chloroform (0.5 mL) and acidified Tris buffer (0.5 mL, pH = 2.0) were added, and the mixture was mixed thoroughly. After centrifugation (1000 g, 10 min, 4 °C), the lower phase was pooled, washed once with a solution of 10:10:1 methanol/acidified Tris buffer (pH 2.0)/chloroform (2 mL), and dried by using a centrifugal evaporator. The dried lipid extracts were dissolved in 0.1 mL of DMSO and analyzed by RP-HPLC using a Nucleosil 100-7 C2 column (250 x 4.6 mm) with a 10 min run consisted of 1 min at 30% CH_3CN + 0.1% TFA/70% H_2O + 0.1% TFA, followed by 9 minutes of linear gradient from 30% CH_3CN + 0.1% TFA/70% H_2O + 0.1% TFA to 100% CH_3CN + 0.1% TFA (flow rate = 1 mL/ min). The HPLC profiles were obtained by fluorescence detection (excitation at 640 nm, emission at 655 nm).

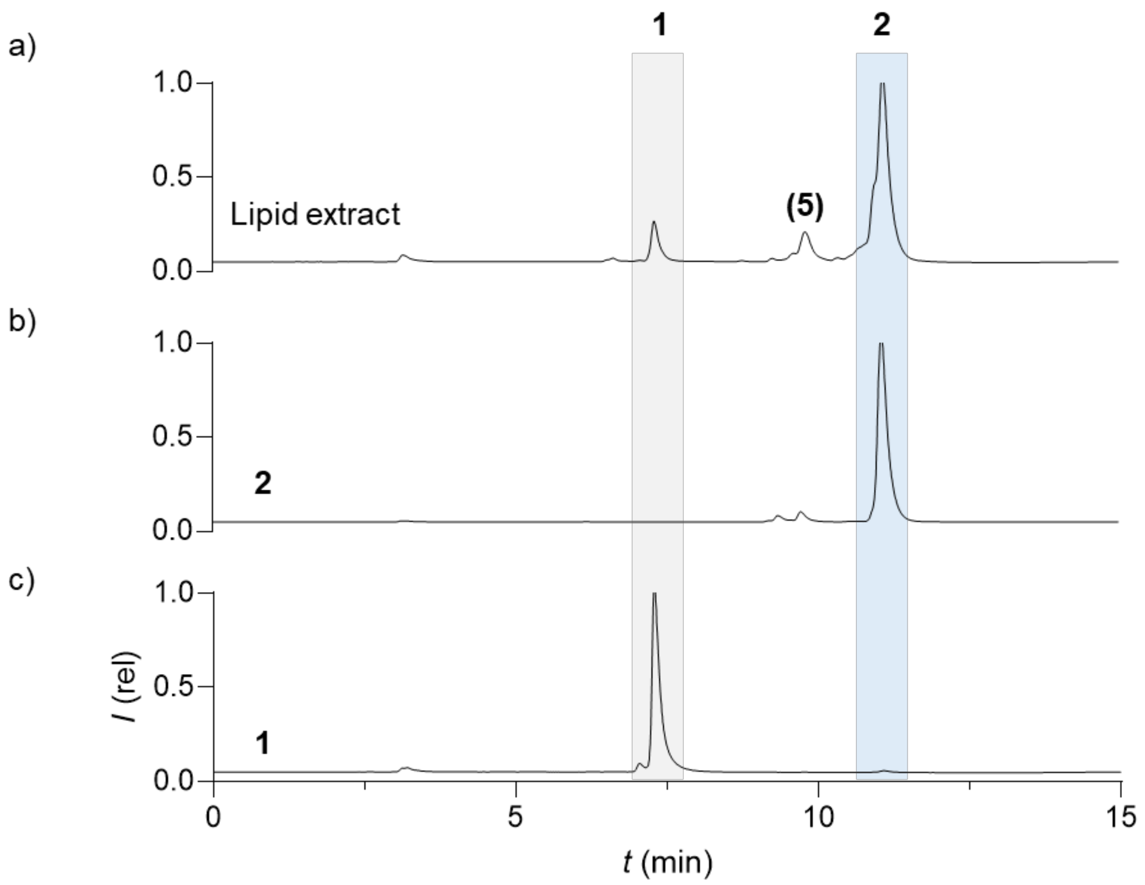

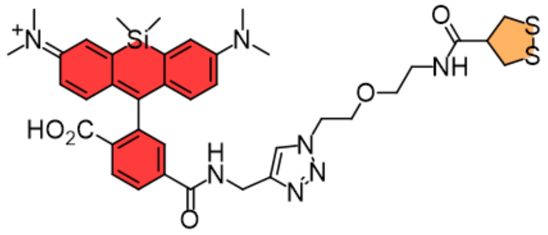

1

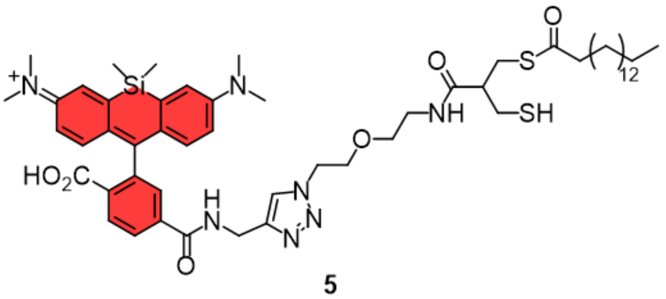

5

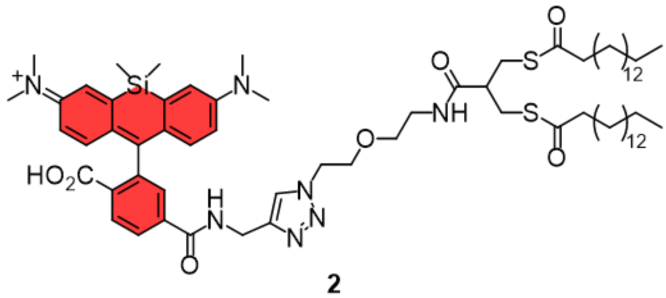

2

Figure S33. Normalized HPLC traces of a) the lipid extract from HK treated with **1** (10 μM, 10 min), b) chemically synthesized **2** and c) **1**. Detection of fluorescence at 655 nm (excitation at 640 nm).

15. Palmitoyl Transferase Inhibitors

15.1. General Procedure

Procedures were adapted from references S1: Briefly, the cells were prepared in a 96-well plate as described in section 4, then medium was removed, and cells were washed with PBS (3 × 3 mL/well) followed by fresh FDMEM (4 × 100 µL/well) using a plate washer (Biotek EL406®), and kept in 100 µL/well of the latter medium. Stock solutions of the inhibitors (3× final concentration), **1** or **6** (10×) and Hoechst 33342 (50 µg/mL) in serum-free FDMEM were prepared freshly in a 96-well V-bottom plate before adding to the cells. The inhibitor solutions from the V-bottom plate were added using an electronic multichannel pipette to the cells (50 µL/well) keeping a final volume of 150 µL/well. Cells were incubated for 1 h for PAT inhibitors and 30 min for endocytosis inhibitors under 5% CO_2 humidified atmosphere at 37 °C. Then, **1** or **6** from the V-bottom plate was added (15 µL/well) to the cells, giving a final volume of 165 µL/well and a final concentration of 1 µM for **1** and 10 µM for **6**. The cells were incubated for an additional 10 minutes for **1** or 60 minutes for **6** under 5% CO_2 humidified atmosphere at 37 °C. After that, the cells were washed with PBS (3 × 3 mL/well) and FDMEM (4 × 100 µL/well) using the plate washer. A solution of Hoechst 33342 from the V-bottom plate was added (50 µL/well) to the cells to a final volume of 150 µL/well. After 10 min of incubation under 5% CO_2 humidified atmosphere at 37 °C, the cells were washed with PBS (9 × 3 mL/well) and kept in FDMEM for imaging. During live cell imaging, samples were kept under 5% CO_2 atmosphere at 37 °C. The distribution of fluorescent signals was captured on a IXM-C automated wide-field fluorescence microscope acquiring 4 images per well using a 10× or 20x objective lens with 3 channels, blue for Hoechst 33342 (377/50 nm excitation filter; 477/60 nm emission filter), green for **6** (475/34 nm excitation filter; 536/40 nm emission filter) and/or red for **1** (620/50 nm excitation filter; 690/50 nm emission filter). Duplicates were performed for each condition.

15.2. Data Analysis

Fluorescence intensities per cell were extracted for each of conditions from the microscope images by the procedure described in section 5.2.

15.3. Influence on Golgi Tracking

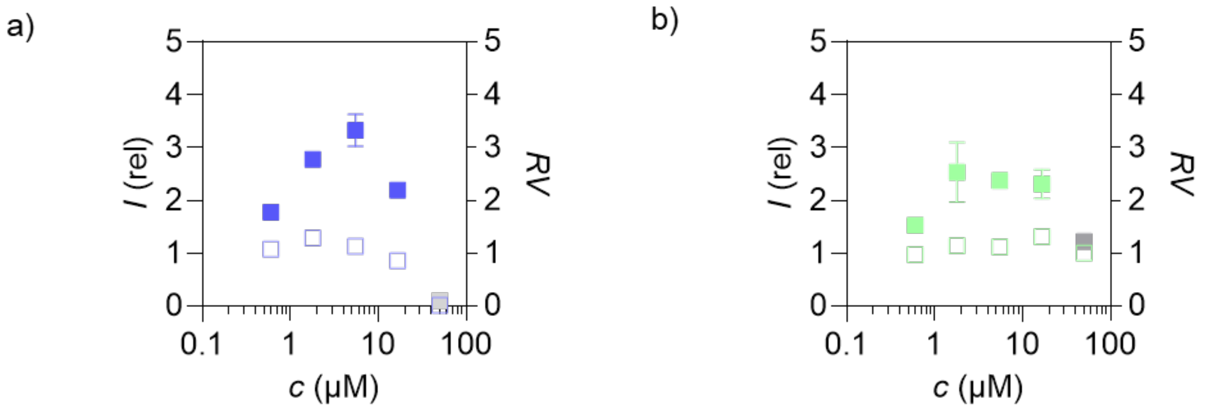

Figure S34. Relative fluorescence intensity I (rel) ± SEM of **1** (1 μM) in HK cells (filled symbols) and relative viability RV ± SEM (empty symbols) as a function of the concentration of a) TM and b) CL.

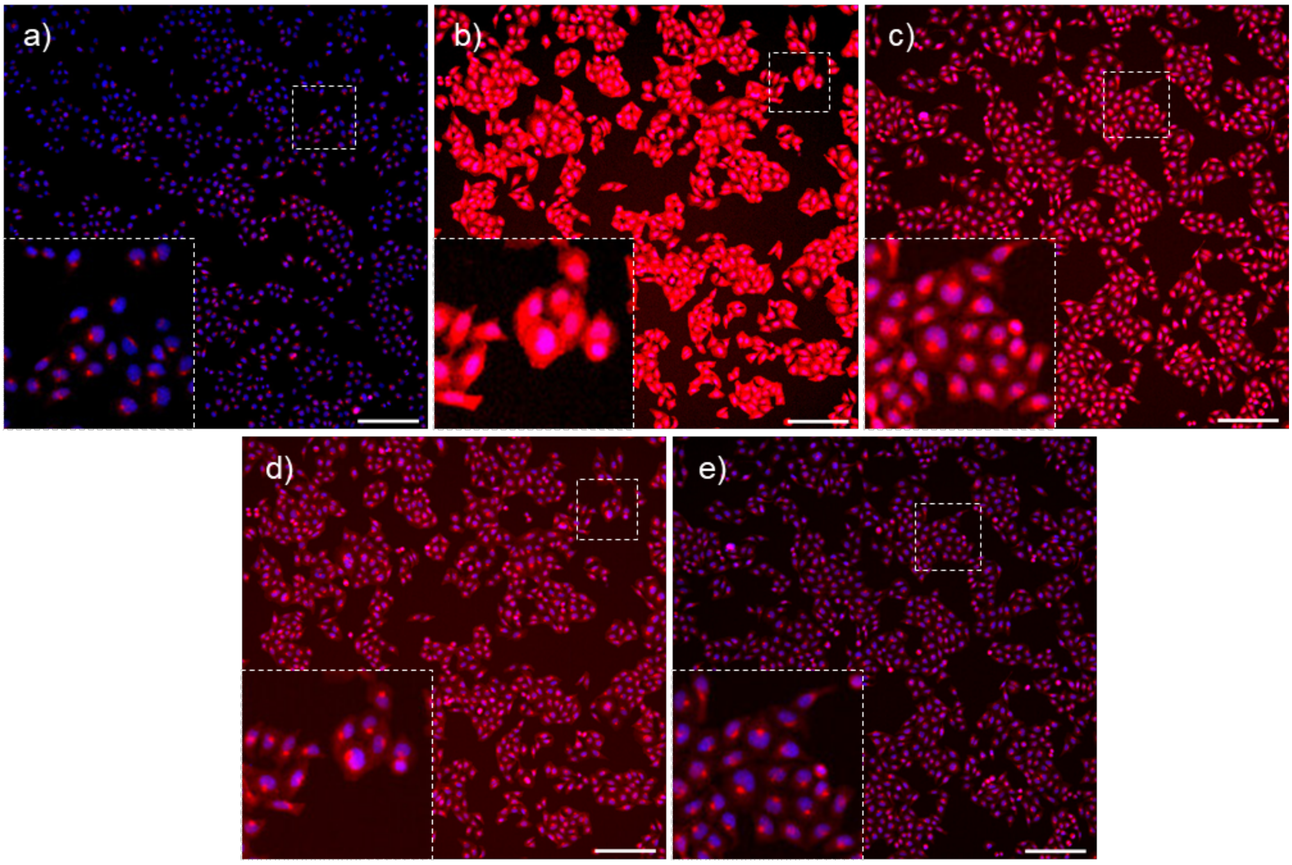

Figure S35. AHCHT images (10X, widefield) showing fluorescence intensity of **1** (1 µM, red) in HK cells a) without any inhibitors, or with b, d) CL (5 µM) or c, e) TM (5 µM) after 1 h of incubation at 37 °C (blue: Hoechst 33342, nuclei; scale bar 200 µm). Intensities of images d) and e) were individually adjusted. Cell count average: 1000 cells/image.

15.4. Endocytosis Inhibitors

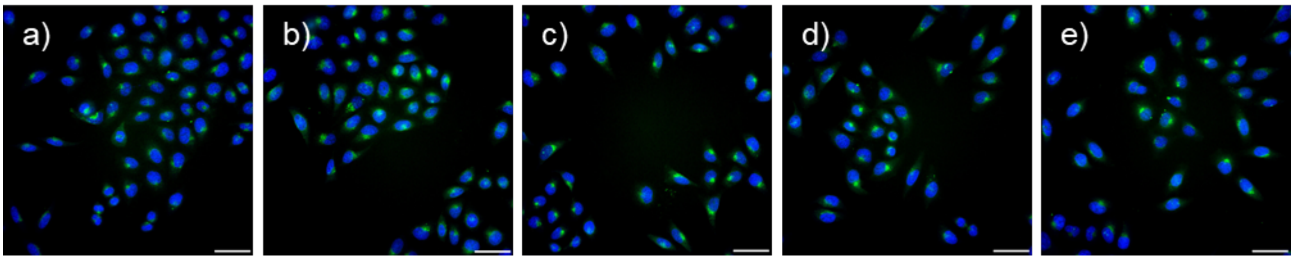

Figure S36. SDCM images (40X WI) showing fluorescence intensity of **6** (10 µM, green) in HK cells a) without any inhibitors, or with b) chlorpromazine (CPZ, 30 µM), c) methyl-β-cyclodextrin (mβCD, 50 µM), d) cytochalasin B (CytoB, 10 µM) and e) wortmannin (Wort, 50 nM) after 1 h of incubation at 37 °C (blue: Hoechst 33342, nuclei; scale bar 50 µm). Cell count average: 50 cells/image.

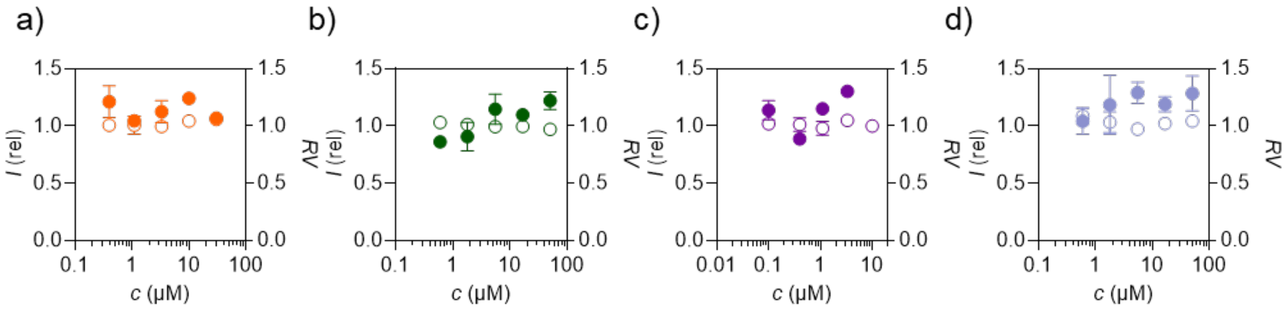

Figure S37. Relative fluorescence intensity *I* (rel) ± SEM of **6** (10 µM) in HK cells (filled symbols) and relative viability *RV* ± SEM (empty symbols) as a function of the concentration of a) CPZ, b) mβCD, c) CytoB and d) Wort.

16. Confocal Laser Scanning Microscopy (CLSM)

Cell preparation. As described in reference S7, HK cells were cultured in 25 cm^2 cell culture flasks with a vent cap and grew in FDMEM (high D-Glucose, without phenol red) containing 10% fetal calf serum (FCS), 1% Penicillin/Streptomycin (PS) and 1% Glutamine. Cell culture flask was kept under 5% CO_2 in a humidified atmosphere at 37 °C. The cells were detached by adding 1 mL of TrypLE Express at 37 °C for 3 min, followed by the addition of 1 mL of FDMEM (same as above). For the microscopy experiment, the cells were seeded at 8×10^4 cells/mL in 35 mm glass bottom dishes from ibidi and kept at 37 °C at 5% CO_2 overnight.

CLSM measurements. The cells were imaged with the Leica Stellaris 8 FALCON, at 20 MHz, with λ_{ex} = 480 nm (white light laser). The cells were washed with PBS (3 x 1 mL) and incubated with **3** (500 nM) for 30 min at 4 °C in HBSS (1 mL). Subsequently, the cells were rinsed with ice-cold FDMEM (x3) and incubated in 1 mL of fresh FDMEM at 37 °C at 5% CO_2 for a further 30 min. The fluorescence was measured between 510 and 540 nm (Laser energy: 5%), or between 600 and 650 nm (laser energy: 90%). Cells in another dish were incubated with **11** (1 µM) for 30 min at 37 °C at 5% CO_2. The fluorescence was measured between 510 and 540 nm (laser energy 90%), or between 600 and 650 nm (laser energy: 90%). All images were acquired without exchanging the incubation medium or additional washing.

To evaluate the co-localization, a solution of **11** (1 µM, 1 mL) in fresh FDMEM was added to cells after the treatment with **3** as described above, and cells were incubated at 37 °C for additional 30 min. The images were acquired without exchanging the incubation medium or additional washing.

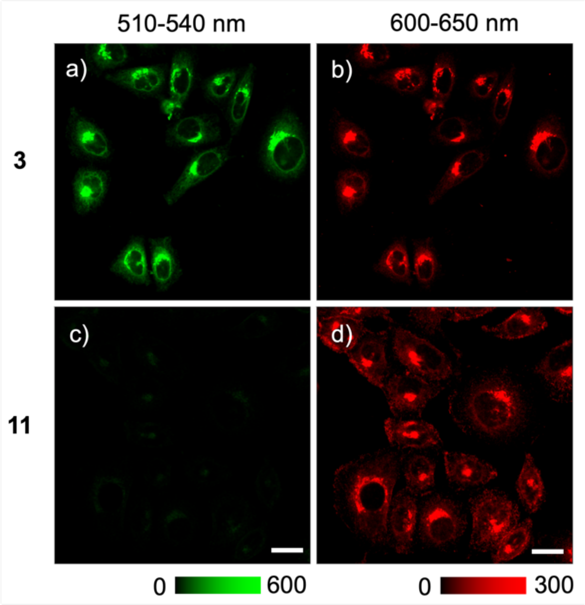

Figure S38. a-b) Confocal microscope images of HK cells after incubation with 0.5 µM of **3**. c-d) Confocal microscope images of HK cells after incubation with 1 µM of **11**. λ_{ex} = 480 nm, emission intensity collected between 510-540 nm (green channel) and 600-650 nm (red channel). Scale bar: 20 µm.

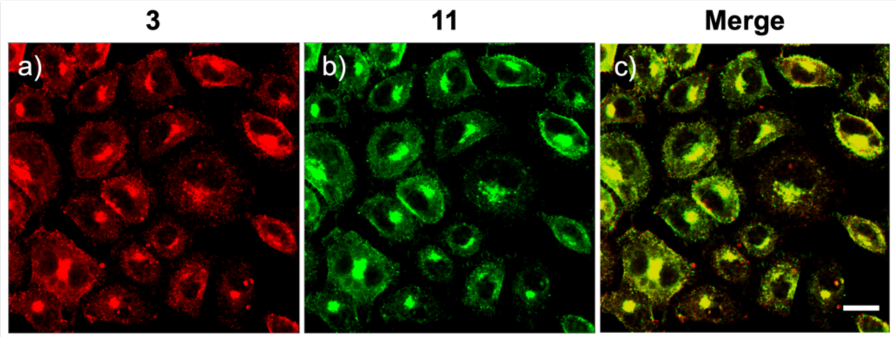

Figure S39. Confocal microscope images of HK cells after co-incubation with (a) **3** (0.5 µM, 60 min), (b) **11** (2 µM, 30 min) and c) the merged image of both. λ_{ex} = 480 nm, emission intensity collected between 510-540 nm (for **3**) and 600-650 nm (for **11**). Scale bar: 20 µm.

17. Fluorescence Lifetime Imaging Microscopy (FLIM)

FLIM measurements. The cells were prepared as previously described and imaged with a Leica Stellaris 8 FALCON, at 20 MHz, with λ_{ex} = 480 nm (for **11**) or λ_{ex} = 600 nm (for **1**) (white light laser). Emission was measured in different channels: **11** (550-650 nm or 600-650 nm), **1** (630-800 nm). The cells were incubated with FDMEM containing the corresponding probe (1 μM of **11** or **1** in 1 mL) for 60 min at 37 °C at 5% CO_2. The images were acquired without exchanging the medium or additional washing. The hypertonic shock was achieved by adding a sucrose medium (1 M, 1 mL) containing the corresponding probe (1 μM for both **11** and **1**) in the dish containing 1 mL of isotonic medium for 30 min. For the analysis, Leica LAS X FLIM/FCS software (4.5.0) was used to fit fluorescent decay data (at least 6 cells per picture) to a triexponential deconvolution model where the intensity weighted average lifetime τ_{av} was extracted. The lifetimes τ_{av} are average of at least 6 cells and the reported values represent the mean ± SD. ROIs were selected by "painting" the GA or plasma membrane for measurements.

Table S2. Response of Golgi Flippers to changes in membrane tension.

Cps[a]	τ_{iso} (ns)[b]		τ_{hyper} (ns)[c]		$\Delta\tau$ (ns)[d]	
	Golgi	PM	Golgi	PM	Golgi	PM
**11**	3.60 ± 0.03	4.24 ± 0.02	3.28 ± 0.04	3.77 ± 0.02	0.31 ± 0.03	0.47 ± 0.03
**TR**	n.d.	5.08 ± 0.06	n.d.	4.54 ± 0.04	n.d.	0.53 ± 0.04

[a]Compound **11** (Golgi Flipper) and **Flipper-TR**. [b]Fluorescence lifetime τ_{av} in Golgi and plasma membrane of isoosmotic HK cells. [c]Fluorescence lifetime τ_{av} in Golgi and plasma membrane of hyperosmotic HK cells. [d]Difference in fluorescence lifetime τ_{av} between isoosmotic and hyperosmotic HK cells. Data displayed as the average ± SD from 6 cells.

Phasor Analysis. The cells were prepared and incubated with **3** (0.5 μM) or **11** (1.0 μM) as described in *CLSM measurements.* FLIM images were acquired in different emission channels: **3** (510-540 nm), **11** (600-650 nm). Subsequently, FLIM images were analysed by using Leica LAS X FLIM/FCS software (4.5.0). For phasor analysis, the lifetimes were estimated by manually selecting the center of photon clouds in phasor plots.[S9]

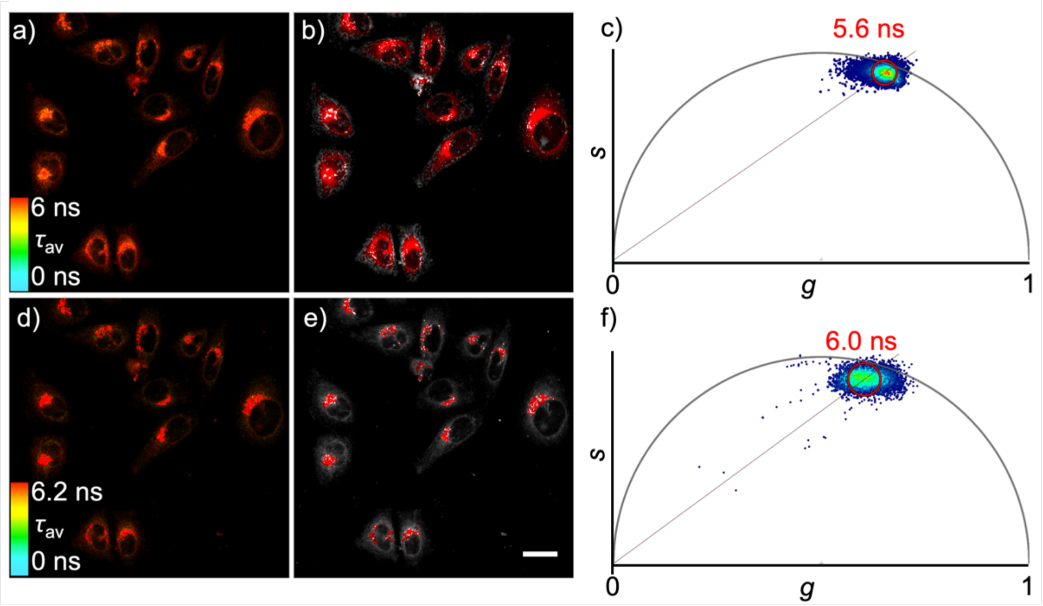

Figure S40. a, d) FLIM images of HK cells after 60 min incubation with 0.5 μM of **3**; b, e) FLIM images reconstructed from its c, f) phasor hemispheres. a-c): λ_{ex} = 480 nm, λ_{em} = 510-540 nm. d-f): λ_{ex} = 480 nm, λ_{em} = 600-650 nm. Scale bar: 20 μm.

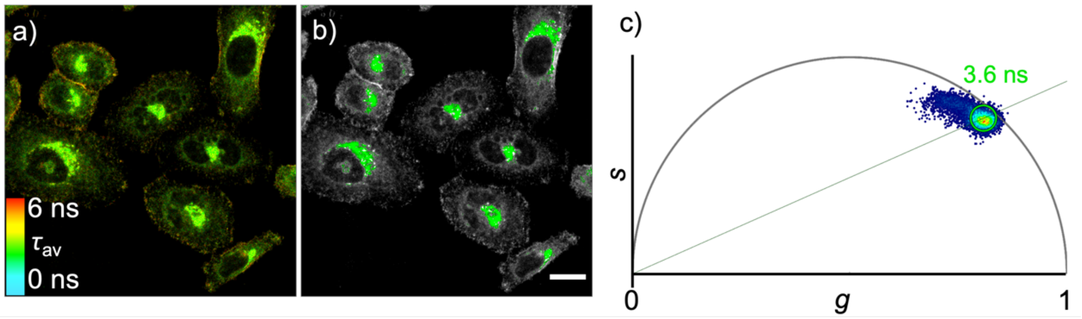

Figure S41. a) FLIM images of HK cells after 30 min incubation with 1.0 µM of **11**. b) FLIM images reconstructed from its c) phasor hemispheres. λ_{ex} = 480 nm, emission intensity collected between 600-650 nm. Scale bar: 20 µm.

Phasor co-localization. The cells were prepared and incubated with **3** (0.5 µM) and **11** (1.0 µM) as described in *CLSM measurements* and imaged with the Leica Stellaris 8 FALCON, at 20 MHz, with λ_{ex} = 480 nm (white light laser). The fluorescence was measured between 510 and 540 nm for **3** and between 600-650 nm for **11**. FLIM images were analysed by using Leica LAS X FLIM/FCS software (4.5.0). The lifetimes were estimated by manually selecting the center of photon clouds in phasor plots as described in ref S9. The probe co-localization was evidenced by comparing the mapped images originating from differently positioned Phasor clouds of **3** and **11**.

18. Live-Cell STED Imaging

Live-cell imaging was carried out on glass-bottom dishes (3.5 cm diameter, No. 1.5 glass; Cellvis) previously coated with fibronectin at a concentration of 1 µg/mL for 1 h at 37 °C and 5% CO_2. Microscopy dishes were washed twice with PBS. The day after seeding, cells were labelled for 1 h using Halo substrates (JF_{571}-CA) at a concentration of 1 µM.[S10] To remove the excess of dye, cells were washed three times with warm growth medium after the staining and incubated for 1 h at 37 °C and 5% CO_2. After labelling with Halo substrates, cells were further labelled with **1** at a concentration of 0.5 µM for 10 minutes in PBS at 37 °C and 5% CO_2. To remove the excess of dye, cells were washed with warm PBS (x3) after the staining and directly imaged at 37 °C. Live-cell

imaging was performed using FDMEM supplemented with 10% FBS, 20 mM HEPES and 1x GlutaMAX. Imaging was carried out on a commercial expert line Abberior STED microscope equipped with 485 nm, 561 nm, and 645 nm excitation lasers and an Olympus Objective UPlanSApo 100x, NA 1.40, WD 0.13. The 775 nm depletion laser served to deplete dyes in dual-color STED experiments. Dual-color images were acquired sequentially line by line. The detection was carried out using avalanche photodiodes with detection windows set to 571-630 nm and 650-756 nm. The pixel size was set to 30 nm for STED imaging. Live-cell STED experiments were carried out at 37 °C.[S11]

19. Supporting References

(S1) Saidjalolov, S.; Coelho, F.; Mercier, V.; Moreau, D.; Matile, S. Inclusive Pattern Generation Protocols to Decode Thiol-Mediated Uptake. *ACS Cent. Sci.* **2024**, *10*, 1033–1043.

(S2) Gasparini, G.; Sargsyan, G.; Bang, E.-K.; Sakai, N.; Matile, S. Ring Tension Applied to Thiol-Mediated Cellular Uptake. *Angew. Chem. Int. Ed.* **2015**, *54*, 7328–7331.

(S3) Fabre, B.; Pícha, J.; Vaněk, V.; Selicharová, I.; Chrudinová, M.; Collinsová, M.; Žáková, L.; Buděšínský, M.; Jiráček, J. Synthesis and Evaluation of a Library of Trifunctional Scaffold-Derived Compounds as Modulators of the Insulin Receptor. *ACS Comb. Sci.* **2016**, *18*, 710–722.

(S4) Abegg, D.; Gasparini, G.; Hoch, D. G.; Shuster, A.; Bartolami, E.; Matile, S.; Adibekian, A. Strained Cyclic Disulfides Enable Cellular Uptake by Reacting with the Transferrin Receptor. *J. Am. Chem. Soc.* **2017**, *139*, 231–238.

(S5) Lin, P.-C.; Ueng, S.-H.; Tseng, M.-C.; Ko, J.-L.; Huang, K.-T.; Yu, S.-C.; Adak, A. K.; Chen, Y.-J.; Lin, C.-C. Site-Specific Protein Modification through CuI-Catalyzed 1,2,3-Triazole Formation and Its Implementation in Protein Microarray Fabrication. *Angew. Chem. Int. Ed.* **2006**, *45*, 4286–4290.

(S6) Dal Molin, M.; Verolet, Q.; Colom, A.; Letrun, R.; Derivery, E.; Gonzalez-Gaitan, M.;

Vauthey, E.; Roux, A.; Sakai, N.; Matile, S. Fluorescent Flippers for Mechanosensitive Membrane Probes. *J. Am. Chem. Soc.* **2015**, *137*, 568–571.

(S7) Chen, X.-X.; Gomila, R. M.; García-Arcos, J. M.; Vonesch, M.; Gonzalez-Sanchis, N.; Roux, A.; Frontera, A.; Sakai, N.; Matile, S. Fluorogenic In Situ Thioacetalization: Expanding the Chemical Space of Fluorescent Probes, Including Unorthodox, Bifurcated, and Mechanosensitive Chalcogen Bonds. *JACS Au* **2023**, *3*, 2557–2565.

(S8) Sawada, S.; Yoshikawa, M.; Tsutsui, K.; Miyazaki, T.; Kano, K.; Mishiro-Sato, E.; Tsukiji, S. Palmitoylation-Dependent Small-Molecule Fluorescent Probes for Live-Cell Golgi Imaging. *ACS Chem. Biol.* **2023**, *18*, 1047–1053.

(S9) Ranjit, S.; Malacrida, L.; Jameson, D. M.; Gratton, E. Fit-Free Analysis of Fluorescence Lifetime Imaging Data Using the Phasor Approach. *Nat. Protoc.* **2018**, *13*, 1979–2004.

(S10) Grimm, J. B.; Tkachuk, A. N.; Xie, L.; Choi, H.; Mohar, B.; Falco, N.; Schaefer, K.; Patel, R.; Zheng, Q.; Liu, Z.; Lippincott-Schwartz, J.; Brown, T. A.; Lavis, L. D. A General Method to Optimize and Functionalize Red-Shifted Rhodamine Dyes. *Nat. Methods* **2020**, *17*, 815–821.

(S11) Wong-Dilworth, L.; Rodilla-Ramirez, C.; Fox, E.; Restel, S. D.; Stockhammer, A.; Adarska, P.; Bottanelli, F. STED Imaging of Endogenously Tagged ARF GTPases Reveals Their Distinct Nanoscale Localizations. *J. Cell Biol.* **2023**, *222*, e202205107.

The original data can be found at: https://doi.org/10.5281/zenodo.12750855

20. NMR Spectra

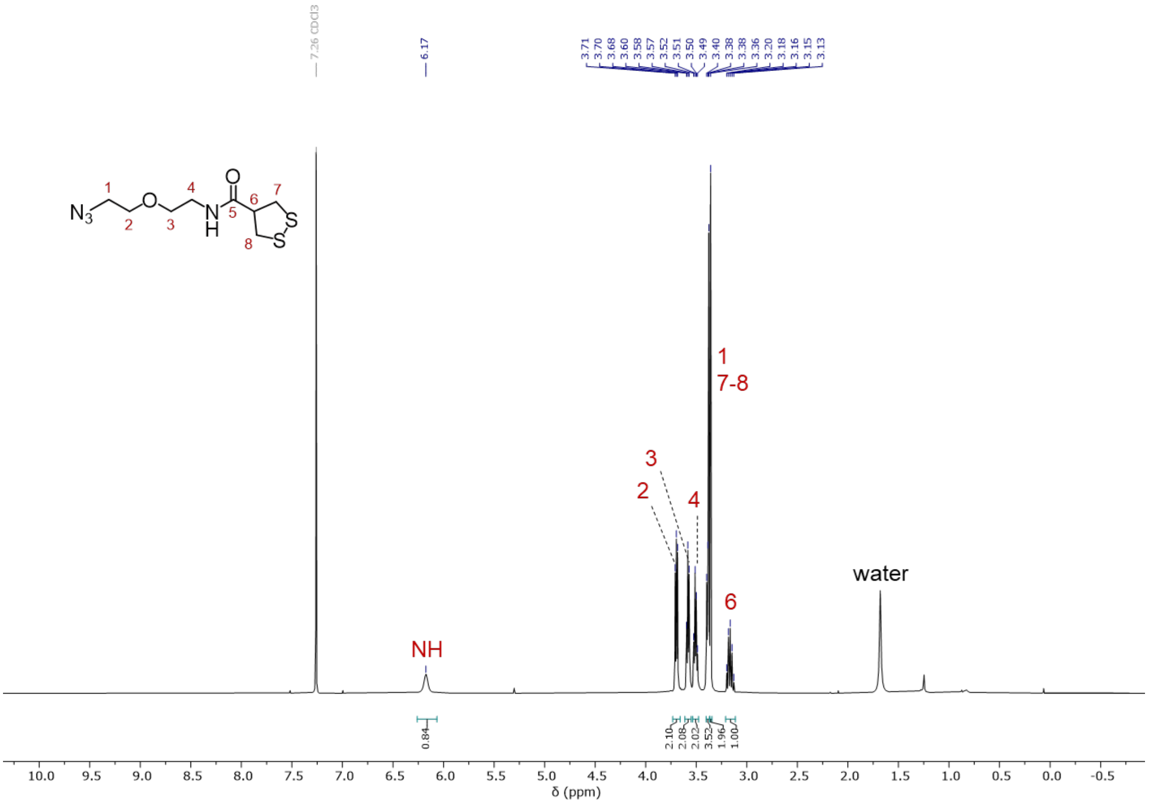

Figure S42. 400 MHz ^{1}H NMR spectrum of compound **14** in $CDCl_3$.

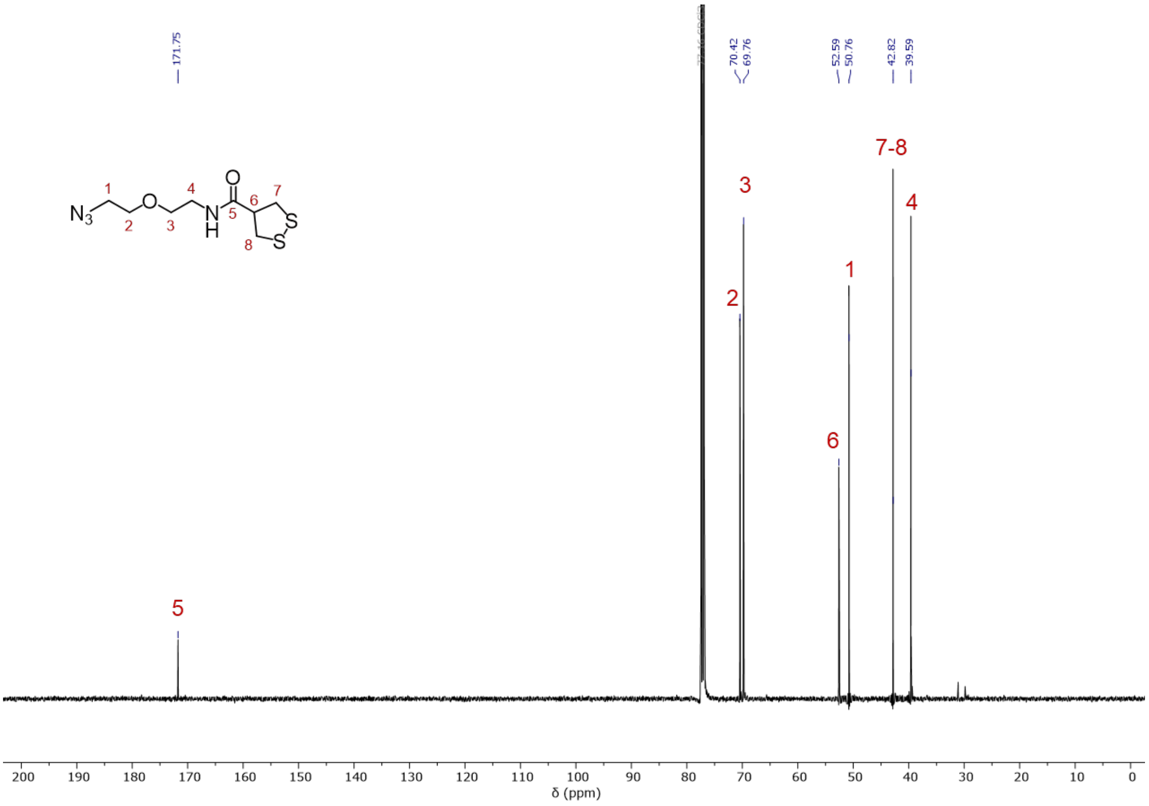

Figure S43. 101 MHz ^{13}C NMR spectrum of compound **14** in $CDCl_3$.

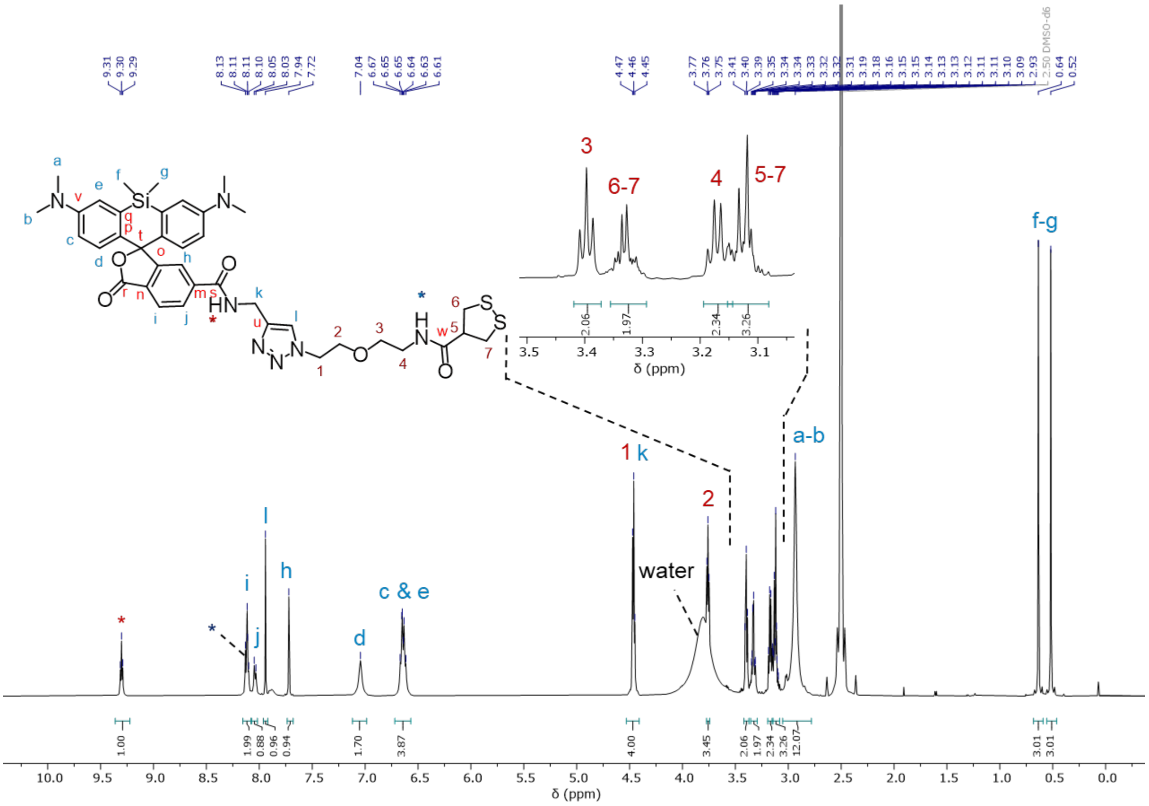

Figure S44. 500 MHz ^{1}H NMR spectrum of compound **1** in DMSO-d_6.

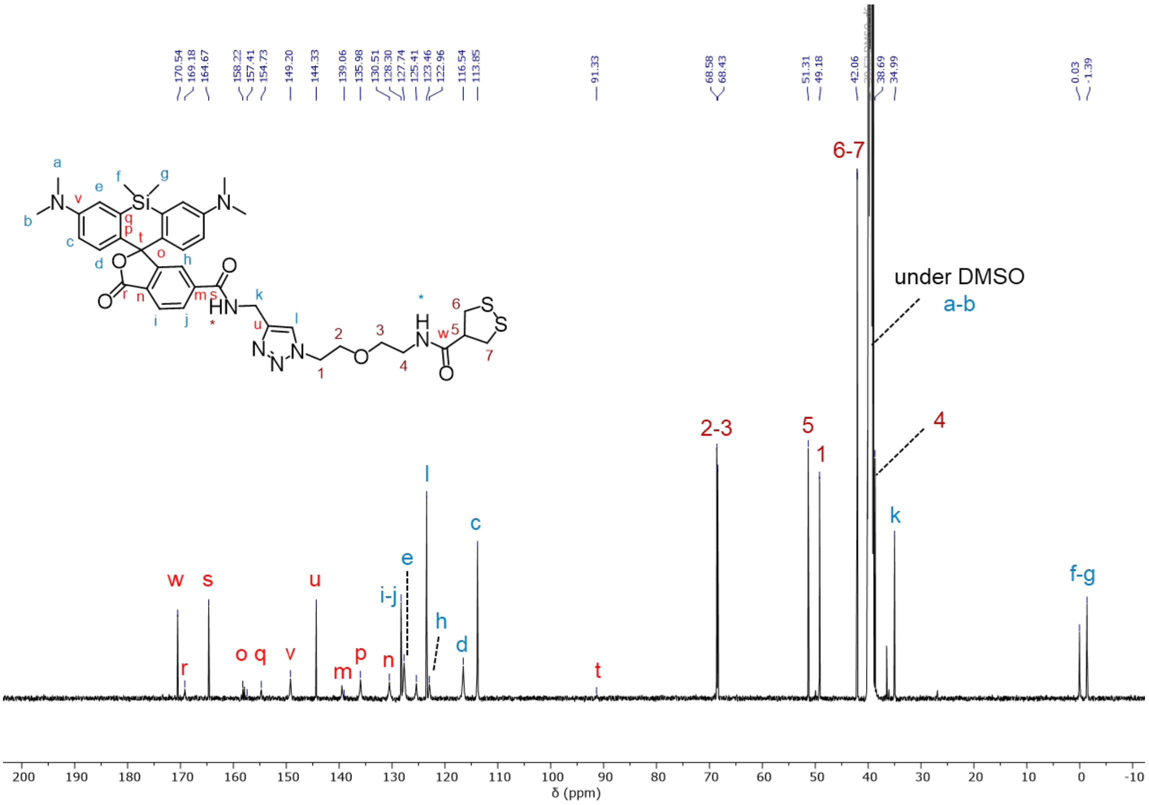

Figure S45. 126 MHz ^{13}C NMR spectrum of compound **1** in DMSO-d_6.

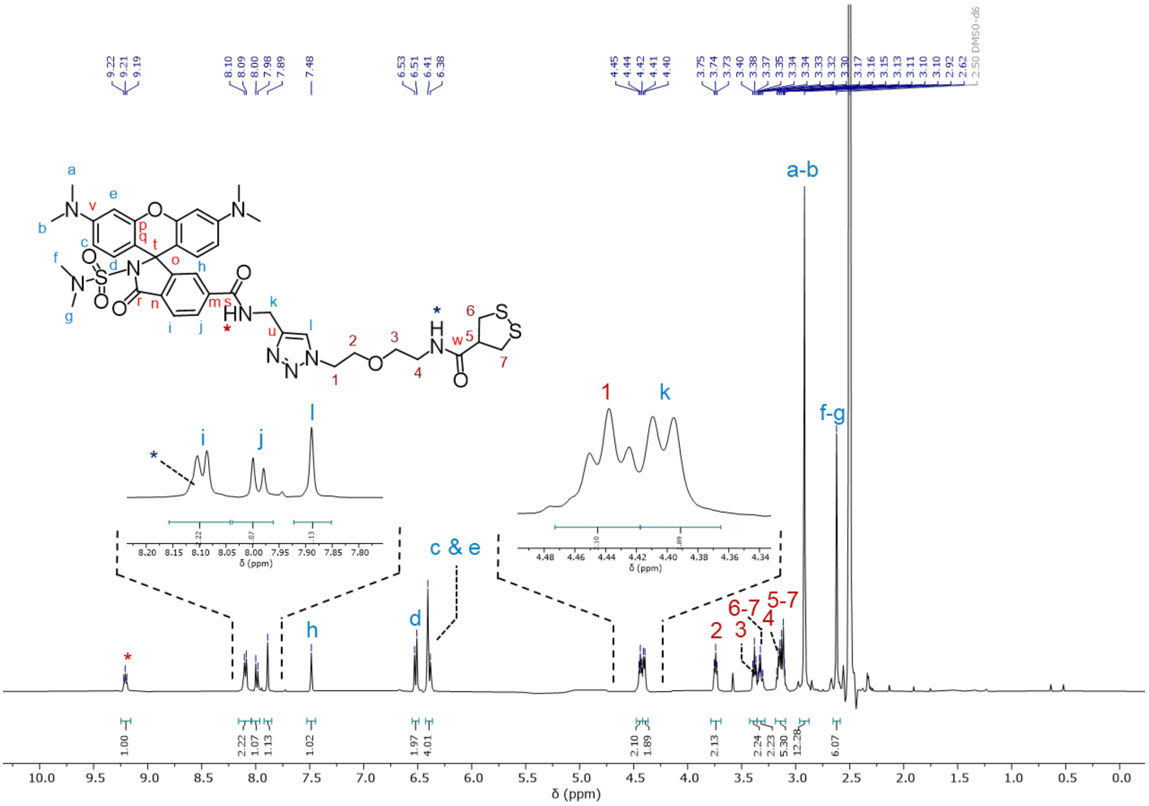

Figure S46. 400 MHz ^{1}H NMR spectrum of compound **7** in DMSO-d_6.

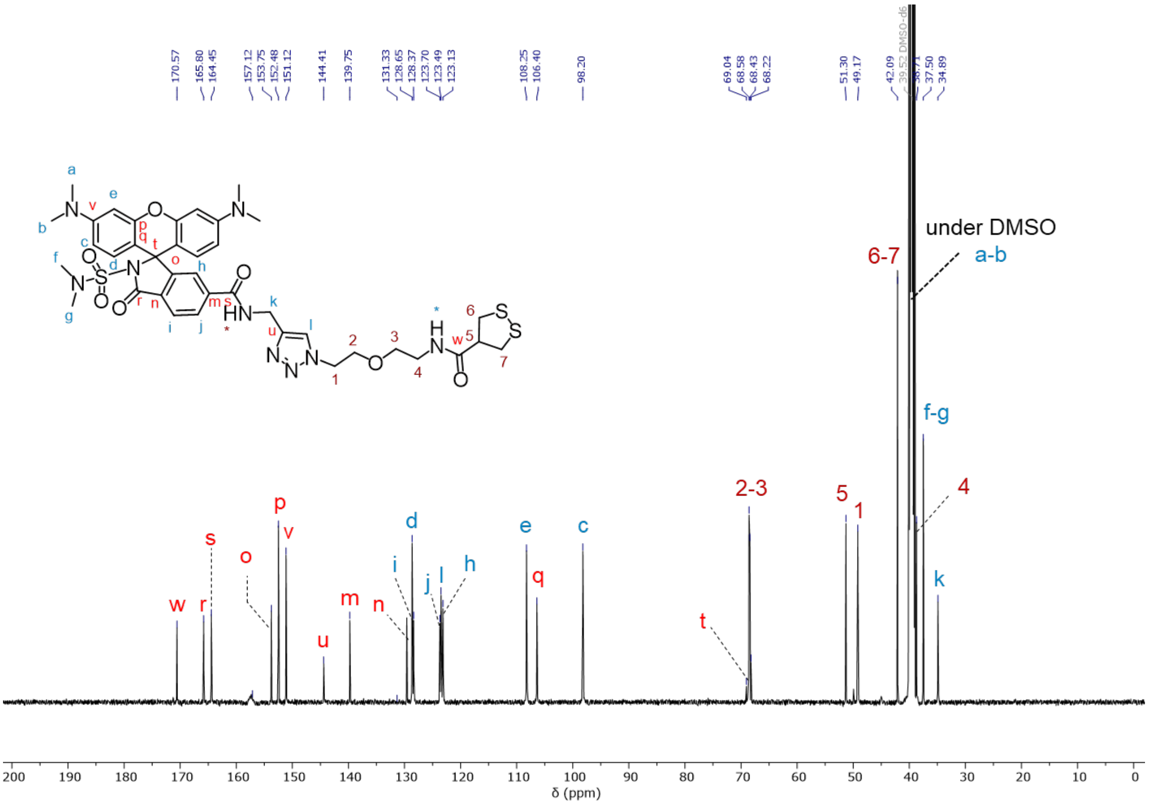

Figure S47. 126 MHz ^{13}C NMR spectrum of compound **7** in DMSO-d_6.

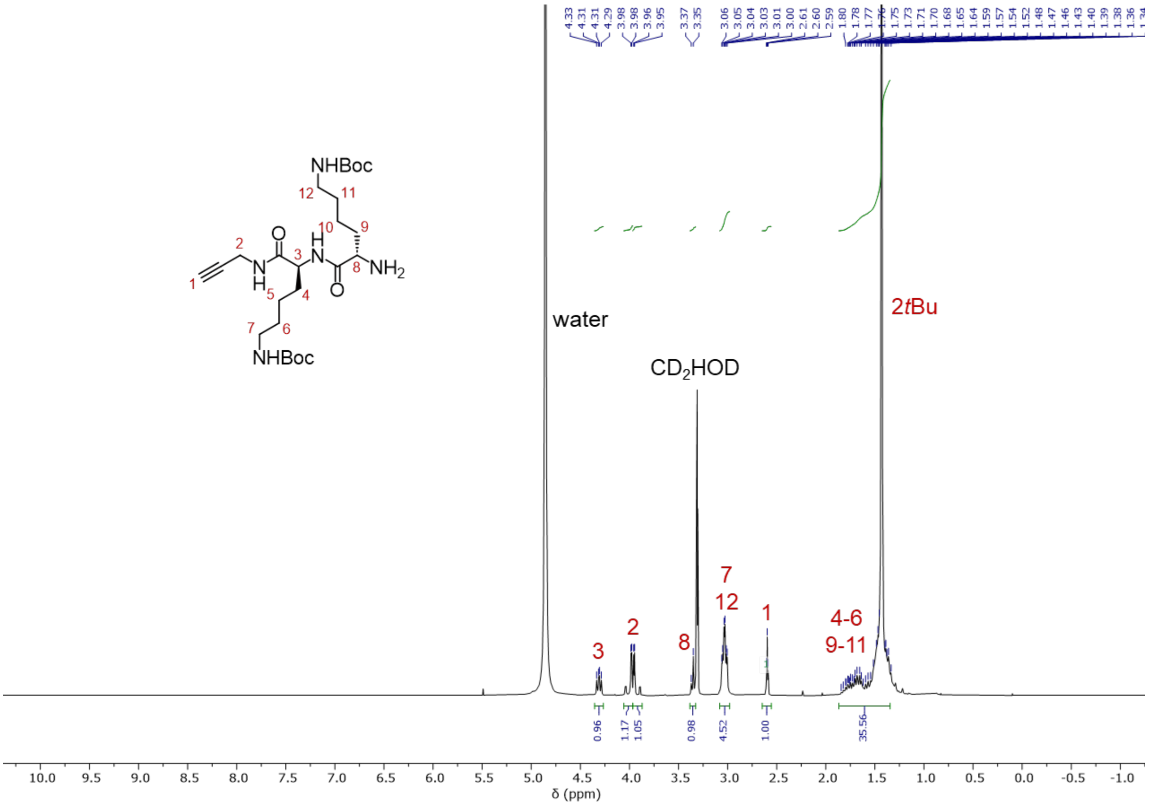

Figure S48. 300 MHz ^{1}H NMR spectrum of compound **22** in CD_3OD.

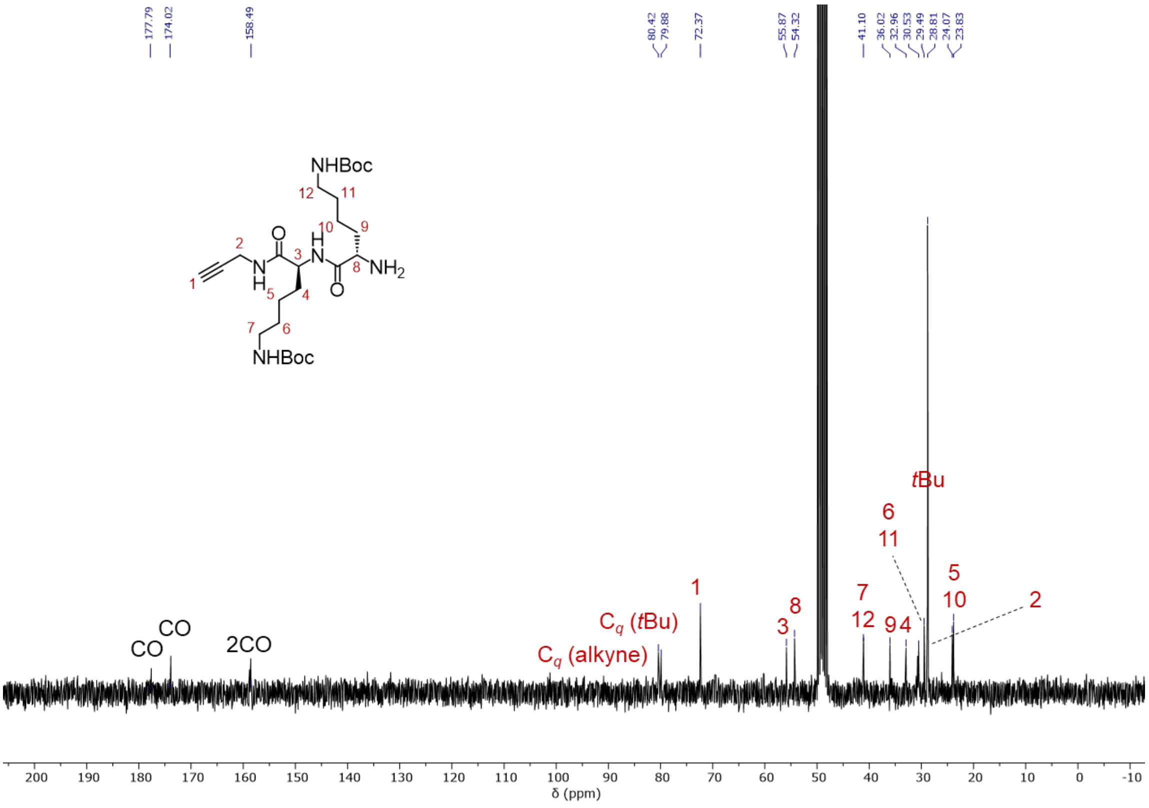

Figure S49. 75 MHz ^{13}C NMR spectrum of compound **22** in CD_3OD.

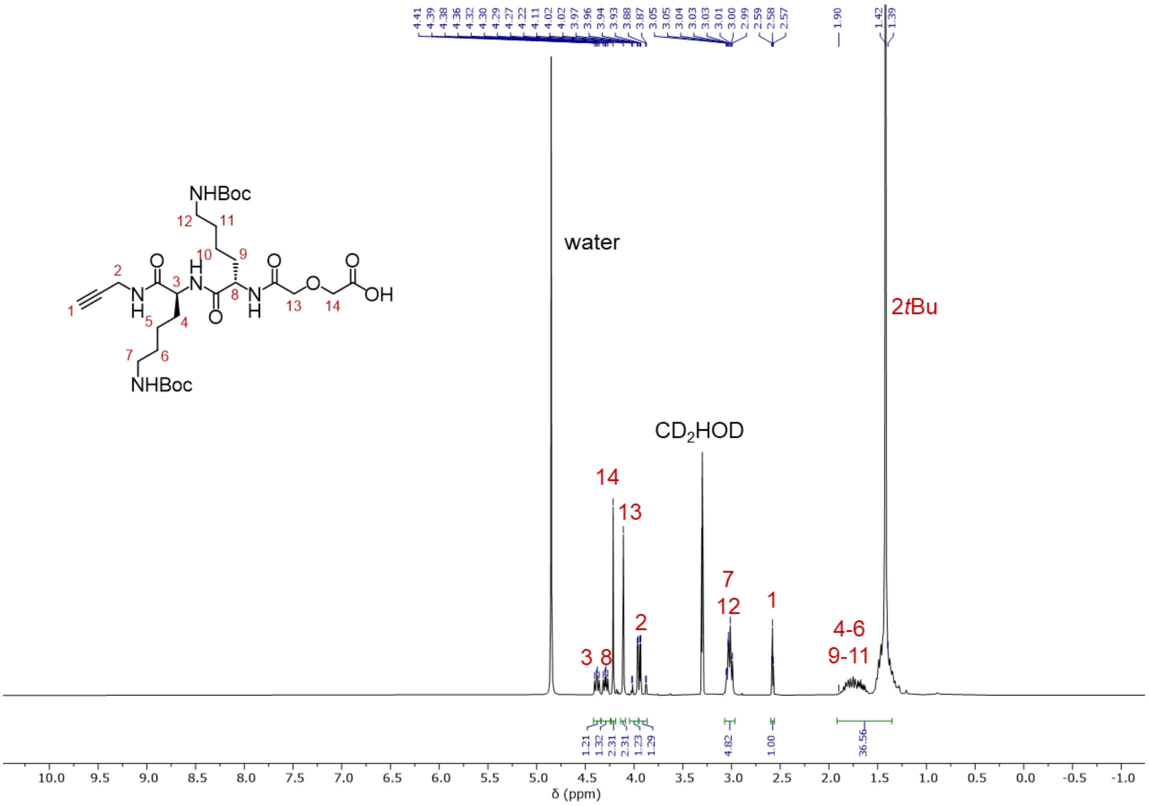

Figure S50. 500 MHz ^{1}H NMRspectrum of compound **24** in CD_3OD.

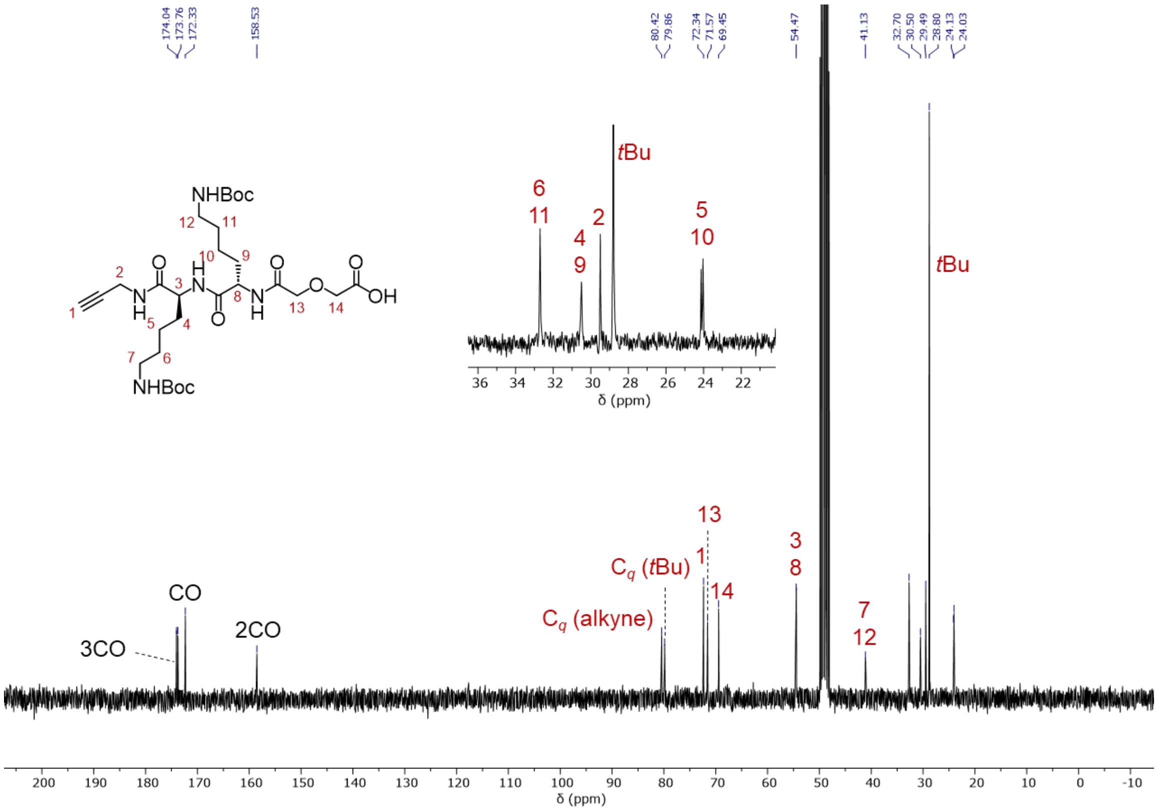

Figure S51. 126 MHz ^{13}C NMR spectrum of compound **24** in DMSO-d_6.

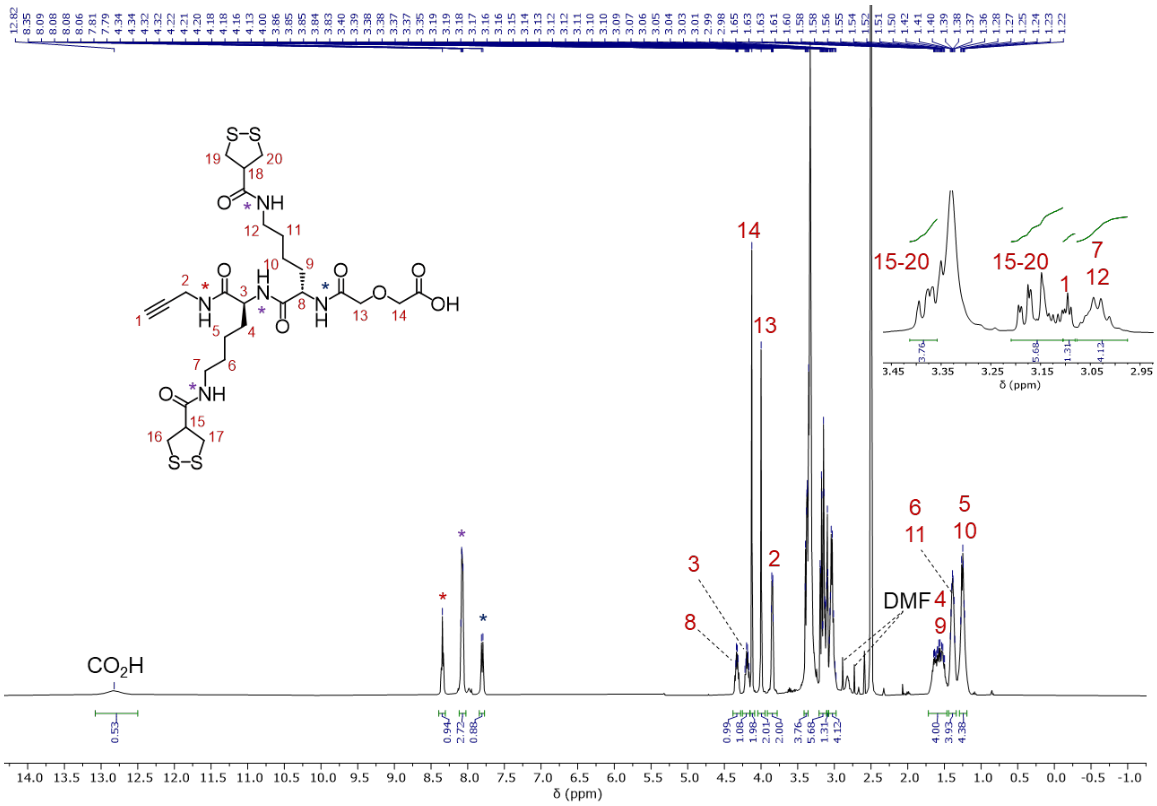

Figure S52. 400 MHz ^{1}H NMR spectrum of compound **26** in DMSO-d_6.

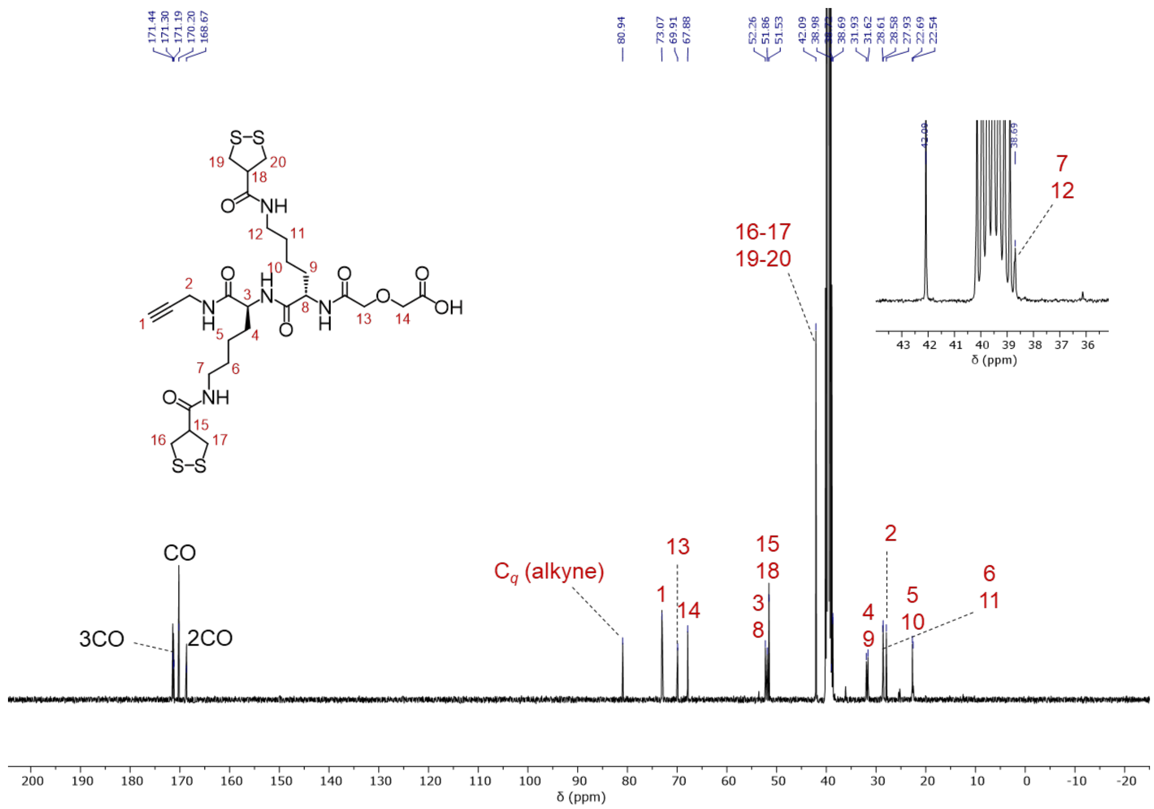

Figure S53. 101 MHz ^{13}C NMR spectrum of compound **26** in DMSO-d_6.

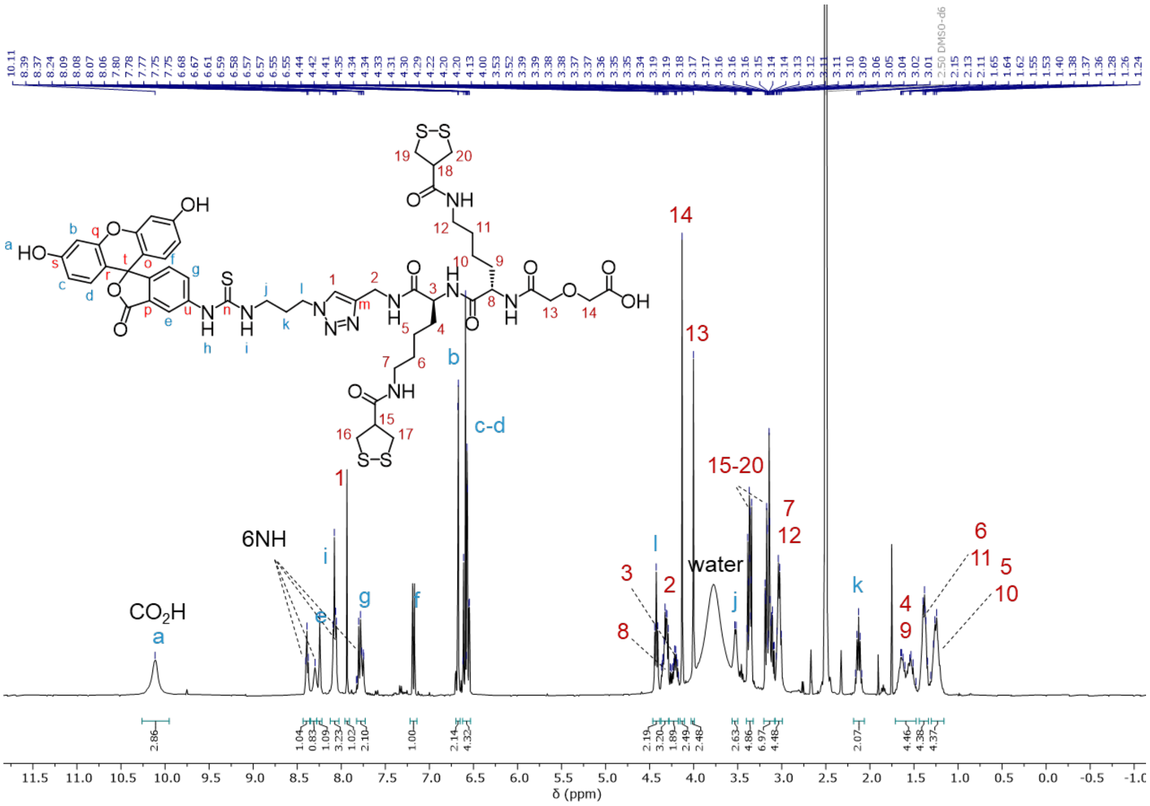

Figure S54. 400 MHz ^{1}H NMR spectrum of compound **8** in DMSO-d_6.

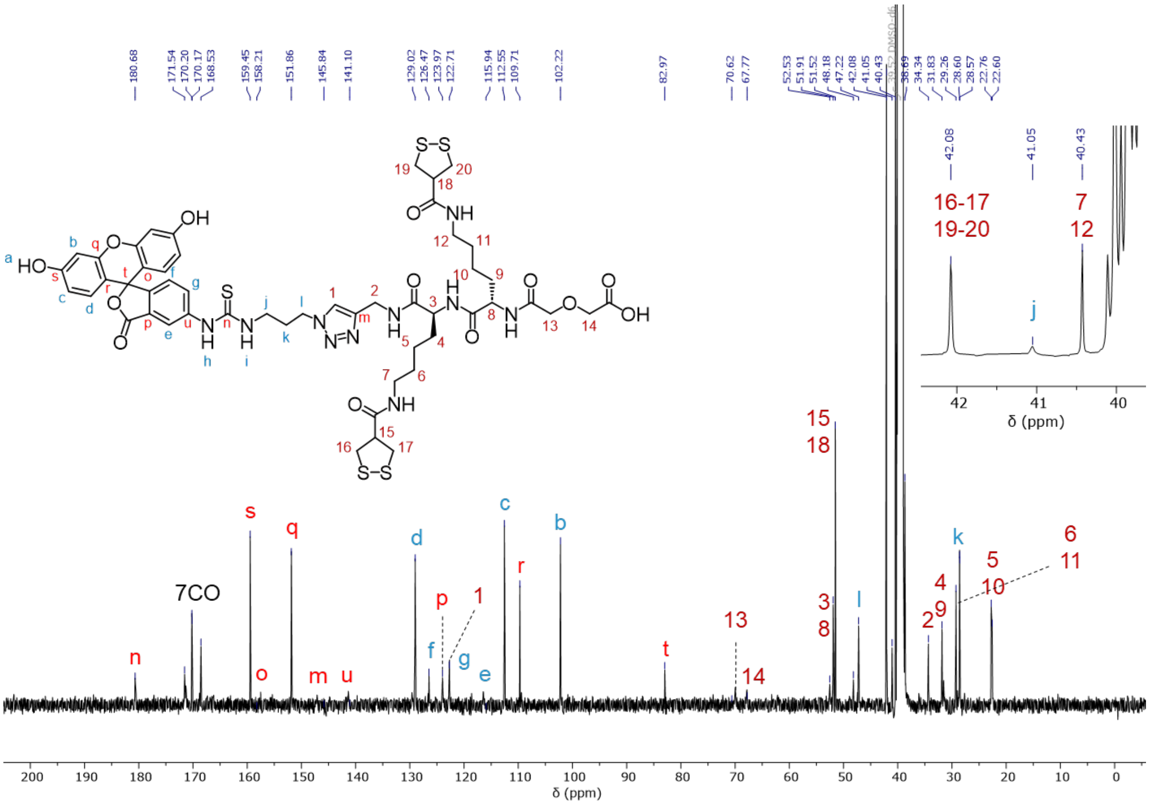

Figure S55. 126 MHz ^{13}C NMR spectrum of compound **8** in DMSO-d_6.

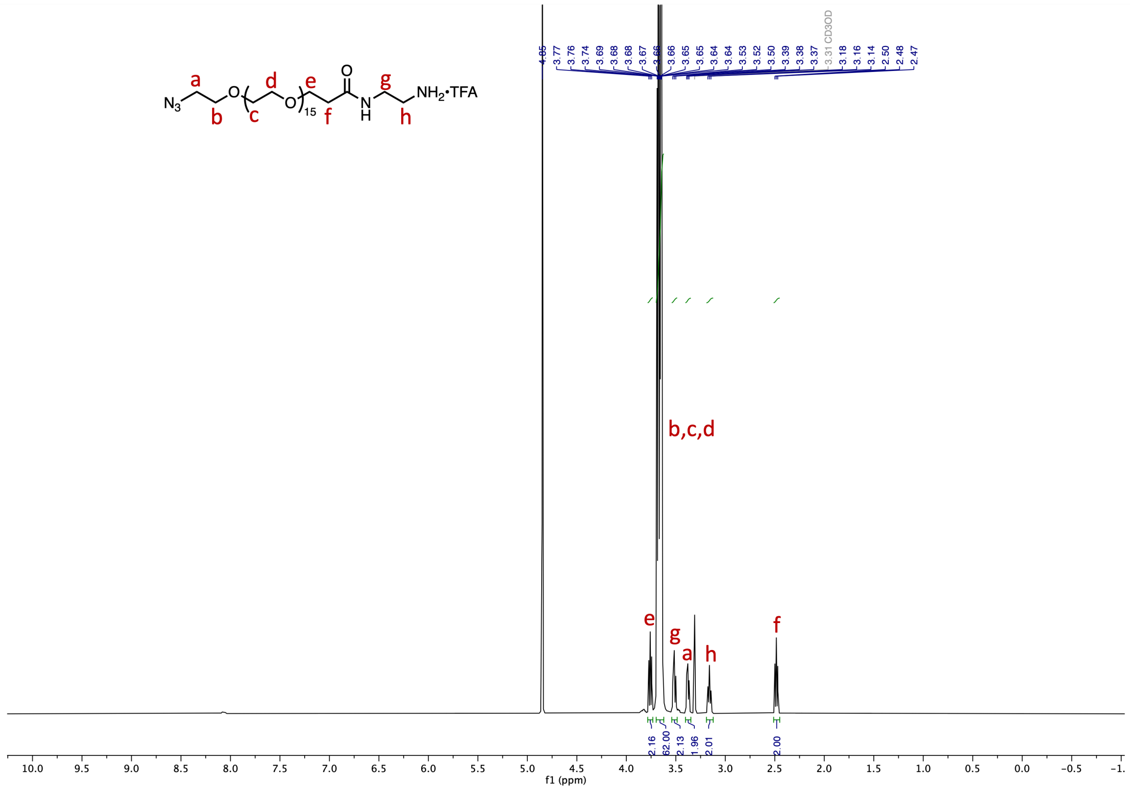

Figure S56. 400 MHz ^{1}H NMR spectrum of compound **30** in CD_3OD.

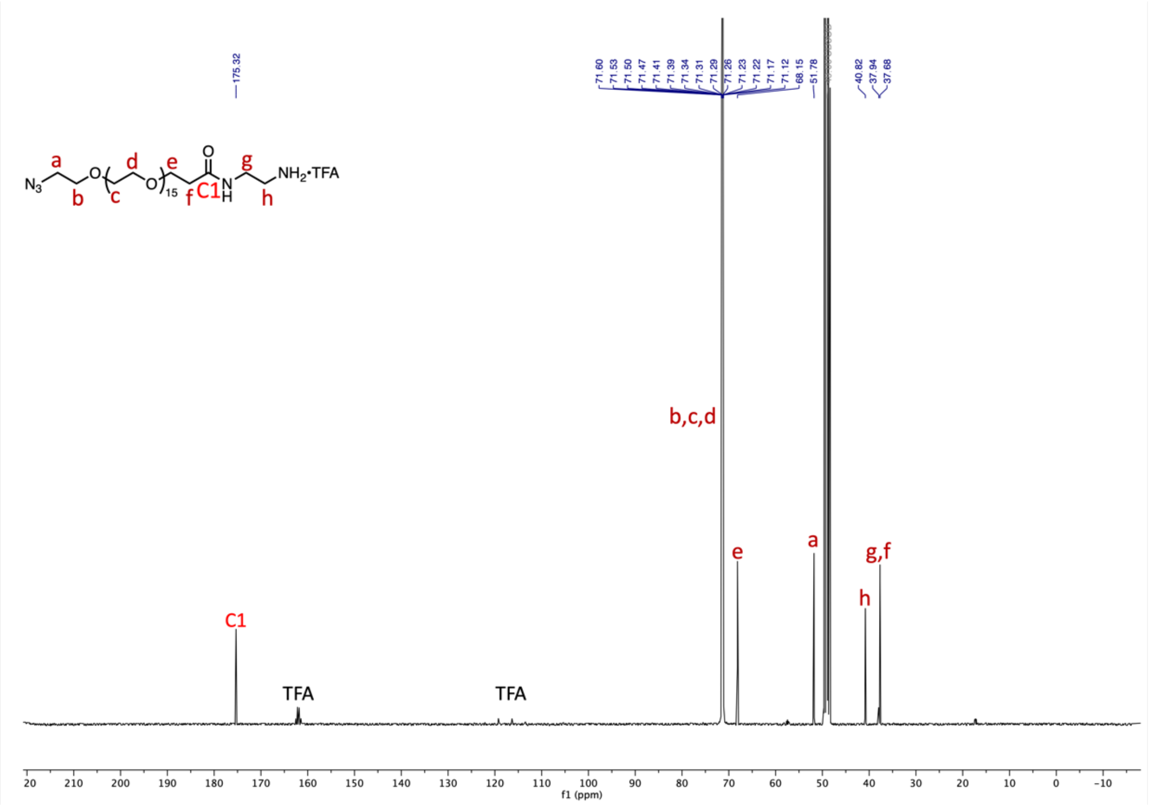

Figure S57. 101 MHz ^{13}C NMR spectrum of compound **30** in CD_3OD.

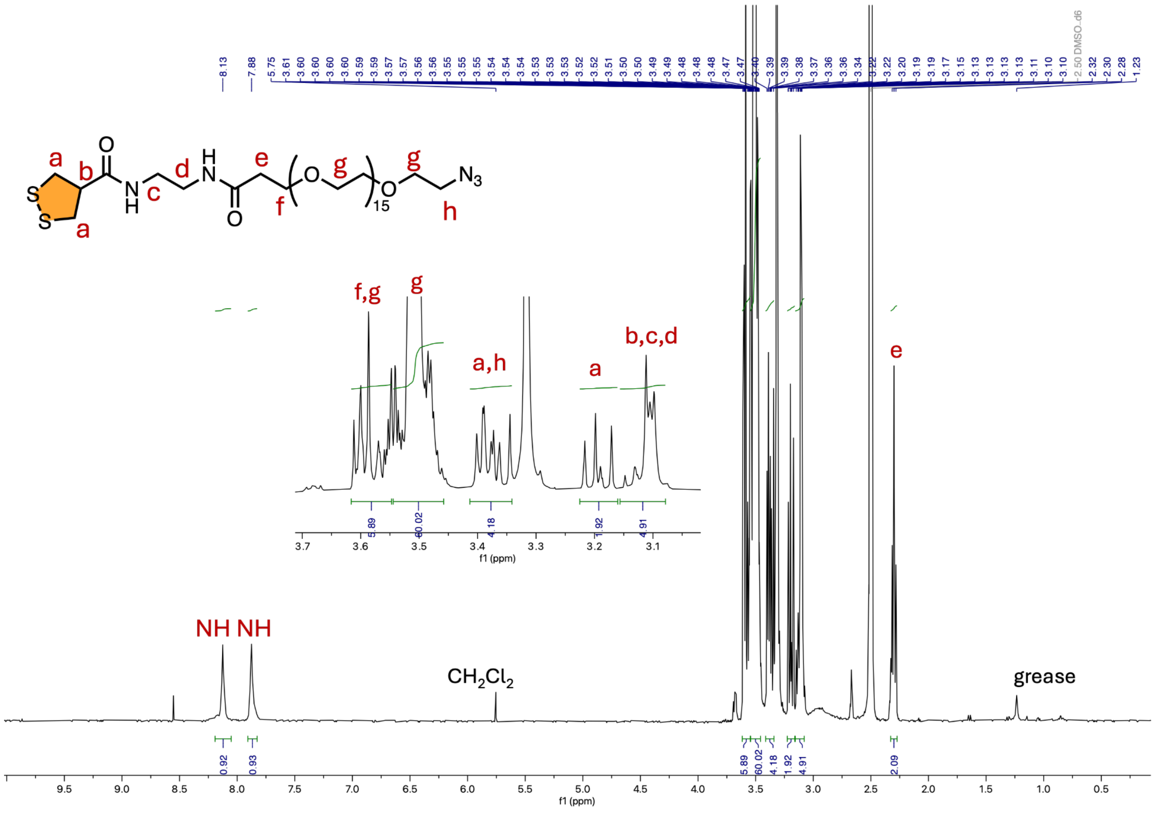

Figure S58. 400 MHz ^{1}H NMR spectrum of compound **31** in DMSO-d_6.

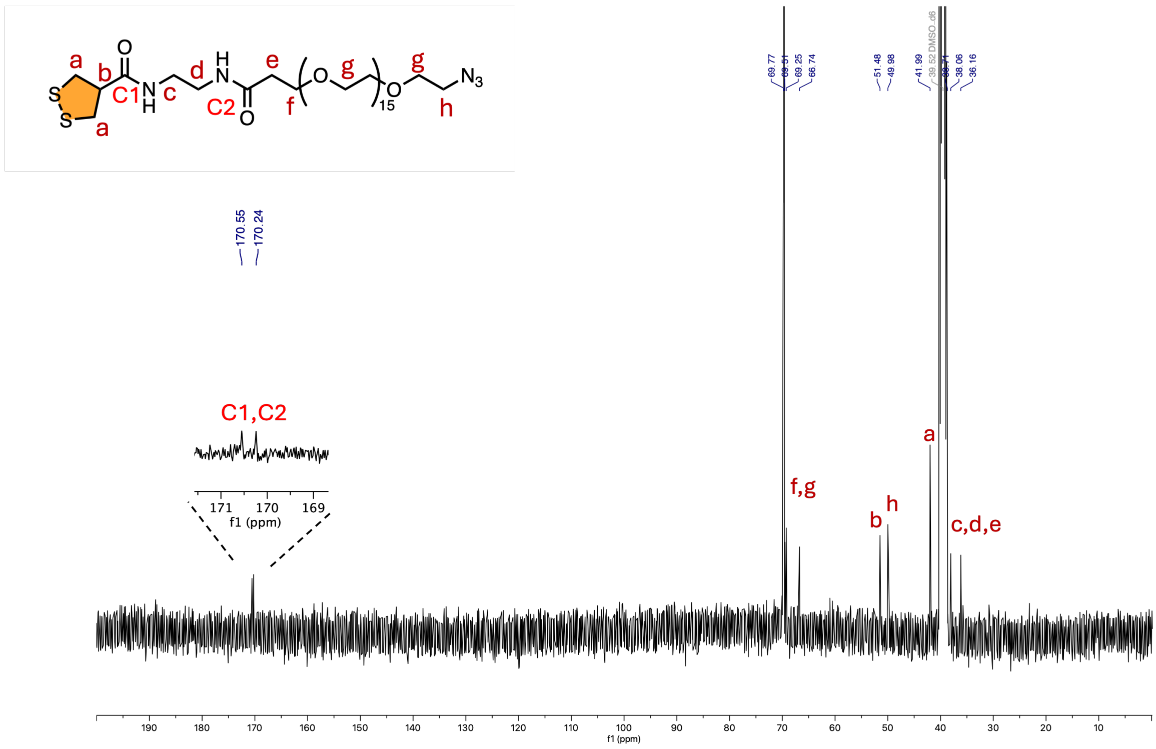

Figure S59. 101 MHz ^{13}C NMR spectrum of compound **31** in DMSO-d_6.

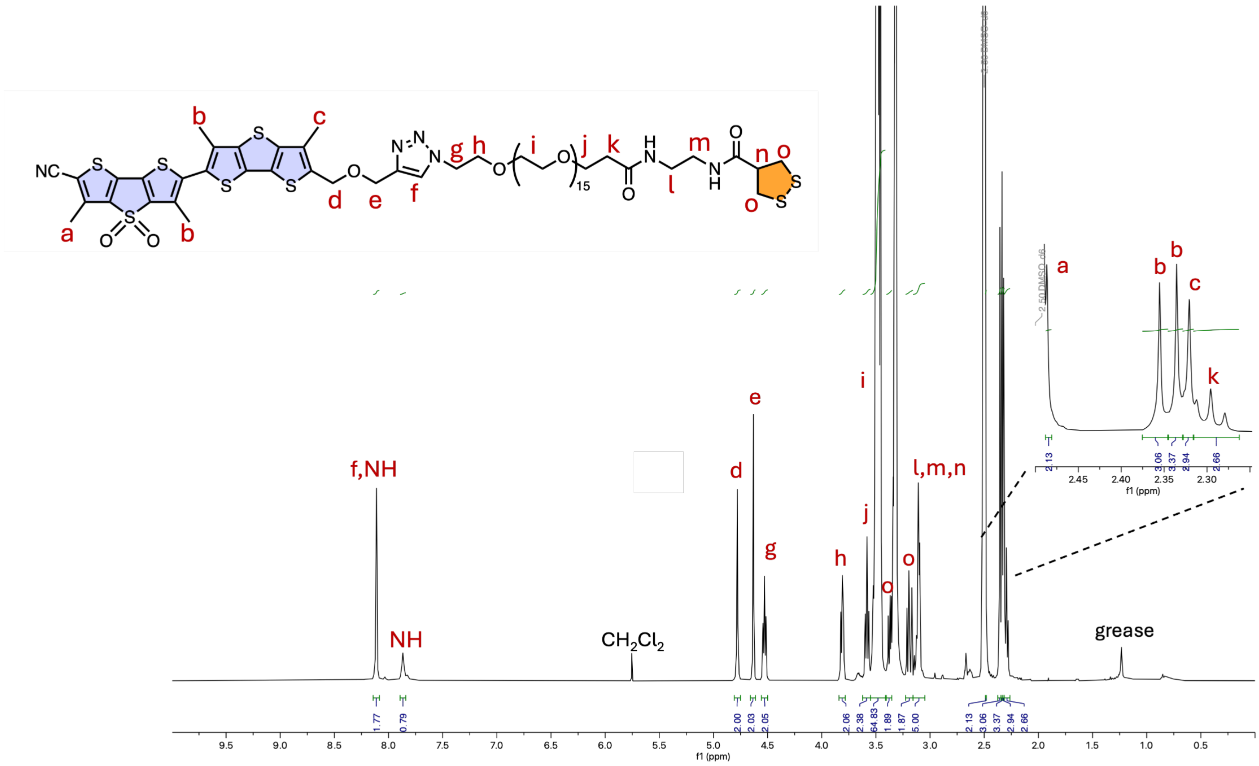

Figure S60. 500 MHz ^{1}H NMR spectrum of compound **11** in DMSO-*d*6.

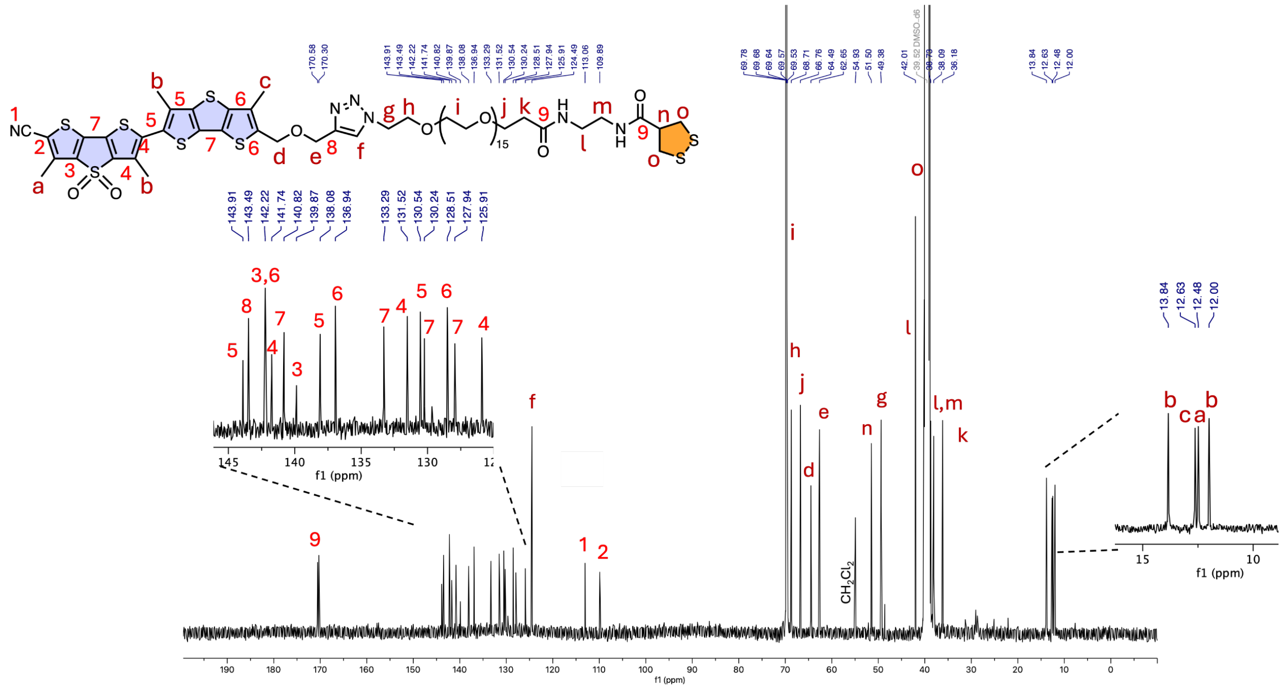

Figure S61. 126 MHz ^{13}C NMR spectrum of compound **11** in DMSO-*d*6.

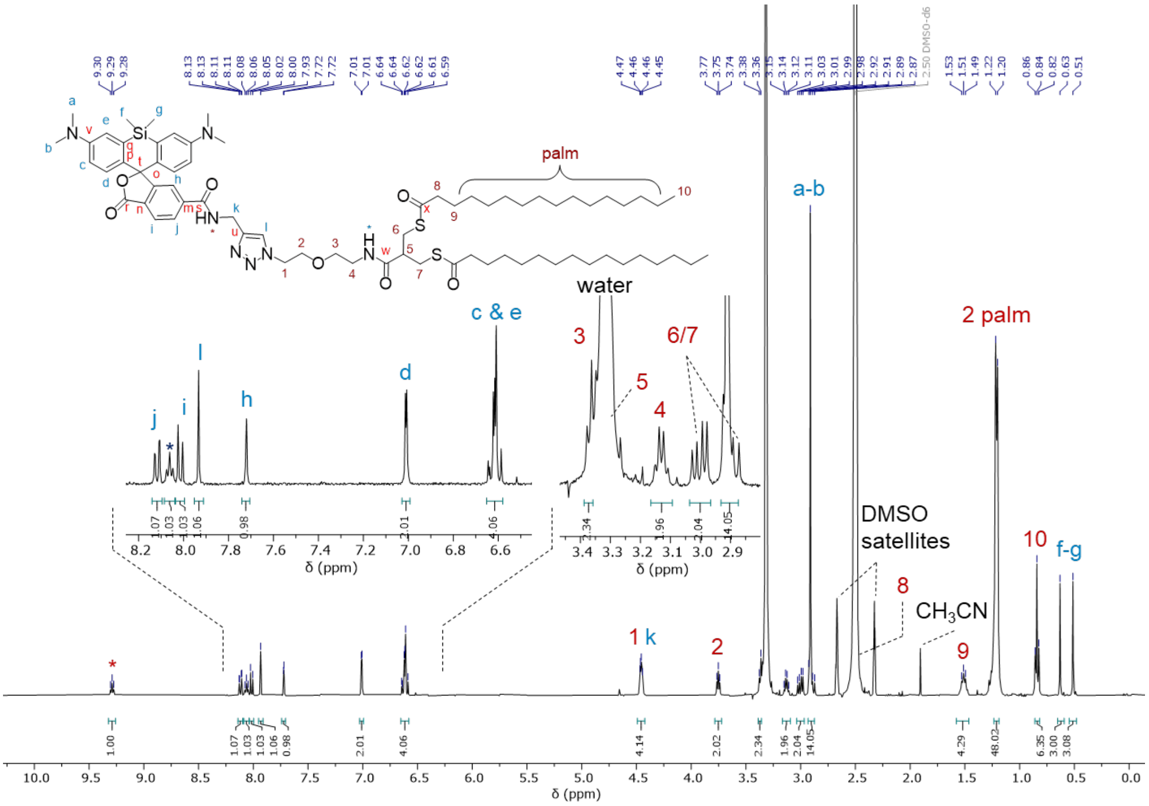

Figure S62. 500 MHz ^{1}H NMR spectrum of compound **2** in DMSO-d_6.

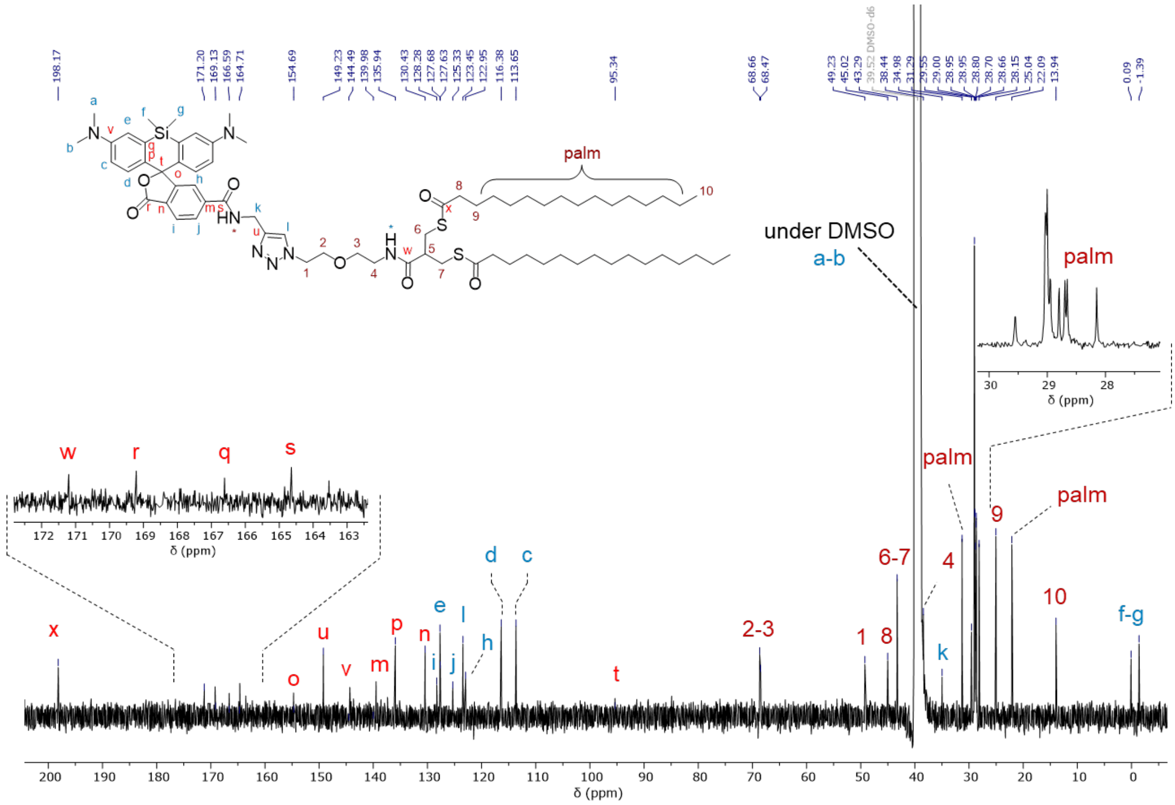

Figure S63. 126 MHz ^{13}C NMR spectrum of compound **2** in DMSO-d_6.
